# Biomimetic All-Wood Sponge for the Co-Generation of Adsorption-Based Atmospheric Water Harvesting and Hydrovoltaic Power Generation

**DOI:** 10.34133/research.1195

**Published:** 2026-03-24

**Authors:** Haoyu Ma, Shengnan Li, Shaowei Wang, Weisheng Yang, Jingquan Han

**Affiliations:** National Key Laboratory for the Development and Utilization of Forest Food Resources, Co-Innovation Center of Efficient Processing and Utilization of Forest Resources, College of Materials Science and Engineering, Nanjing Forestry University, Nanjing 210037, China.

## Abstract

Atmospheric water harvesting (AWH) has garnered widespread attention for the alleviation of freshwater scarcity. However, AWH still presents challenges in simultaneously achieving rapid adsorption–desorption, low-energy regeneration, and environmentally sustainable development. Herein, a biomimetic all-biomass double-layer sponge is prepared to spatially separate moisture capture from solar-driven desorption while integrating hydrovoltaic power generation (HG) for the co-production of water and electricity. Inspired by tree transpiration, the carboxylated wood scaffold loaded with LiCl mimics root-like moisture uptake, while the recycled demethylated lignin coated on the upper layer serves as a photothermal evaporation layer that drives efficient solar-induced desorption analogous to leaf-mediated sunlight absorption. The biomimetic double-layer structure enables efficient moisture absorption and desorption, with vertical humidity gradients guiding water through directional fiber microchannels and generating sustained flow potentials. Under 60% relative humidity (RH), 25 °C, and 1-sun illumination, the open-circuit voltage (*V*_oc_) of the double-layer wood sponge is ~430 mV, with a short-circuit current (*I*_sc_) of ~6.3 μA and water uptake capacity of ~1.45 g g^−1^, maintaining stable properties over multiple adsorption–desorption cycles. Under simulated conditions outdoors, freshwater can be effectively harvested, while the generated electricity can power lighting or be stored in capacitors to supply commercial electronic devices. In this strategy, the natural cellulose–lignin structure within wood is restructured and repurposed into a low-cost, sustainable platform that supplies both freshwater and environmental energy, providing a scalable pathway for efficient collection of water resources in resource-constrained regions.

## Introduction

Freshwater scarcity remains an urgent global challenge, particularly in arid and remote regions lacking centralized infrastructure [1,2]. Substantial amounts of water vapor are stored in the atmosphere, forming a decentralized and underutilized water source [3,4]. Atmospheric water harvesting (AWH) has emerged as a promising strategy for alleviating water constraints [5,6]. Adsorption-based atmospheric water harvesting (SAWH) has attracted particular interest because it can operate across broad ambient humidity conditions and enable day–night water capture [7]. A range of sorbent materials, including modified metal–organic frameworks (MOFs) [8,9], hygroscopic hydrogels [10,11], carbon-based sorbents [12,13], and salt-loaded porous matrices [14,15], have demonstrated substantial water uptake capabilities. Rapid sorption–desorption behavior has also been demonstrated under controlled cycling protocols in recent reports [16]. Yet, it remains challenging to reconcile high uptake, low-energy regeneration, and material sustainability within a single, deployable architecture. In previously reported systems, the adsorption and desorption functions are stacked and hybridized, with limited synergistic effect between functional components [17,18]. The hybrid structure restricts rapid moisture absorption and release, discouraging water collection under low-energy conditions. Furthermore, most high-performance sorbents are derived from petrochemical feedstocks, requiring high production costs and posing environmental burdens during disposal [8,19,20]. Moreover, existing SAWH strategies still require external energy input (such as electric heating) during desorption [21,22], limiting sustainability and scalability. The existing SAWH systems remain primarily single-function and have yet to demonstrate integrated operation with renewable energy harvesting.

Hydrovoltaic power generation (HG) presents a promising pathway to coupling SAWH with electrical energy harvesting by converting adsorption- and evaporation-driven ion transport into electrical output. Recent reports on evaporation-driven or humidity gradient-driven HG systems demonstrate the feasibility of simultaneously recovering water resources and low-grade energy [23,24]. Related biomass-based energy-harvesting concepts have also been explored [25–27]. For example, Yu et al. reported a lignin–cellulose paper for raindrop-driven electricity generation, which relies on droplet impact-driven ion transport [28]. However, practical SAWH and HG co-generation systems are constrained by material and structural design limitations. The SAWH materials (e.g., MOFs [8,29] or aerogels [10,14]) and HG materials (e.g., polymeric materials [30,31] or conductive porous carbons [32,33]) typically form heterogeneous assemblies with poor interfacial compatibility, leading to increased resistance to mass and charge transport. The structural complexity and cost are increased by multi-material module assembly, while the dependence on nonrenewable components often contradicts the original intent of low-carbon sustainable development. What remains missing is a genuinely coupled platform in which water capture, regeneration, and ion-to-electric transduction are co-designed rather than assembled as discrete modules. Therefore, there is an urgent need for a renewable materials platform intrinsically capable of coupling SAWH with the HG process.

Wood, as a renewable and biodegradable biomass, offers distinct advantages for SAWH and energy conversion. The hierarchical and anisotropic pore network is inherently suited for vapor adsorption and directional transport [34]. The cellulose framework can be chemically modified to regulate surface wettability and interfacial charge, enabling wood to serve as a functional substrate for coupling SAWH and HG [35–37]. In parallel, sustainable oxidation and carboxylation routes developed for nanocellulose were shown to introduce surface carboxyl groups under environmentally benign conditions, offering a robust basis for interfacial charge engineering in cellulose-based architectures [38,39]. With the natural capacity for photothermal conversion, lignin can be extracted from wood and reutilized as an efficient photothermal desorption component in SAWH systems [40], allowing solar-driven desorption without external electrical heating. The cellulose framework and lignin provide a fully biomass-based platform capable of moisture adsorption, solar-driven desorption, and hydrovoltaic electricity generation. However, the native wettability, porosity, and charge characteristics of natural wood (NW) remain insufficient to simultaneously meet the requirements of rapid vapor absorption, efficient solar-driven desorption, and effective water collection. Moreover, without targeted modification, the inherent interfacial charge density of NW is typically insufficient to generate high hydrovoltaic output [41–43]. Most current wood-based studies focus on only a single functionality (e.g., solely as a sorbent or as a hydrovoltaic substrate) [44,45] and have yet to realize synergistic optimization of water-harvesting efficiency and electrical energy generation.

Inspired by tree transpiration, a fully bio-based double-layer moisture-absorbing photothermal wood sponge (PHW) was constructed to integrate SAWH with HG. A delignified and carboxylated wood scaffold served as the structural substrate, retaining vertically microchannels for directional mass transport. The lower “root-like” layer was impregnated with LiCl to impart a moisture-absorbing property, while the upper “leaf-like” surface was coated with recycled and modified lignin to function as a photothermal layer for solar-driven desorption. Spatially separating adsorption and photothermal evaporation established the vertical humidity gradient that guided the transport of moisture and ions through the microchannel network, generating a sustained streaming potential during the adsorption–desorption cycle. Under 60% RH and 25 °C, the PHW delivers a *V*_oc_ of 430 mV, an *I*_sc_ of 6.3 μA, and a water uptake capacity of 1.45 g g^−1^ under 1-sun illumination. The performance remained stable over repeated sorption–desorption cycles, demonstrating the structural robustness and long-term reliability of the PHW system. By consolidating sorption, solar regeneration, and ion-to-electric transduction within a monolithic all-wood scaffold, PHW minimizes interfacial transport penalties while harnessing operational humidity/ion gradients to sustain directional water–ion flux and stable streaming-potential output for concurrent water harvesting and HG.

## Results and Discussion

### Biomimetic bilayer strategy for integrated water-energy harvesting

**Fig. 1.**
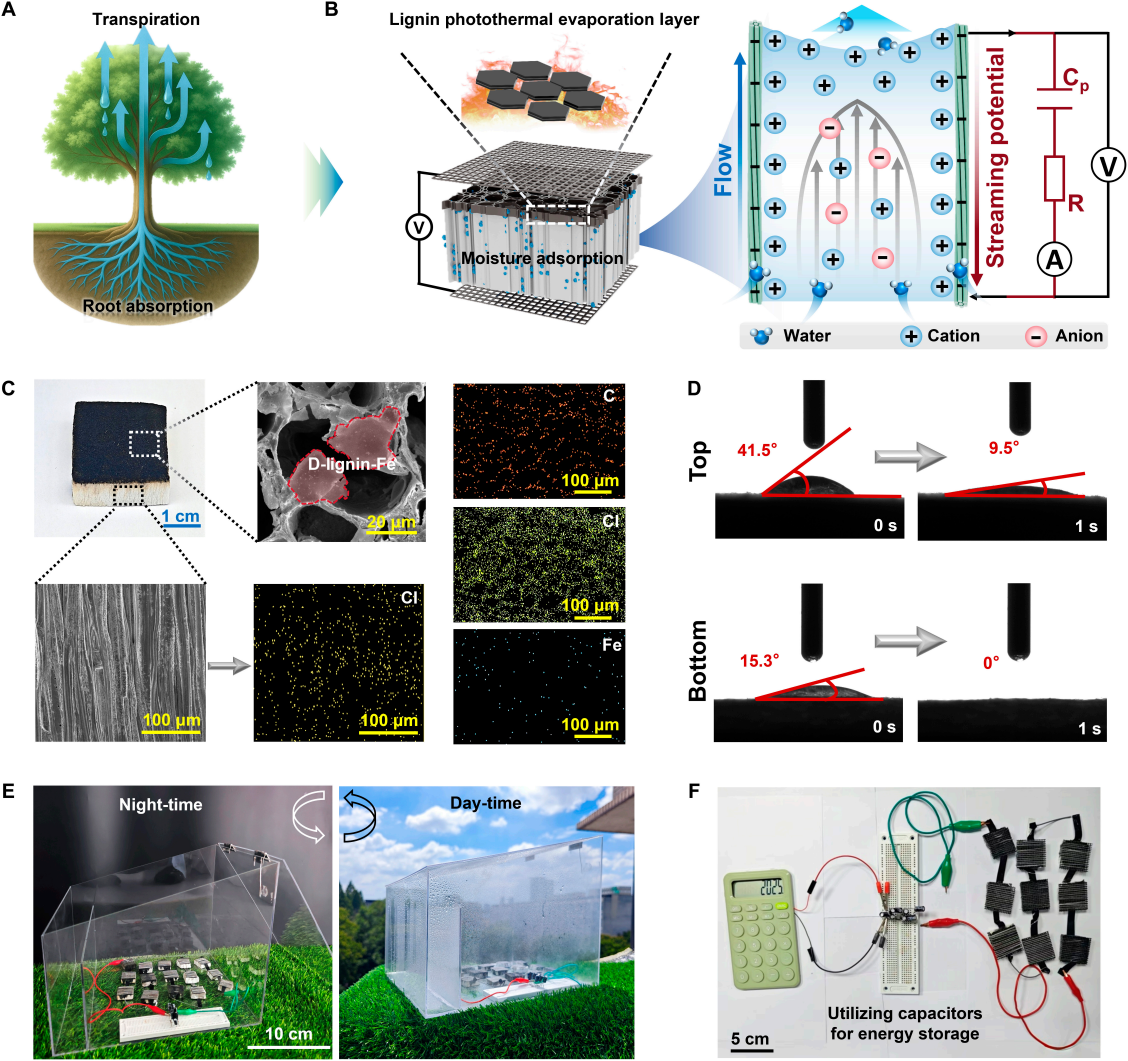
Moisture absorption and hydrovoltaic mechanisms of PHW. (A) Schematic illustrating water absorption and transport inspired by tree transpiration. (B) Layered hygroscopic-photothermal architecture of PHW and its hydrovoltaic mechanism. (C) SEM images and elemental mappings of the cross-section and longitudinal section, with a photograph of PHW. (D) Dynamic contact angle of upper and lower surfaces. (E) Photograph of the outdoor AWH collector used for field-relevant tests. (F) Commercial calculator powered by the electrical energy stored in the capacitor.

Inspired by the spatial separation of root uptake and leaf transpiration in trees, a biomimetic bilayer all-wood sponge was constructed to couple sorption-based AWH with HG (Fig. 1A and B). After delignification and carboxylation, the wood scaffold retained the vertically aligned microchannels, providing a natural framework for directional water and ion transport (Figs. S1 and S2). Removal of lignin and the introduction of carboxyl groups were verified by Fourier-transform infrared spectroscopy (FTIR), x-ray photoelectron spectroscopy (XPS), and quantitative compositional analysis (Figs. S3 to S6 and Tables S1 and S2). The resulting carboxylated wood sponge (CW) exhibited enhanced hydrophilicity and accelerated capillary water transport (Figs. S7 and S8). To enhance atmospheric water uptake, hygroscopic LiCl was incorporated into the CW substrate, yielding a hygroscopic wood sponge (HW) capable of root-like moisture absorption under ambient humidity. X-ray diffraction (XRD) analysis revealed LiCl crystal diffraction peaks while retaining the characteristic cellulose peaks, indicating that the cellulose crystalline structure was retained (Fig. S9). Scanning electron microscopy (SEM) images further confirmed morphological preservation, with the fibrous framework and anisotropic microchannel architecture maintained after LiCl incorporation (Fig. S10).

To complete the tree-inspired “leaf” function, a photothermal regeneration layer was introduced on the upper surface of HW. Lignin released into the deep eutectic solvent (DES) liquor was recovered via an antisolvent precipitation route, thereby enabling lignin recycling and supplying a biomass-derived photothermal precursor (Fig. S11). The recovered lignin was subsequently demethylated to enrich phenolic –OH (Figs. S12 and S13) and subsequently coordinated with Fe^3+^ to form the D-lignin–Fe photothermal complex. The photothermal layer was deposited by drop-casting. During deposition, partial downward infiltration into the porous cellulose network was driven by gravity and capillary action, producing a narrow interpenetration zone at the bilayer interface (Fig. S14). This transition helped stabilize the through-thickness wettability asymmetry and supported the formation of a vertical moisture gradient for directional transport. The hygroscopic and photothermal components were uniformly distributed in the anisotropic channels and on the upper surface of PHW (Fig. 1C and Fig. S15). Due to the biomimetic layered strategy, PHW exhibited remarkable asymmetric wettability, establishing the construction of vertical moisture gradients to facilitate directional transport (Fig. 1D). Outdoor test validated the dual functionality: efficient atmospheric water capture during high-humidity nighttime conditions, and solar-driven interfacial evaporation for freshwater collection during the day (Fig. 1E). Integrated with carbon mesh electrodes, the modular PHW-HG units enabled scalable water-energy co-generation capabilities, enabling direct power supply to electronic devices or energy storage via capacitors for subsequent reuse (Fig. 1F and Movie S1). These results confirmed the potential of the PHW as a self-sustaining, multifunctional platform for AWH and ambient energy generation.

### AWH performance

**Fig. 2.**
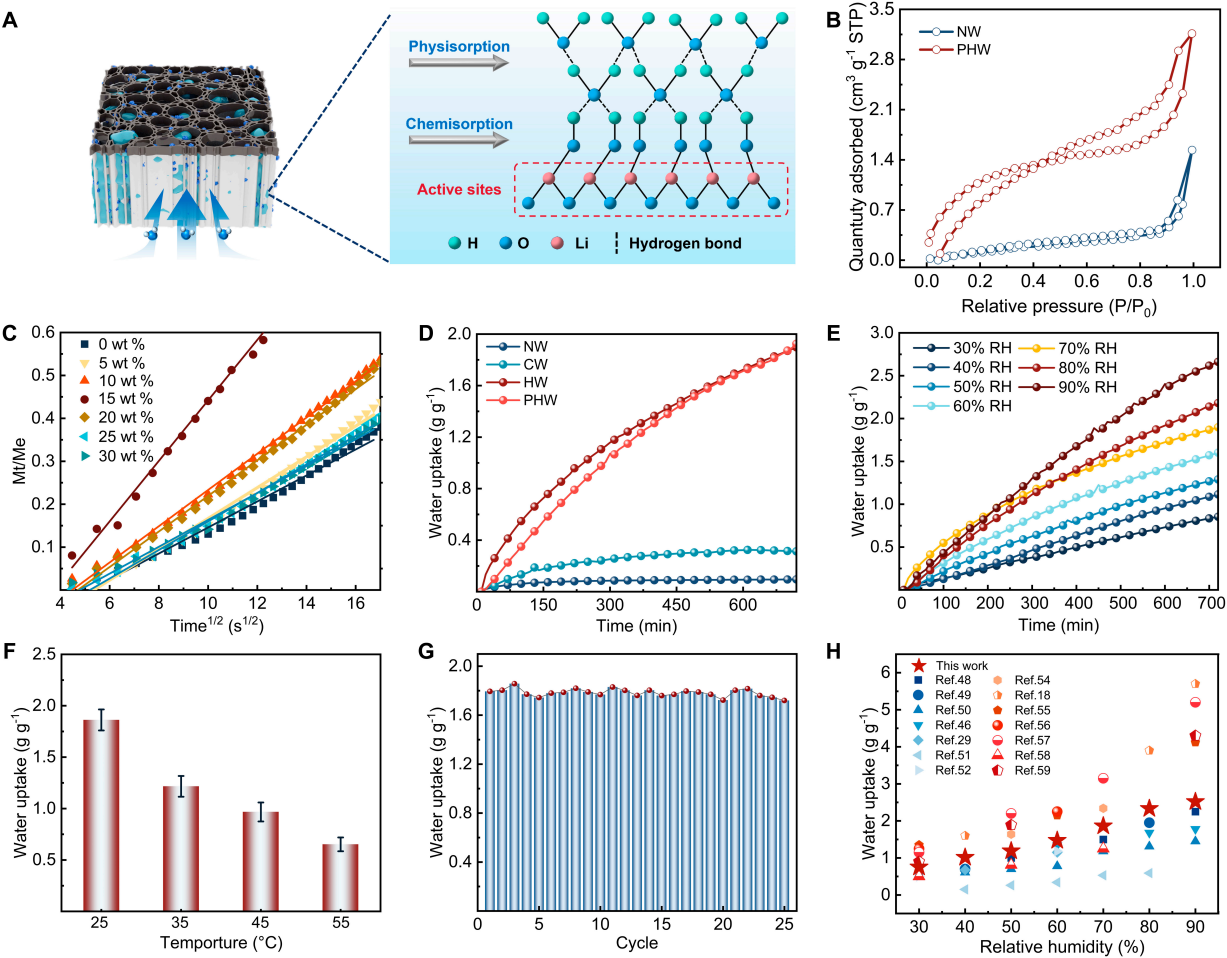
Moisture absorption mechanism and performance of PHW. (A) Schematic illustration of the multilayer water adsorption mechanism. (B) N_2_ adsorption–desorption isotherms of NW and PHW. (C) Fickian diffusion fitting of water sorption data at 70% RH for PHWs impregnated with different LiCl concentrations. (D) Water uptake kinetics of NW, CW, HW, and PHW under 70% RH. (E) Water uptake profiles of PHW under various RH conditions (30% to 90% RH). (F) Hygroscopic capacity of PHW under 70% RH at different temperatures (25 to 55 °C). (G) Cyclic stability of PHW during consecutive adsorption–desorption cycles at 70% RH. (H) Comparison of water uptake capacities of PHW with previously reported AWH sorbents under varying RH conditions.

To elucidate the moisture-uptake behavior of PHW, the sorption mechanism was systematically analyzed. The synergistic interaction of hydrophilic groups (–OH, –COOH) and LiCl promoted hydrogen bonding and hydration interactions with water, producing a dual-mode adsorption composed of surface physical adsorption and chemical adsorption (Fig. 2A) [46,47]. Nitrogen adsorption–desorption isotherms confirmed a markedly increased specific surface area and enhanced mesoporosity compared with NW, indicating extensive structural reorganization induced by delignification and functionalization treatment (Fig. 2B, Fig. S16, and Table S3).

The effect of hygroscopic salt loading on sorption kinetics was evaluated by comparing PHW samples impregnated with different LiCl concentrations at 70% RH (Fig. 2C). Fitting the uptake curves with a Fickian diffusion model ($\frac{M_{t}}{M_{e}}=A\sqrt{t}$) revealed a nonmonotonic dependence on LiCl loading: Diffusion was accelerated within a moderate loading range, whereas the effective diffusion coefficient decreased at excessive LiCl loading (Fig. S17 and Table S4). This reduction at high salt loading was attributed to LiCl aggregation within the pore space, which partially occluded voids and micro-/nanochannels (e.g., cell cavities and interstitial spaces) and suppressed water-vapor penetration and internal moisture diffusion (Figs. S18 and S19). Among all samples, PHW impregnated with 15 wt % LiCl provided the best balance between sorption rate and pore accessibility. Cross-sectional SEM images of the hygroscopic layer in PHW revealed a retained, vapor-accessible microchannel network after functionalization and LiCl loading (Fig. S20), supporting the structural basis for rapid sorption transport and cycling stability. PHW exhibited substantially higher uptake and faster sorption kinetics than NW, CW, and HW, reflecting the synergistic contribution of the hierarchical porosity, surface functionalities, and salt incorporation (Fig. 2D and Table S5). When benchmarked against commercial silica gel, PHW exhibited a faster uptake response and a higher moisture uptake, highlighting the practical advantage of its sorption kinetics (Fig. S21). Under the standard cyclic sorption–desorption protocol used in this work, no visible brine seepage was observed. Under prolonged continuous adsorption at 70% RH (>24 h), however, leakage was detected (Figs. S22 and S23), delineating a practical operating time for stable salt confinement under continuous sorption. To quantify the contribution of the deliquescent salt, a LiCl-free PHW control was evaluated under identical conditions (the same delignified–carboxylated scaffold with the same top coating, but without LiCl impregnation). Moisture uptake dropped sharply (Fig. S24), indicating that hygroscopicity was dominated by LiCl deliquescence. The carboxylated wood scaffold mainly served as a porous, hydrophilic host that confined the salt and maintained vapor-accessible transport pathways. Thus, the hierarchical channels enabled rapid transport and cycling stability, while LiCl provided the dominant sorption capacity. PHW maintained strong water uptake over a broad humidity range (30% to 90% RH), reaching ~2.5 g g^−1^ at 90% RH and maintaining effectiveness at moderate humidity (Fig. 2E, Fig. S25, and Table S6). Water uptake decreased with increasing temperature, consistent with a temperature-shifted vapor–sorbent equilibrium that reduces the retention of adsorbed water at a given RH (Fig. 2F). Under repeated sorption–desorption cycles at 70% RH, PHW retained its sorption capacity, demonstrating the excellent reversibility and structural stability (Fig. 2G). Macroscopic integrity was also retained after sorption–desorption cycling, as evidenced by the post-cycling photographs and a simple load-bearing demonstration (Figs. S26 and S27). Compared with previous AWH materials [18,29,46,48–59], PHW exhibited competitive performance, particularly at moderate humidity conditions where many conventional sorbents demonstrate diminished efficiency (Fig. 2H and Table S7). It should be noted that several LiCl-based composite sorbents and recently developed MOFs can achieve higher peak gravimetric water uptake. Such systems were predominantly designed as single-function sorbents, and performance was often reported primarily by peak capacity. PHW was developed under a different design logic, where sorption is leveraged as a functional driver rather than a stand-alone metric. Within a unified wood scaffold, it enables directional moisture transport, ion migration, and concurrent hydrovoltaic electricity generation.

### Photothermal desorption behavior

**Fig. 3.**
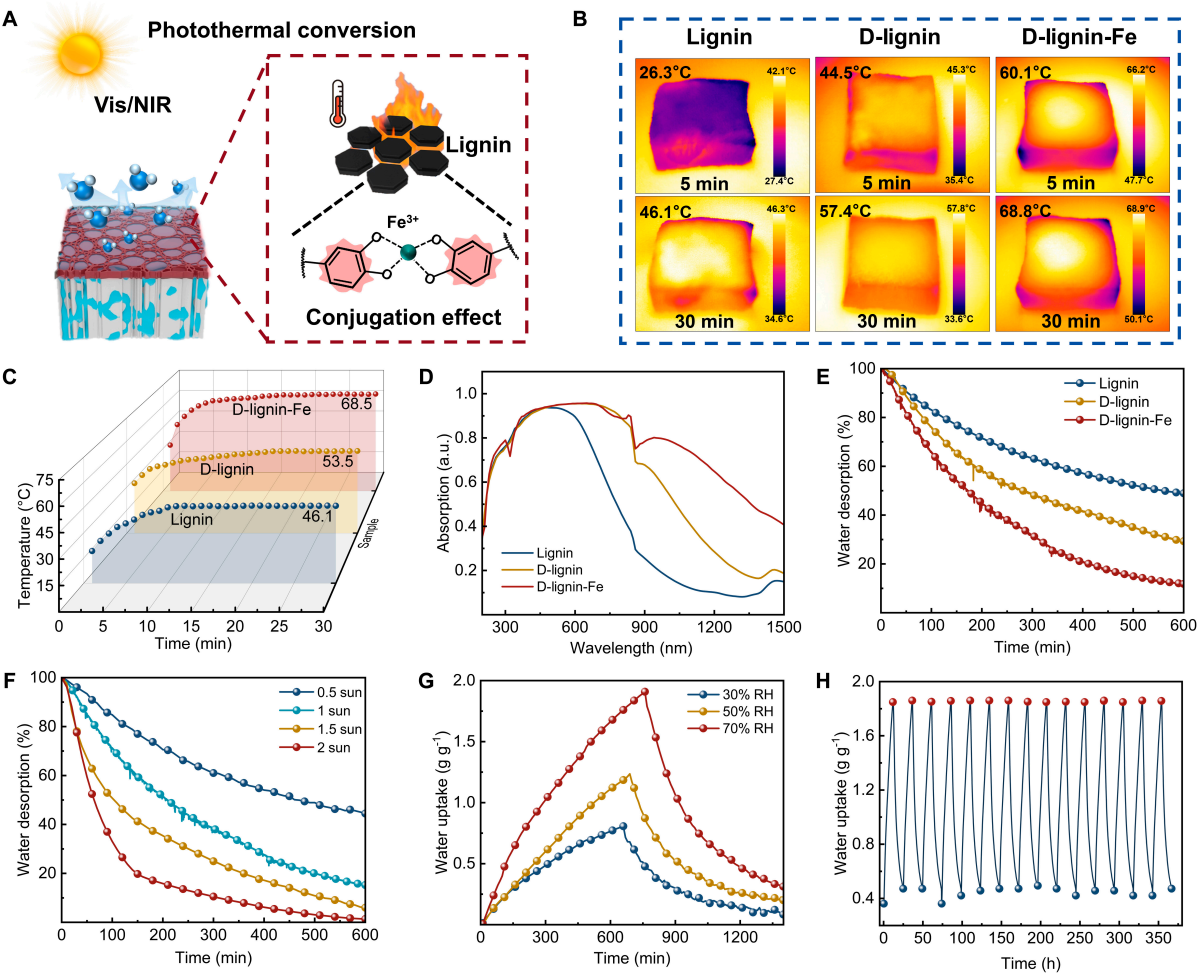
Photothermal desorption performance. (A) Schematic illustrating the photothermal conversion mechanism of D-lignin–Fe complex on PHW. (B) Infrared thermal images of PHWs loaded with lignin, D-lignin, and D-lignin–Fe under 1-sun illumination. (C) Surface temperature evolution of different lignin-based coatings under 1-sun illumination. (D) UV–vis–NIR absorption spectra of lignin, D-lignin, and D-lignin–Fe. (E) Water desorption kinetics of PHW functionalized with different types of lignin under 1-sun illumination. (F) Dynamic desorption curves of PHW under varying solar intensities from 0.5 to 2 suns. (G) Moisture-uptake performance of PHW at different relative humidities (30% to 70% RH), followed by solar-driven desorption under 1-sun illumination. (H) Cycling performance of PHW, with moisture absorption performed at 70% RH and desorption under 1-sun illumination.

To enhance photothermal desorption performance in solar-driven AWH systems, the lignin-derived photothermal coating was modified (Fig. S28). Demethylated lignin was coordinated with Fe^3+^ to form D-lignin–Fe complexes, in which metal–π and π–π interactions were introduced to enhance charge-transfer character and broaden absorption, thereby strengthening the photothermal response (Fig. 3A). Infrared thermal imaging confirmed the enhanced photothermal behavior of the D-lignin–Fe coating, which reached higher surface temperatures under irradiation (Fig. 3B and C). This enhancement correlated with the ultraviolet (UV)–visible (vis)–near-infrared (NIR) spectra, where the broadened and intensified absorption, particularly in the NIR region, indicated the improved light-harvesting capability (Fig. 3D) [40,60]. Solar-driven desorption was enabled by placing the photothermal lignin coating on the upper surface of PHW. Under irradiation, absorbed light was converted into local heat at the air–solid interface, and regeneration was promoted primarily by interfacial evaporation rather than bulk heating. Water was thus preferentially removed from the near-surface region, while a humidity gradient was maintained across the thickness, driving moisture transport from the hygroscopic layer upward through the aligned microchannels during regeneration. Under solar illumination, PHW coated with D-lignin–Fe exhibited rapid and nearly complete water desorption, supporting its suitability for SAWH through efficient photothermal desorption (Fig. 3E). A coating mass of 60 mg provided the best desorption performance (Fig. S29). Below this loading, insufficient photothermal input limited the desorption efficiency, whereas excessive coating partially obstructed microchannels and restricted vapor transport, reducing the desorption rate (Fig. S30). The desorption rate could be modulated by increasing irradiance (0.5 to 2 suns), indicating adaptability to fluctuating sunlight conditions (Fig. 3F). Continuous adsorption–desorption testing further evaluated cycling durability. PHW achieved 0.75 to 1.86 g g^−1^ water uptake under ambient RH (30% to 70%), followed by rapid release under 1-sun illumination (Fig. 3G). Notably, the D-lignin–Fe coating maintained stable and reproducible adsorption–desorption performance over multiple cycles, with no observable macroscopic damage (Fig. 3H), supporting the robustness for extended outdoor operation.

### Hydrovoltaic generation of the PHW-HG device

**Fig. 4.**
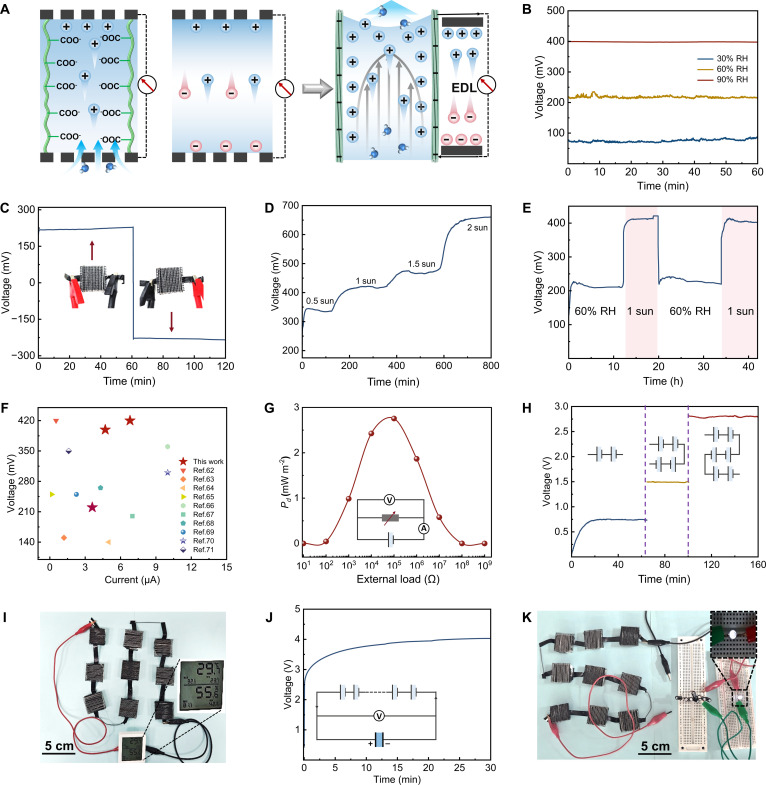
Coupled moisture absorption and photothermal evaporation hydrovoltaic performance. (A) Schematic of the hydrovoltaic mechanism. (B) *V*_oc_ during moisture absorption at different RHs (30%, 60%, 90% RH). (C) Reversal of voltage polarity by reversing electrode connection. (D) Enhanced voltage response under increased illumination (0.5 to 2 suns). (E) Simulating the day–night cycle electrical output property during alternating moisture absorption (60% RH) and desorption (under 1 sun). (F) Voltage–current comparison of PHW-HG with reported hydrovoltaic devices. (G) Output power density (*P*_d_) versus external load resistance. (H) Scalable voltage output of multiple modules connected in series (under 1 sun). (I) Hydrovoltaic performance powering a commercial thermohygrometer. (J) Series-connected modules charging a capacitor and (K) capacitor-stored energy powering a light-emitting diode.

During sorption–desorption cycling, negatively charged –COO^−^ sites along the aligned PHW channels promoted cation enrichment at the channel–water interface and within the electric double layer. Combined with asymmetric wettability and a directional moisture flux, this ion-selective redistribution sustained interfacial charge separation and stabilized electrical double layer (EDL) formation, thereby accumulating a path-integrated flow potential along the transport direction (Fig. 4A). Zeta-potential measurements served as a qualitative indicator for charge asymmetry across the bilayer (Fig. S31). The hygroscopic bottom layer showed a more negative potential (−18.4 mV) than the D-lignin–Fe top layer (−11.2 mV), consistent with partial charge neutralization due to Fe^3+^ coordination. The ζ-potential contrast (~7.2 mV) was not interpreted as a stand-alone driving force for the device output. It was used only to indicate the relative interfacial charge magnitude under simplified measurement conditions. Under operation, a continuous, directional moisture flux along the aligned microchannels sustained the macroscopic voltage. This effect was reinforced by mobile ions from dissociated LiCl. The presence of LiCl in the hygroscopic layer provided a steady reservoir of Li^+^ ions, effectively amplifying the humidity gradient-driven ionic flux and enhancing the resulting flow potential [61]. The high mobility of Li^+^ within cellulose nanochannels further promoted directional ion migration, leading to higher voltage and current outputs. In contrast, the PHW without LiCl exhibited negligible electrical output, highlighting the essential role of LiCl in converting ambient humidity into sustained ionic transport and electrical output (Fig. S32). As relative humidity increased, enhanced moisture flux drove more water molecules to migrate through the cellulose channels, promoting ion migration. Strengthened interfacial charge separation and the establishment of flow potential led to elevated voltage and current outputs (Fig. 4B and Fig. S33). Stable and reproducible voltage curves over repeated sorption cycles revealed outstanding reversibility and operational stability (Fig. S34). The polarity reversal caused by electrode polarity inversion further confirmed that electricity generation arose from directed ion migration and asymmetric interfacial charge distribution (Fig. 4C). Photothermal heating further boosted the hydrovoltaic output by increasing evaporation flux and ion transport (Fig. 4D and Fig. S35). During the cycle, the PHW remained partially hydrated, with LiCl primarily existing in a deliquescent state rather than as crystalline aggregates. Accordingly, after multiple adsorption–desorption cycles, the output remained stable (Fig. 4E). Compared with reported hydrovoltaic platforms, PHW-HG was placed in a favorable voltage–current regime (Fig. 4F) [62–71]. To probe the impact of deeper dehydration, prolonged continuous illumination was applied to intensify water removal while the hydrovoltaic output was monitored. LiCl crystallization became evident, and the output declined progressively under this harsher condition (Fig. S36). Notably, under practical outdoor operation, solar irradiation is confined to daytime. Accordingly, within a normal day–night cycle, crystallization-induced efficiency loss was not expected to be particularly marked. Under external load conditions simulating real-world applications, a peak power density of ~2.9 mW m^−2^ was achieved at ~10^5^ Ω, demonstrating an optimal balance between current and voltage (Fig. 4G). Modularity was demonstrated by connecting multiple PHW units in series or parallel to achieve multi-level voltage or current outputs (Fig. 4H and Figs. S37 and S38). Capacitor charging tests further validated the practical feasibility. The series modules accumulated several volts within minutes under 1-sun illumination, and the stored energy successfully powered modest electronic devices (Fig. 4H to K and Movie S2). Collectively, the PHW generated continuous electricity through moisture absorption and photothermal desorption, offering scalable output and energy storage capability. These results highlight its potential as a decentralized, environmentally friendly, and geographically adaptable power-generation platform.

### Outdoor demonstration of integrated water-energy supply

**Fig. 5.**
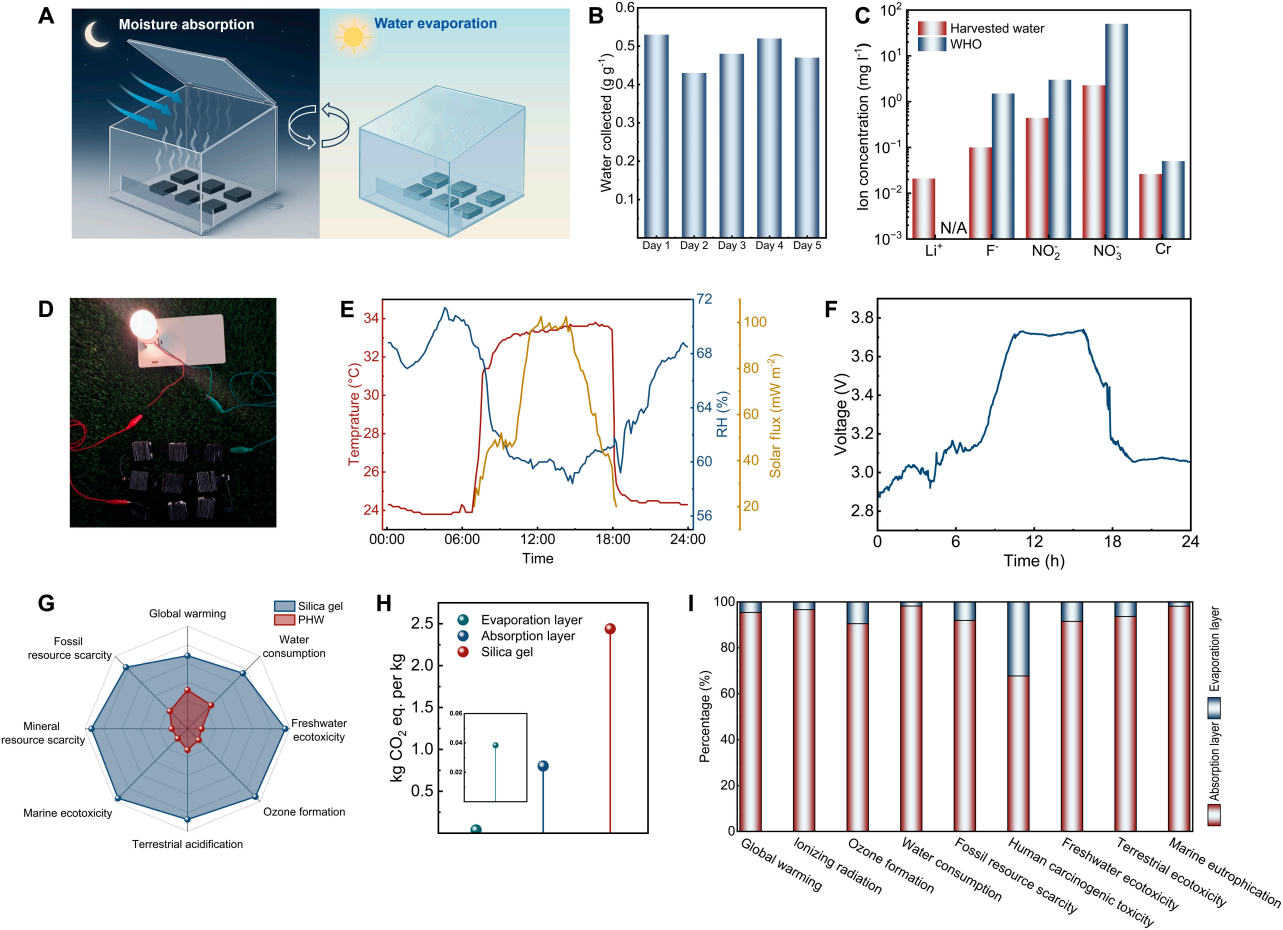
Outdoor water-electricity co-generation and LCA calculation. (A) Schematic of the outdoor day–night operating cycle (night adsorption/daytime regeneration and water collection). (B) Collected water per cycle during 5 day–night run under outdoor conditions. (C) Ionic composition of the harvested water, benchmarked against WHO guideline values where available; species without WHO health-based guideline values are marked accordingly. (D) Commercial lighting illuminated via electricity generated by series-connected PHWs’ hydrovoltaic effect at night. (E) Monitored ambient conditions (temperature, relative humidity, and solar flux) during field testing. (F) Output voltage profile of the PHW-based devices over a 24-h cycle. (G) Radar chart comparing the environmental impact profiles of PHW and silica gel across indicators, including global warming, fossil resource scarcity, and mineral resource scarcity. (H) CO_2_ equivalent emissions of silica gel, along with the moisture-absorbing layer and evaporation layer of PHW. (I) Percentage contribution of the evaporation layer and absorption layer to various environmental impact categories (e.g., global warming and ionizing radiation).

The integrated water–electricity co-generation of PHW was examined under outdoor-style day–night cycling. Moisture was adsorbed during the high-RH nighttime period, followed by solar-driven interfacial evaporation in daylight that regenerated the sorbent and enabled condensate collection (Fig. 5A). To quantify the device-level water yield under repeated outdoor-style operation, the condensate mass collected per cycle was recorded over 5 consecutive day–night cycles (Fig. 5B). A comparable amount of condensate was collected each day, indicating that the sorption–photothermal regeneration–condensation sequence remained reproducible without abrupt decay in water collection. Freshwater production was thus achieved without external electrical input under outdoor conditions. The harvested water was subsequently analyzed for ionic content (Fig. 5C and Table S8). The measured concentrations were compared to World Health Organization (WHO) health-based drinking-water guidelines. Ions without WHO health-based guideline values (e.g., Li^+^) were reported for transparency [72]. Outdoor demonstrations showed that the PHW system can simultaneously harvest atmospheric water and power electronic devices, highlighting the practical applicability (Fig. 5D). To evaluate the real-time response of PHW under natural environmental conditions, ambient temperature, relative humidity, and solar irradiance flux were monitored (Fig. 5E). The output voltage tracked these environmental fluctuations (Fig. 5F). Even under fluctuating weather, the PHW maintained robust hydrovoltaic performance, reflecting the adaptability of the moisture-absorbing photothermal bilayer architecture.

The sustainability of PHW was further evaluated via life cycle assessment (LCA). As wood constitutes the primary raw material, the biogenic carbon sequestered during tree growth was accounted for in the inventory as a carbon credit, thereby reducing the overall greenhouse gas footprint [73]. Compared with moisture-absorbing materials such as silica gel, the all-wood PHW showed lower impacts across multiple categories, with reductions observed in global warming potential, fossil resource consumption, and freshwater consumption (Fig. 5G and H and Table S9). These improvements were attributed to the low-energy processing and renewable nature of wood, together with lignin valorization as a reusable photothermal coating, which reduces reliance on virgin inputs and associated waste streams. Contribution analysis further revealed that the hygroscopic substrate (including LiCl loading and chemical modification) accounted for the majority of the LCA impacts, whereas the photothermal layer contributed a smaller share (Fig. 5I). This indicated that further optimization of substrate preparation offers a promising route to additional environmental impact reduction. In summary, the PHW design not only achieved functional water-energy coupling but also demonstrated that the feedstock selection and processing route constitute a green solution with low environmental impact and carbon sequestration potential.

## Conclusion

A biomimetic, fully biomass-based photothermal-hygroscopic wood sponge was developed to incorporate AWH with HG. Inspired by tree transpiration, the delignified and carboxylated wood scaffold loaded with LiCl supported efficient root-like moisture uptake, while a lignin-derived photothermal coating facilitated solar-driven desorption through interfacial evaporation. Benefiting from the hierarchical porous structure and asymmetric wettability, the PHW exhibited an excellent water uptake of 1.45 g g^−1^ at 60% RH and 25 °C, with rapid moisture release under 1-sun illumination. Meanwhile, continuous hydrovoltaic electricity generation was realized, delivering an open-circuit voltage of ~430 mV and a short-circuit current of ~6.3 μA, with excellent stability over repeated sorption–desorption cycles. LCA further confirmed the environmental benefits of the PHW. As a biomass-based material, wood contributed to carbon sequestration and reduced greenhouse gas emissions, while the recycling of lignin contributed to resource circularity. Compared to petroleum-derived adsorbents, the PHW exhibited substantially lower global warming potential, fossil resource consumption, and freshwater usage. Overall, a renewable, low-cost, and multifunctional decentralized water and energy supply platform was demonstrated, advancing sustainable solutions to address global freshwater scarcity and clean energy demands. Several engineering constraints remain to be addressed. In particular, salt confinement under prolonged high humidity, regeneration-rate sensitivity to ambient vapor-pressure boundary conditions, and durability beyond the present cycling window require further validation. These issues will be addressed in future work by systematically testing the device under controlled humidity/temperature conditions and by improving the wood scaffold design to better retain the salt during long-term operation.

## Materials and Methods

### Materials

Natural balsa (*Ochroma lagopus Swartz*) was purchased from a lumber mill in Shandong, China. Acetic acid (99.5%), oxalic acid (OA) (97%), dichloromethane (99.5%), iron chloride (99.9%), sodium hydroxide (96%), lithium chloride (99.995%), sodium acetate trihydrate (99.5%), chloroacetic acid (98%) and 1,4-dioxane (99.5%) were purchased from Sinopharm Chemical Reagent Co. Choline chloride (ChCl) (98%), boron tribromide (99.99%), chloroacetic acid (98%), and sodium chlorite (80%) were obtained from Shanghai Maclean Biochemical Co. Polyester fiber (PET) with an aperture of 10 mesh was acquired from a merchant in Zhejiang, China. CH-8 ink was purchased from Jujo Chemical Co. Ltd.

### Preparation of carboxylated HW

Natural balsa wood blocks (3 × 3 × 1 cm^3^) were first pretreated in the DES, prepared by mixing ChCl and OA at a mass ratio of 1:1. The mixture was heated to 60 °C under stirring until a transparent liquid was obtained. Wood samples were immersed in the DES (solid–liquid ratio = 1:20) and treated at 100 °C for 3 h. The lignin-rich DES solution was collected for lignin extraction, and the DES-treated wood (DESW) was thoroughly washed with ethanol and deionized water (DIW). A subsequent bleaching process was performed in a mild acetate buffer (pH 4.6) at 80 °C for 12 h. The delignified wood (DW) was rinsed with DIW until a neutral pH was achieved.

To facilitate carboxylation, the DW was solvent-exchanged with ethanol, followed by reaction in a mixture containing chloroacetic acid and NaOH (1:2.6 molar ratio) in a solvent system of 10% DIW and 90% ethanol (v/v). The concentration of NaOH was adjusted to 17 g l^−1^, and the reaction was carried out at 93 °C for 5 h. The carboxylated wood was washed with DIW to remove residual reagents, then frozen at −15 °C for 12 h, and freeze-dried for 48 h to obtain the porous wood sponge. For hygroscopic modification, the wood sponge was immersed for 12 h in LiCl aqueous solutions of varying concentrations (0, 5, 10, 15, 20, 25, and 30 wt %). Samples were then dried at 100 °C for 24 h to yield LiCl-impregnated HWs.

### Extraction and functionalization of DES-derived lignin

Lignin was first extracted from the lignin-rich DES waste solution by adding counter-solvent DIW to reduce lignin solubility. The mixture was centrifuged at 10,000 rpm, and the supernatant was discarded. This washing step was repeated 3 times to fully remove DES residues. The extracted lignin was freeze-dried and tagged as DES-lignin.

For demethylation, 1 g of DES-lignin was dissolved in 100 ml of anhydrous CH_2_Cl_2_. Boron tribromide (1 M in CH_2_Cl_2_, 20 ml) was added dropwise at 0 °C. The reaction mixture was then maintained at room temperature for 24 h and quenched with cold water. The resulting precipitate (D-lignin) was washed with DIW 3 times. For iron coordination, D-lignin (1 g in 20 ml water) was adjusted to pH 12, and 10 ml of 0.5 M FeCl_3_ solution was added. The mixture was stirred for 3 h at room temperature, and the precipitate (D-lignin–Fe) was collected via centrifugation, washed, and dried at 60 °C.

### Loading of lignin-derived photothermal coating

To fabricate lignin-based PHWs, D-lignin–Fe was dissolved in 1,4-dioxane/DIW (4:1, v/v) at a concentration of 25 mg ml^−1^. A volume of 200 μl of the 20 mg ml^−1^ solution was drop-cast onto the surface of HW (a coated area of 9 cm^2^). Upon complete drying, the process was repeated to achieve different lignin loadings.

### Assembly of photothermal-hygroscopic wood sponge HG

The PET mesh was washed in ethanol for 10 min and then thoroughly coated with CH-8 carbon paste. After drying at 80 °C for 2 h, the carbon mesh electrode was obtained and affixed to the top and bottom surfaces of the wood-based photothermal-hygroscopic sponge. The entire assembly was then secured using cotton thread, forming a tightly integrated moisture-electric generator. Finally, the device was electrically connected via external wires and placed under different relative humidity environments, where the generated electrons were collected for subsequent power output evaluation.

### Adsorption/desorption performance test

The hygroscopic performance of the samples was evaluated using a custom-built apparatus (Fig. S39 and Movie S3). Before testing, all samples were dried in a vacuum oven at 100 °C for 10 h. The relative humidity within the sealed chamber was precisely regulated using a commercial humidifier in conjunction with a humidity controller. Solar illumination was simulated using a 300-W xenon lamp. The mass change during the desorption process was recorded using an electronic balance. The water uptake was calculated by the following equation:

$$\text{Water uptake}\;\left(\%\right)=\frac{\left(m_{t}-m_{0}\right)}{m_{0}}\times 100$$ (1)

where *m_t_* is the mass of the aerogels after moisture sorption at different times and *m*_0_ is the initial mass of the aerogels before sorption.

### Diffusion rate calculated via Fick’s second law

The water diffusion coefficient (*D*), which reflects the water transport rate, can be determined via Fick’s second law in the form of a triangular series, as shown in the equation below:

$$\frac{M_{t}}{M_{e}}=\frac{2}{d}\sqrt{\frac{\mathrm{Dt}}{\pi }}$$ (2)

*M_t_* and *M_e_* represent the mass of water uptake, *M_t_* is the mass at time *t*, and *M_e_* is the mass at equilibrium for the adsorption process. *d* is the sample thickness. Experimental values of *M_t_*/*M_e_* were plotted against the square root of time at various RH, along with the linear fit.

### Practical outdoor test

The outdoor water-harvesting experiment was conducted on 2025 June 18, on the roof of the laboratory (118.48 E, 32.04 N). The ambient temperature and relative humidity were monitored in real time using a commercial digital thermo-hygrometer. Solar irradiance was recorded by a photometric solar radiometer (CEL-NP2000-2A, China). Water collection was performed by a custom-designed collector. The harvesting chamber and condenser were constructed from polymeric and other inert components to minimize salt-induced corrosion and to reduce potential ionic contamination from metal components. The ionic composition of the collected water was analyzed via inductively coupled plasma optical emission spectrometry (ICP-OES; iCAP7600, Thermo Fisher Scientific) and ion chromatography (ICS-5000, Thermo Dionex).

### Environmental impact assessment

The environmental impacts of PHW were quantified by performing an LCA using the Simapro software (Netherlands) and the corresponding database.

### Characterization

Chemical functional groups were characterized by FTIR (Bruker VERTEX 80V). Surface elemental composition and chemical states were analyzed by XPS (Shimadzu AXIS UltraDLD), while crystallinity was examined using XRD (Rigaku Ultima IV). Cross-sectional and radial morphologies were observed by field-emission SEM (FE-SEM; JEOL JSM-7600), and elemental distributions were mapped by energy-dispersive x-ray spectroscopy (EDS; Oxford X-Max) at 5 kV. Solution-state nuclear magnetic resonance (NMR) spectra of lignin were acquired on a Bruker AVANCE III 600 MHz spectrometer (Bruker BioSpin). Lignin was dissolved in DMSO-d6 at 60 mg/0.6 ml, and spectra were recorded at 298 K. ^1^H and ^13^C NMR (and 2D heteronuclear singular quantum correlation, if applicable) were collected, with chemical shifts referenced to the residual solvent signal. Cellulose, hemicellulose, and lignin contents were quantified by high-performance liquid chromatography (HPLC) following standard National Renewable Energy Laboratory (NREL) protocols. Carboxyl group content was determined by conductivity titration. The phenolic hydroxyl content of lignin was measured by a Folin–Ciocalteu procedure after dissolution in NaOH, with absorbance read at 760 nm. Specific surface area and porosity were obtained from N₂ adsorption–desorption isotherms (Micromeritics ASAP 2020, Brunauer Emmett Teller method). The zeta potential of the samples was measured with a laser particle size analyzer (Zetasizer Nano ZSE, Malvern, UK). Surface wettability was assessed by contact angle goniometry. Photothermal tests were performed under a solar simulator (HXF300-T3) with irradiance monitored by an optical power meter (CEL-NP2000-2A), and thermal evolution was recorded by an infrared camera (Testo 875-i). Optical absorption of the photothermal coating was measured by UV–vis–NIR spectrophotometry (PerkinElmer Lambda 950, 200 to 2500 nm). The sheet resistance of carbon mesh electrodes was measured using a 4-point probe (RST-9). Electrical signals (voltage and current) were logged in real time by a Keithley DMM6500 data acquisition system (with corresponding control software) at a 1-Hz sampling rate.

## Data Availability

Data will be made available on request.
