# Supplementary material for: Biomimetic All-Wood Sponge for the Co-Generation of Adsorption-Based Atmospheric Water Harvesting and Hydrovoltaic Power Generation
Source: Research (Wash D C). 2026 Mar 24;9:1195. doi: 10.34133/research.1195 (PMC13009533; doi:10.34133/research.1195)
Supplement: Supplementary 1 — Figs. S1 to S39 Tables S1 to S9 Movies S1 to S3 [file research.1195.f1.zip › Supplementary Information.docx]

**Supplementary Information**

**Biomimetic All-Wood Sponge for the Co-Generation of Adsorption-based Atmospheric Water Harvesting and Hydrovoltaic Power Generation**

Haoyu Ma^1^, Shengnan Li^1*^, Shaowei Wang^1^, Weisheng Yang^1^, and Jingquan Han^1*^

^1^ Co-Innovation Center of Efficient Processing and Utilization of Forest Resources, College of Materials Science and Engineering, Nanjing Forestry University, Nanjing, 210037, China.

*Address correspondence to: [hjq@njfu.edu.cn](mailto:hjq@njfu.edu.cn) (J. Han); [lishengnan33@njfu.edu.cn](mailto:lishengnan33@njfu.edu.cn) (S. Li)

**Supplementary Figures**


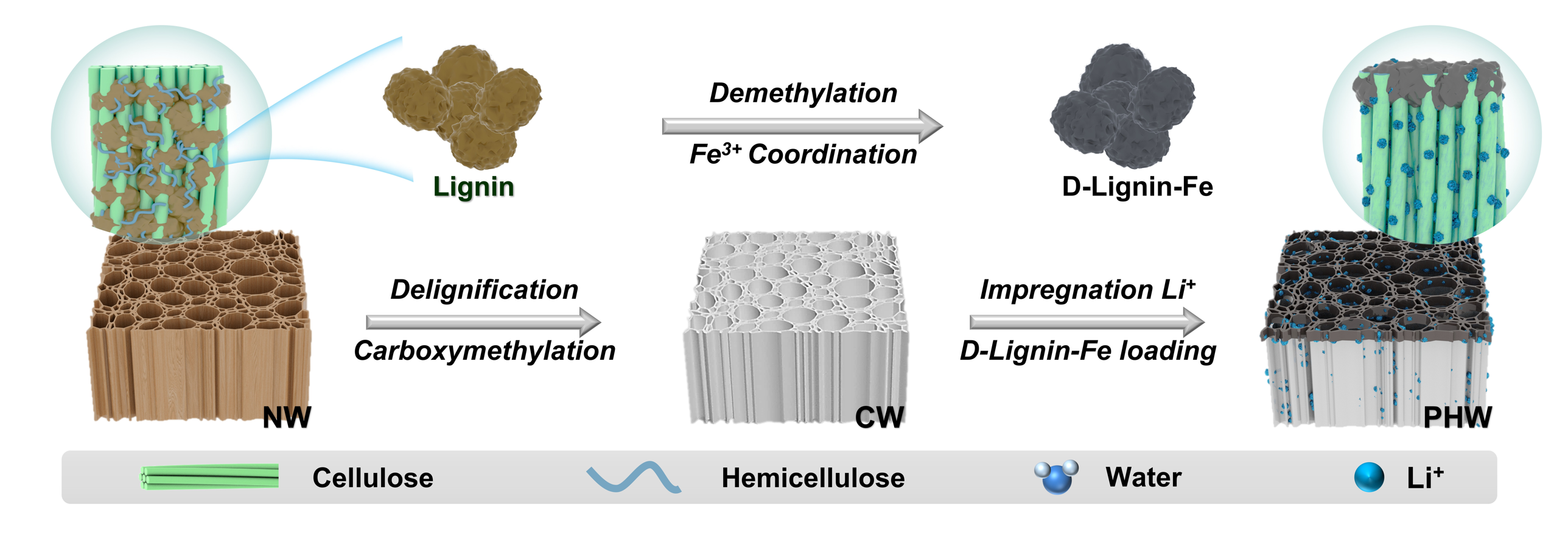


**Fig. S1.** Preparation flowchart of PHW.

NW was delignified and carboxymethylated to produce CW; Lignin underwent demethylation and Fe³⁺ coordination to form D-Lignin-Fe; Subsequently, Li⁺ impregnated and loaded D-Lignin-Fe, ultimately yielding PHW.


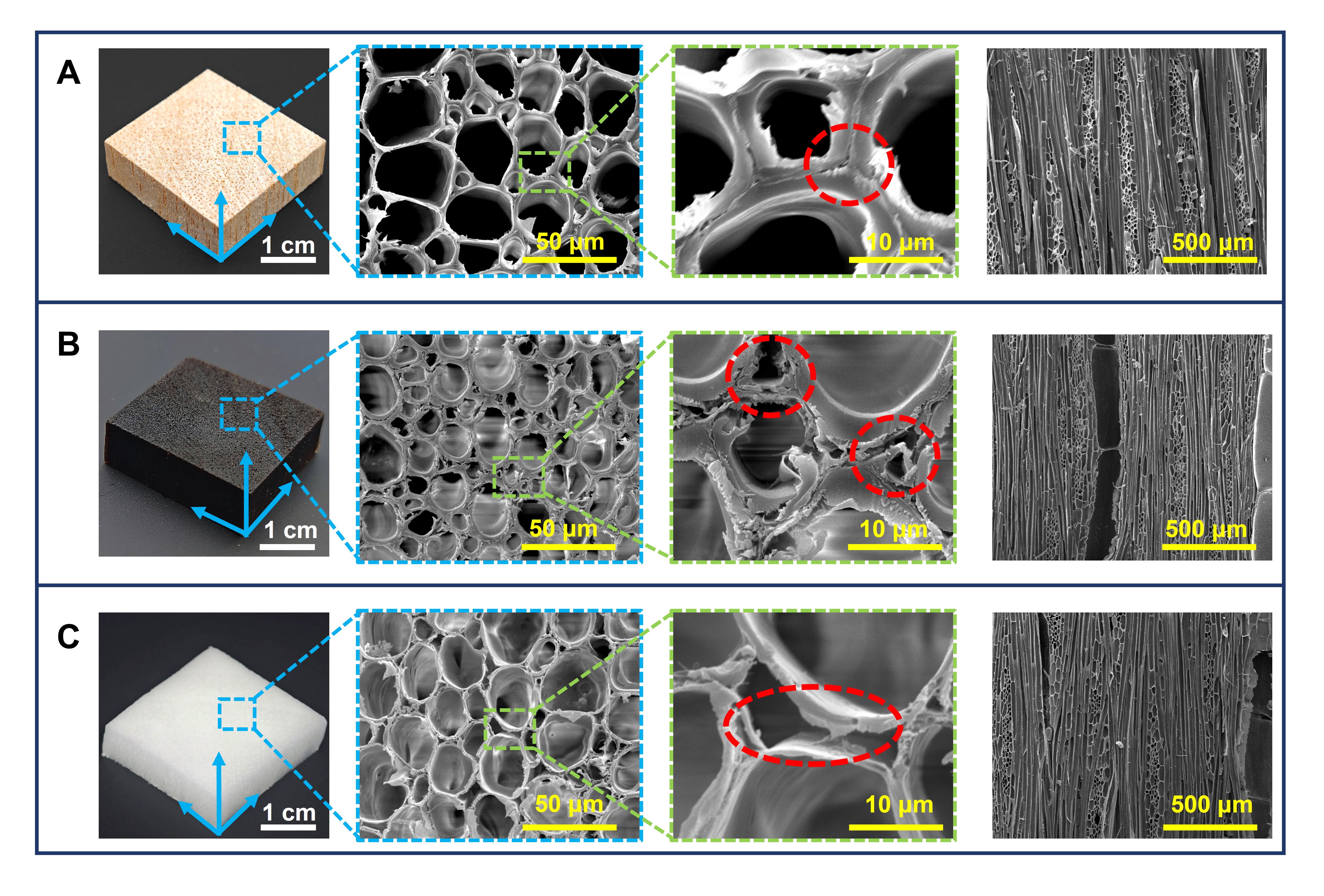


**Fig. S2.** (A) NW, (B) DESW, and (C) DW: Photographs and SEM images of cross-sections and longitudinal sections.

DES-treated wood (DESW) exhibited partial dissolution of lignin and a more porous structure, thereby facilitating subsequent bleaching processes. Bleaching with an acetic acid buffer solution removed residual lignin, yielding delignified wood (DW).





**Fig. S3.** FTIR spectra of NW, DW and PHW.

The removal of lignin was evidenced by the disappearance of characteristic peaks for aromatic C=C stretching at 1596 cm^-1^ and C–H bending at 1453 cm^-1^. Furthermore, a new –COOH absorption peak (1599 cm^-1^) in PHW indicated successful carboxymethylation.





**Fig. S4.** High-resolution C 1s spectra of NW and PHW.

Through peak fitting, the NW showed three chemical states of carbon: C-C (bond energy 284.8 eV), C-O (286.2 eV), and O-C-O (288.2 eV). In addition to C-C and C-O, the PHW sample showed a characteristic peak at O-C=O (289.4 eV), indicating the introduction of additional carboxyl groups in the PHW.





**Fig. S5.** Contents of the three major components in NW, DESW, and DW.

During the chemical treatment process, lignin and hemicellulose were gradually removed, resulting in a significant increase in cellulose content.





**Fig. S6.** Carboxyl content of NW and CW.


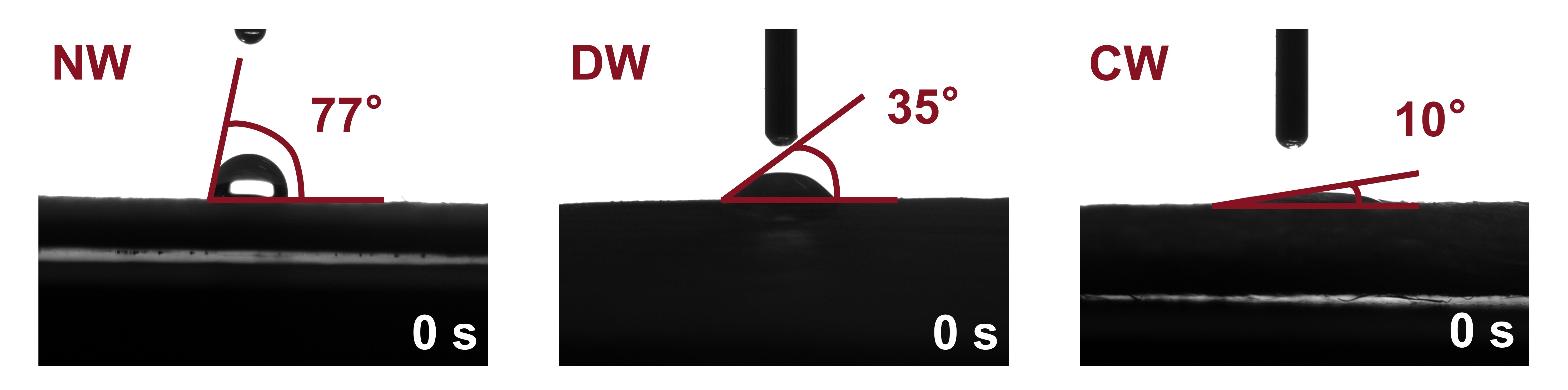


**Fig. S7.** Dynamic water contact angle of NW, DW and CW.

The contact angle decreased from 77° for NW to 35° for DW and further to 10° for CW, confirming that lignin removal and carboxyl group introduction substantially improved the surface hydrophilicity.


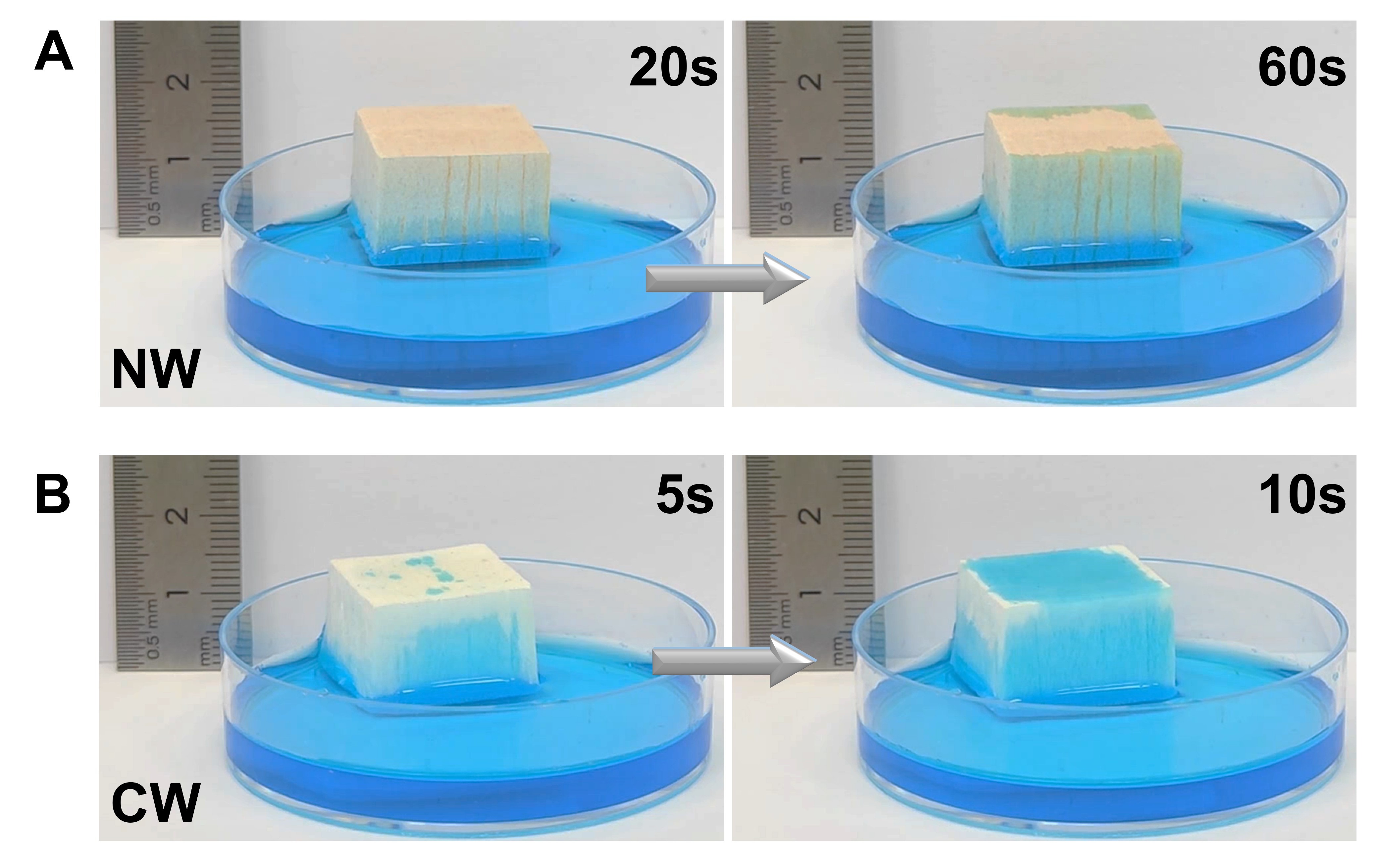


**Fig. S8.** Dye transport experiments of NW and CW.

The introduction of demethylation and carboxylation enhanced water transport capacity compared to natural wood.





**Fig. S9.** XRD patterns of NW, CW, LiCl, and PHW.


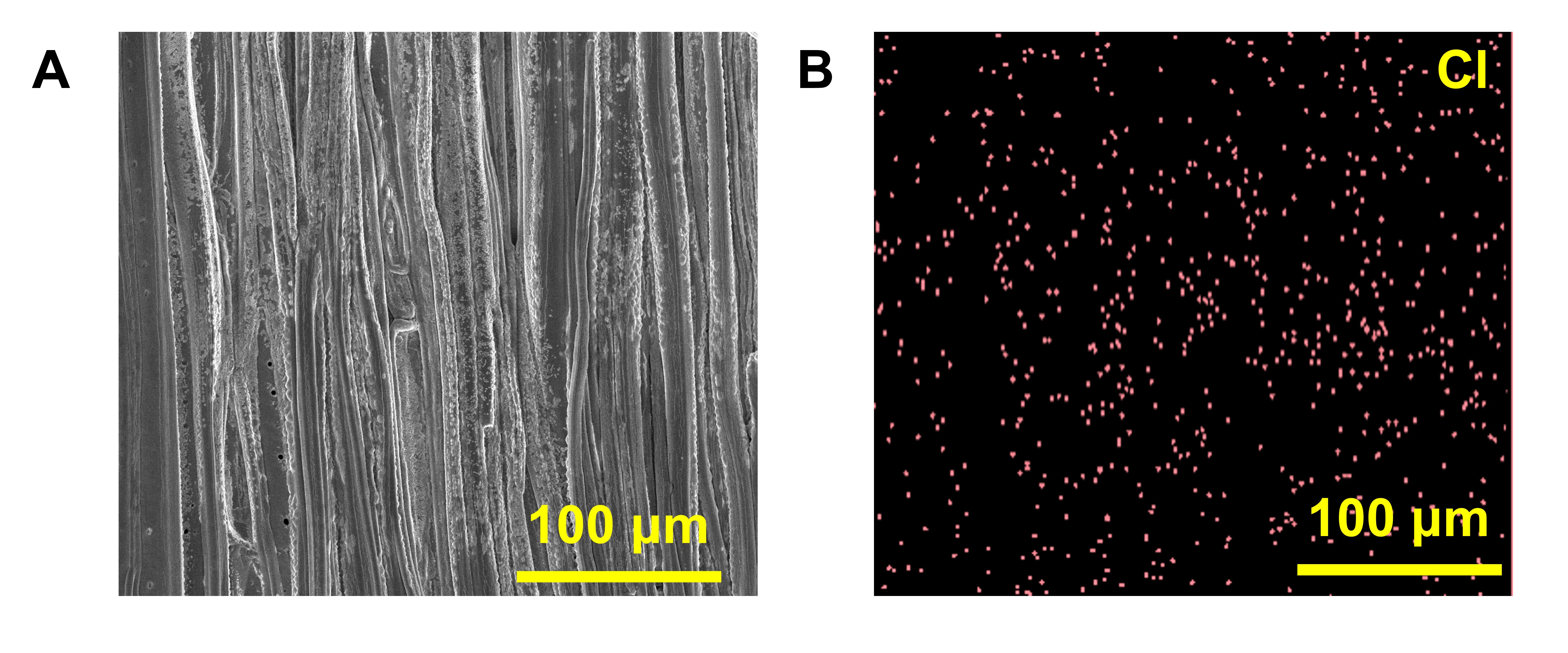


**Fig. S10.** (A) SEM image and (B) EDS mapping of PHW after LiCl loading.

SEM images confirmed that the aligned wood microchannels remained intact after LiCl incorporation (Fig. S10A). EDS mapping of Cl further verified the presence of LiCl within the scaffold (Fig. S10B).


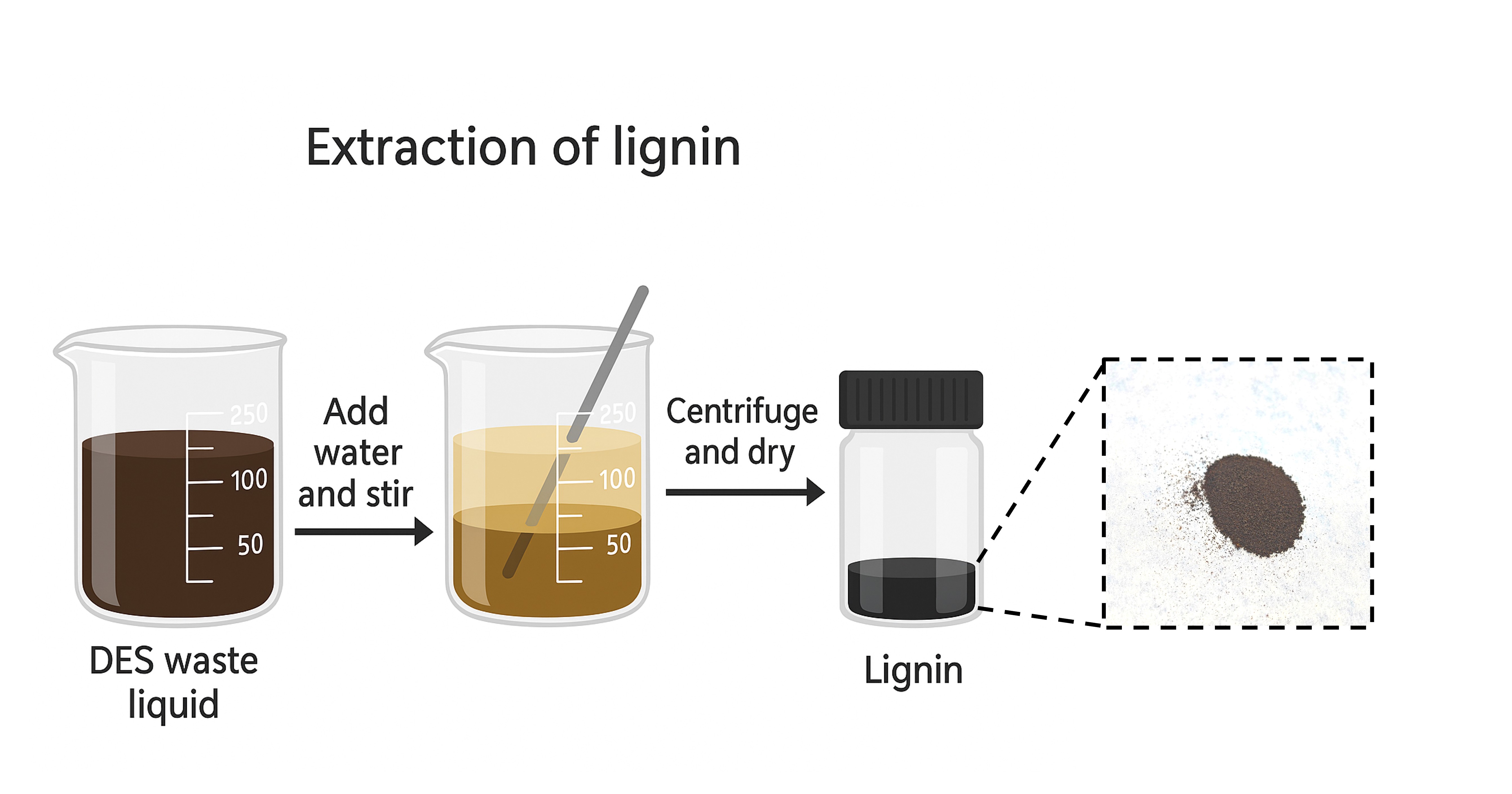


**Fig. S11.** Lignin extraction process: utilizing the anti-solvent method from DES waste liquid.





**Fig. S12.** Phenolic hydroxyl content of lignin before and after demethylation.





**Fig. S13.** ^1^H NMR spectra of lignin before and after demethylation (D-lignin).

Compared with pristine lignin, D-lignin shows a significantly attenuated methoxy (OMe, –OCH_3_) resonance (~3.6–3.9 ppm), indicating effective demethylation and a concurrent increase in phenolic hydroxyl groups.


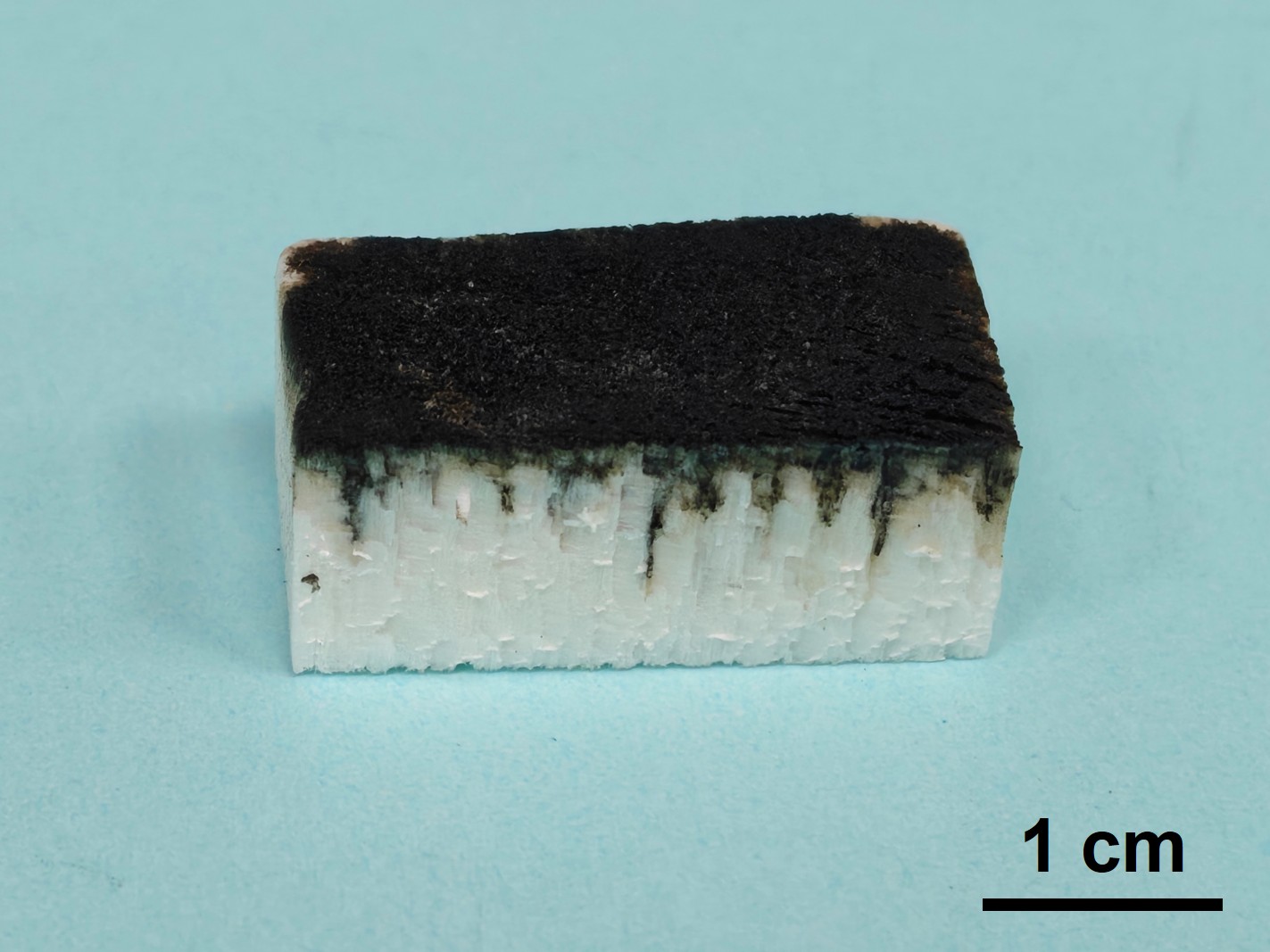


**Fig. S14.** Sectional diagram of PHW.





**Fig. S15.** XPS survey spectra of NB and PHW.





**Fig. S16.** Pore size distribution curves of NW and PHW.





**Fig. S17.** Water absorption rate of PHW impregnated with LiCl solutions of varying concentrations over time.





**Fig. S18.** LiCl Content in PHW Immersed in Different LiCl Solutions.


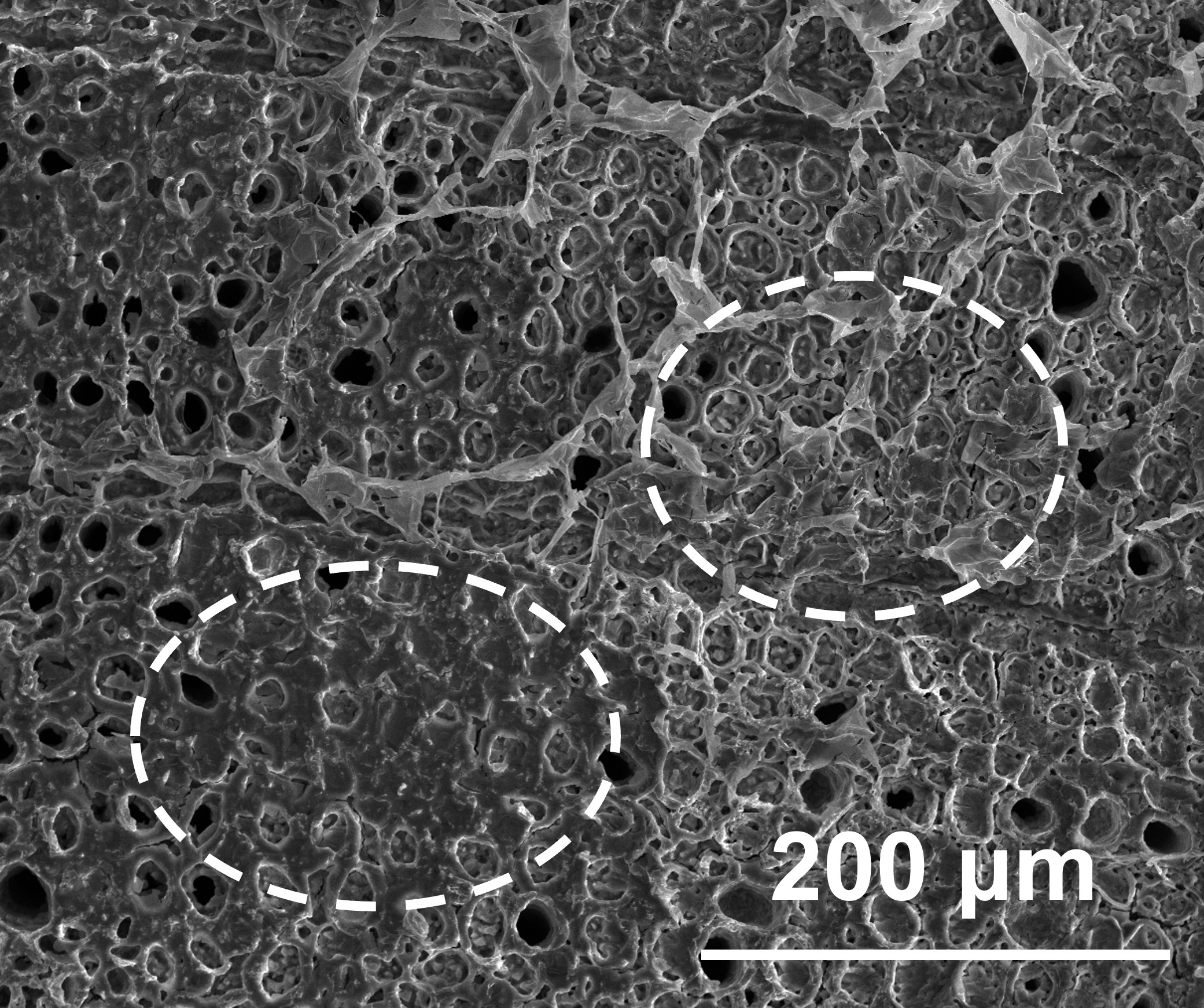


**Fig. S19.** Cross-section of PHW impregnated with 20wt% LiCl solution. The circle highlighted the pore channels blocked by LiCl.


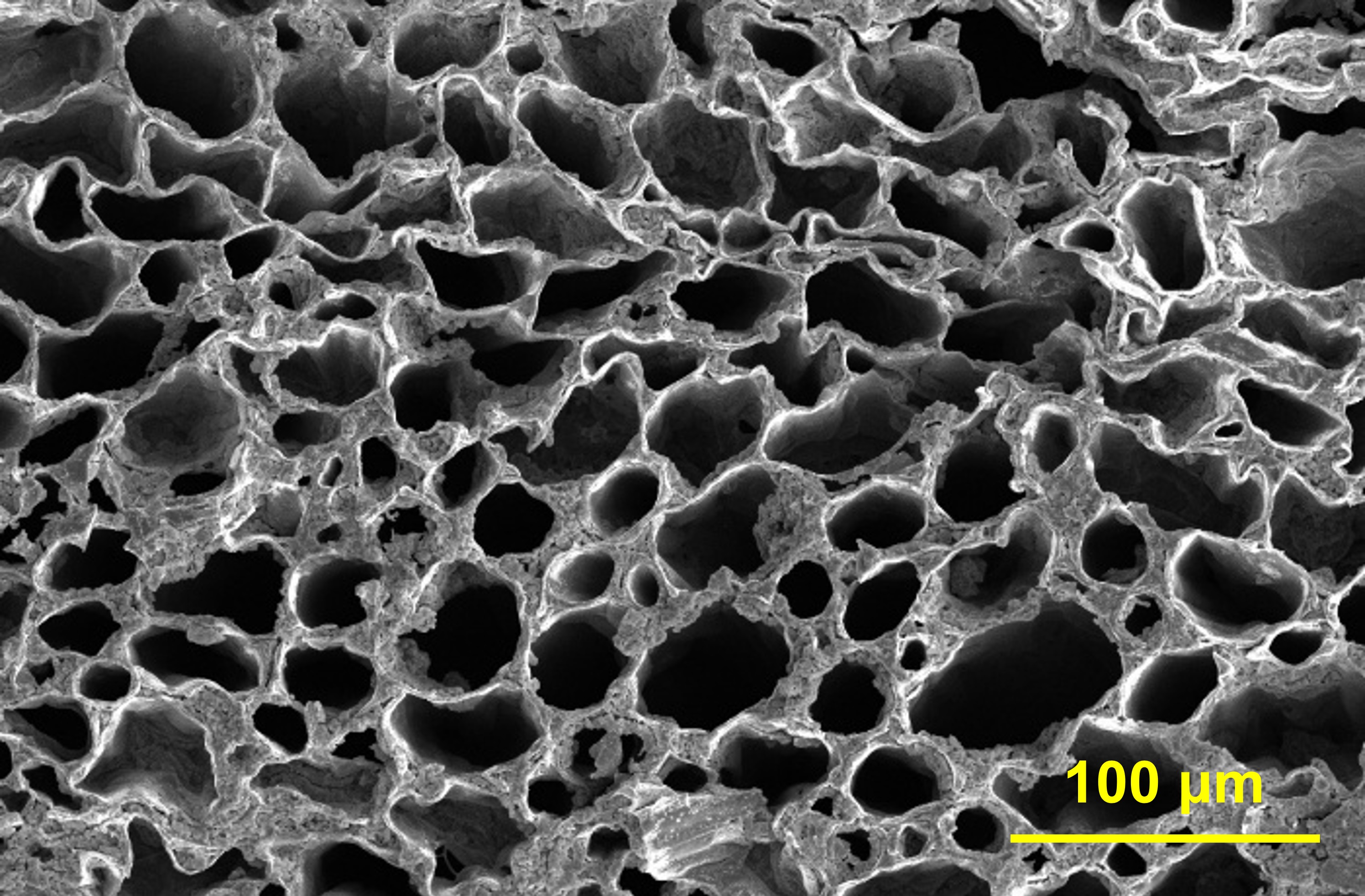


**Fig. S20.** Cross-sectional SEM image of the hygroscopic layer in PHW after delignification/carboxylation and LiCl incorporation, showing a retained open, interconnected pore network with vapor-accessible microchannels.


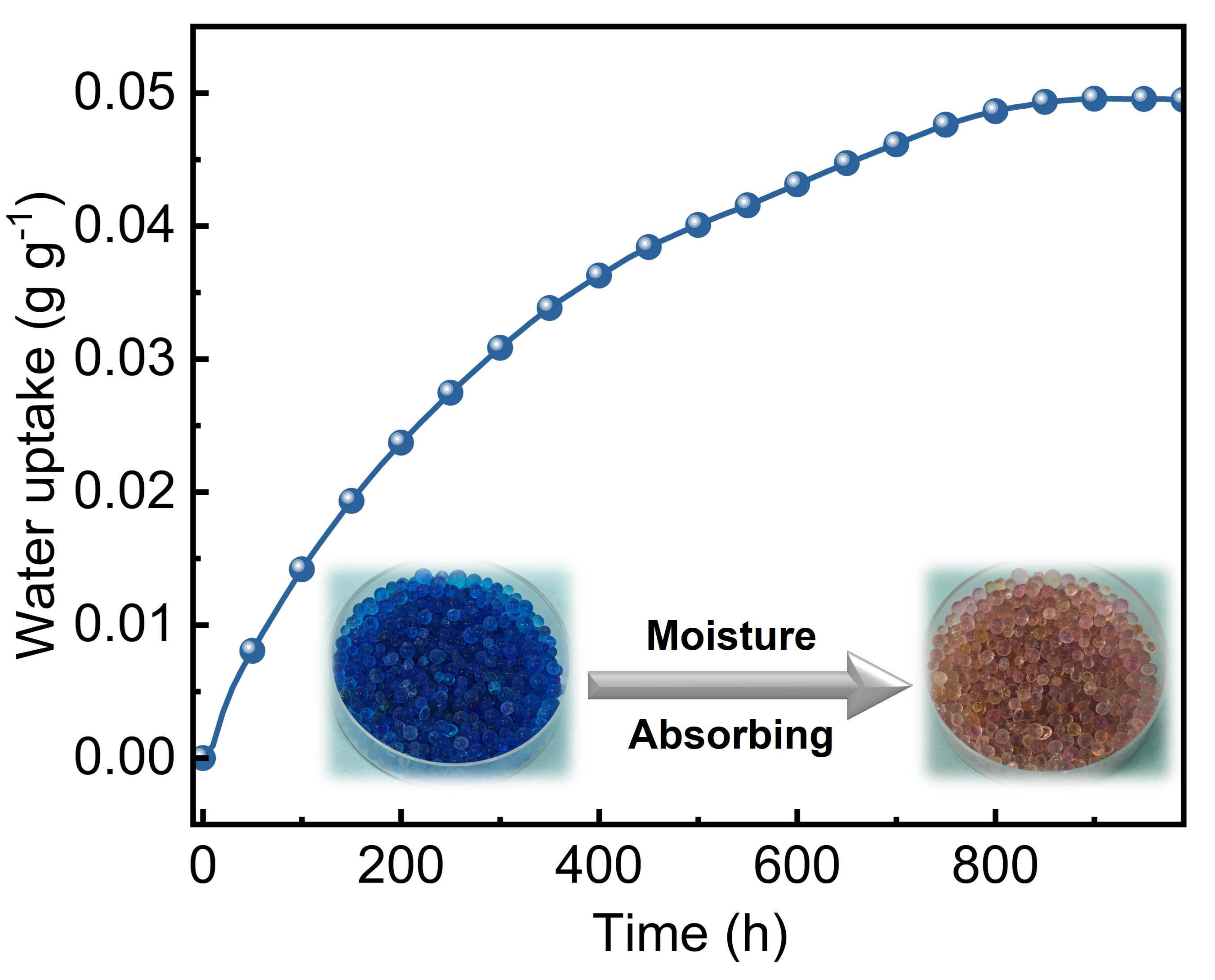


**Fig. S21.** Moisture absorption curve of silicone rubber (RH=70%), along with actual images before and after moisture absorption.


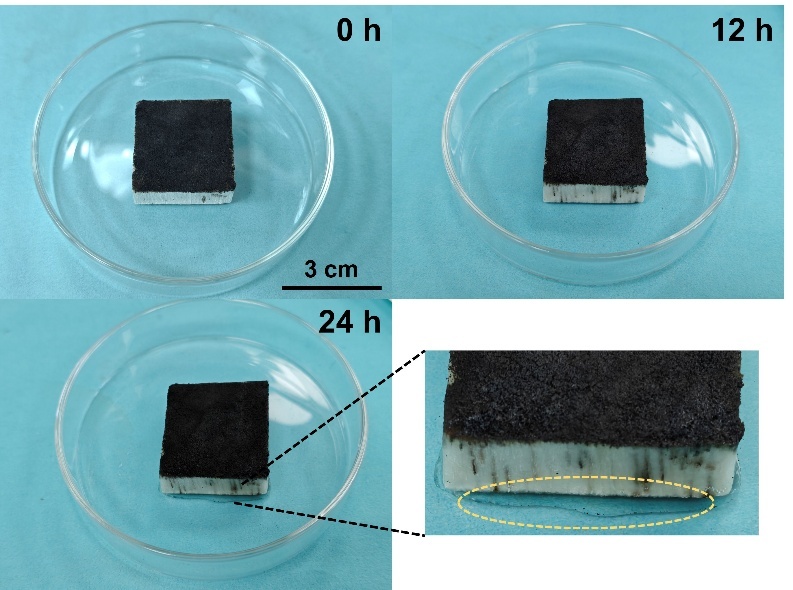


**Fig. S22.** Physical image of PHW after prolonged moisture absorption.





**Fig. S23.** Moisture absorption curve of PHW over an extended period.





**Fig. S24.** Moisture uptake kinetics of the LiCl-free PHW control measured under identical conditions. The control used the same delignified–carboxylated wood scaffold and the same top photothermal coating, but without LiCl impregnation.





**Fig. S25.** Diffusion fitting of water sorption data based on Fick’s second law of PHW under various *RH* conditions (30% to 90% *RH*, 25 °C).

**
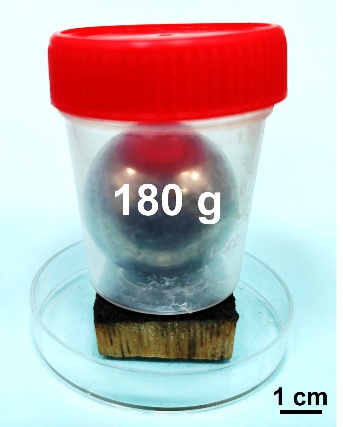
**

**Fig. S26.** Photographs of PHW after repeated sorption–desorption cycling and load-bearing demonstration. The cycled PHW maintained its macroscopic integrity and was able to support an external weight without visible cracking or collapse, indicating retained structural robustness after multiple adsorption–desorption cycles.


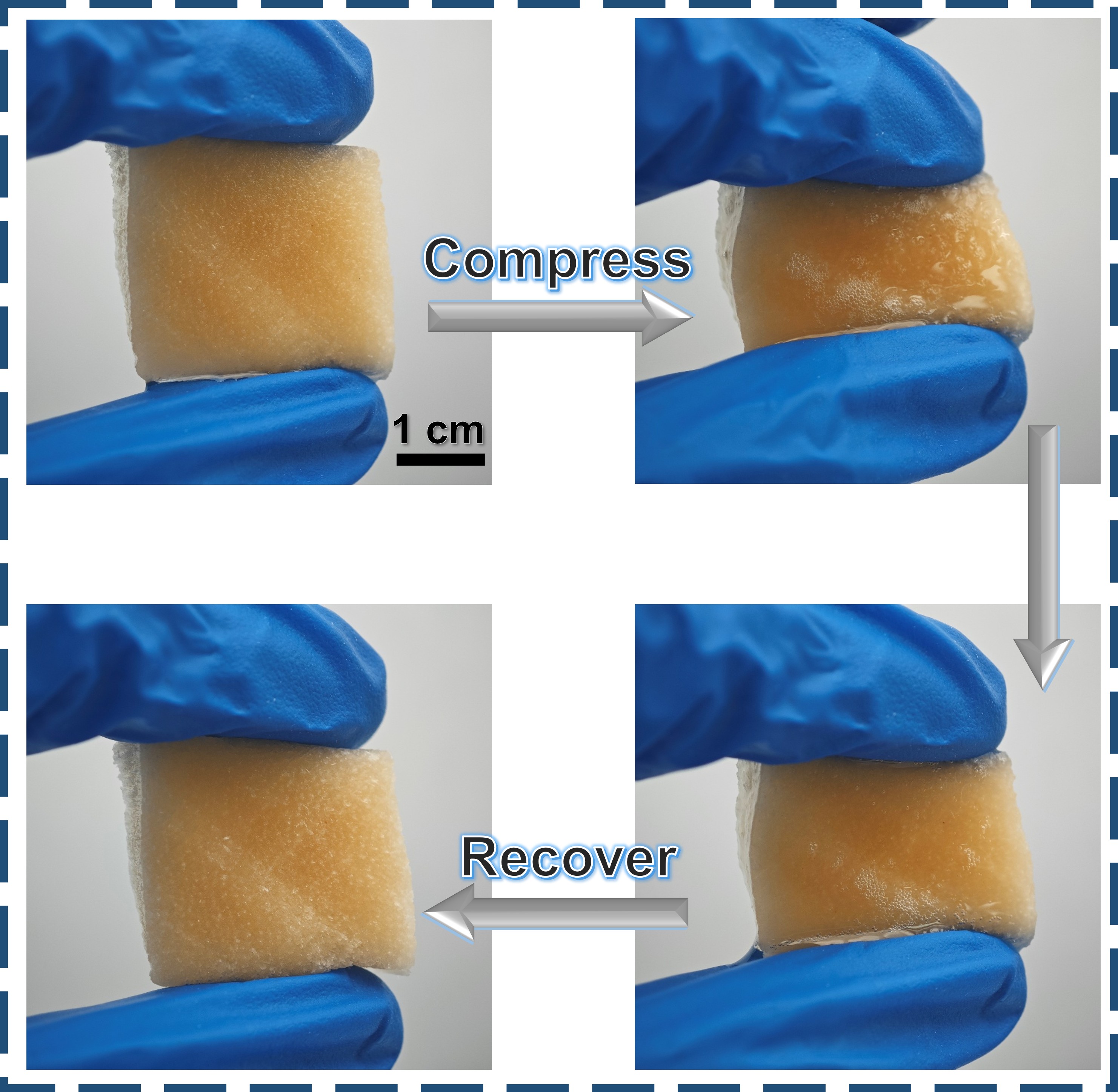


**Fig. S27.** Compression and recovery performance demonstration of moisture-absorbing wood sponge.


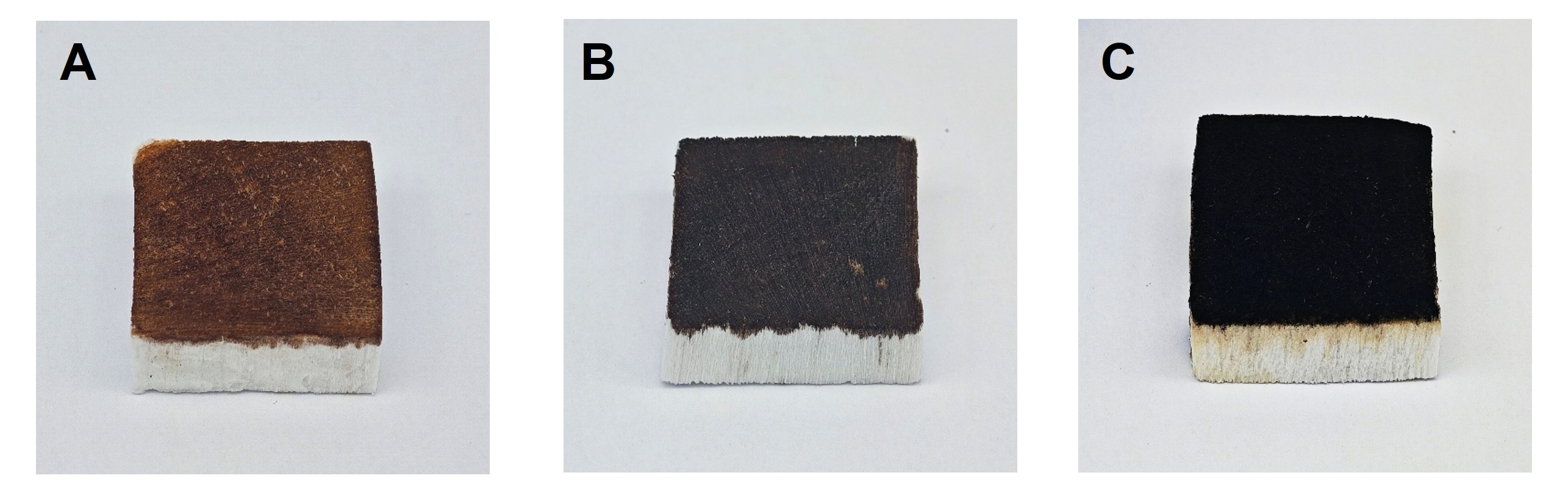


**Fig. S28.** PHW loaded with different types of lignin-based photothermal coatings: (A) Lignin, (B) D-Lignin, and (C) D-Lignin-Fe.





**Fig. S29.** Desorption rate curves for D-Lignin-Fe coatings with varying loading amounts (10-90mg).


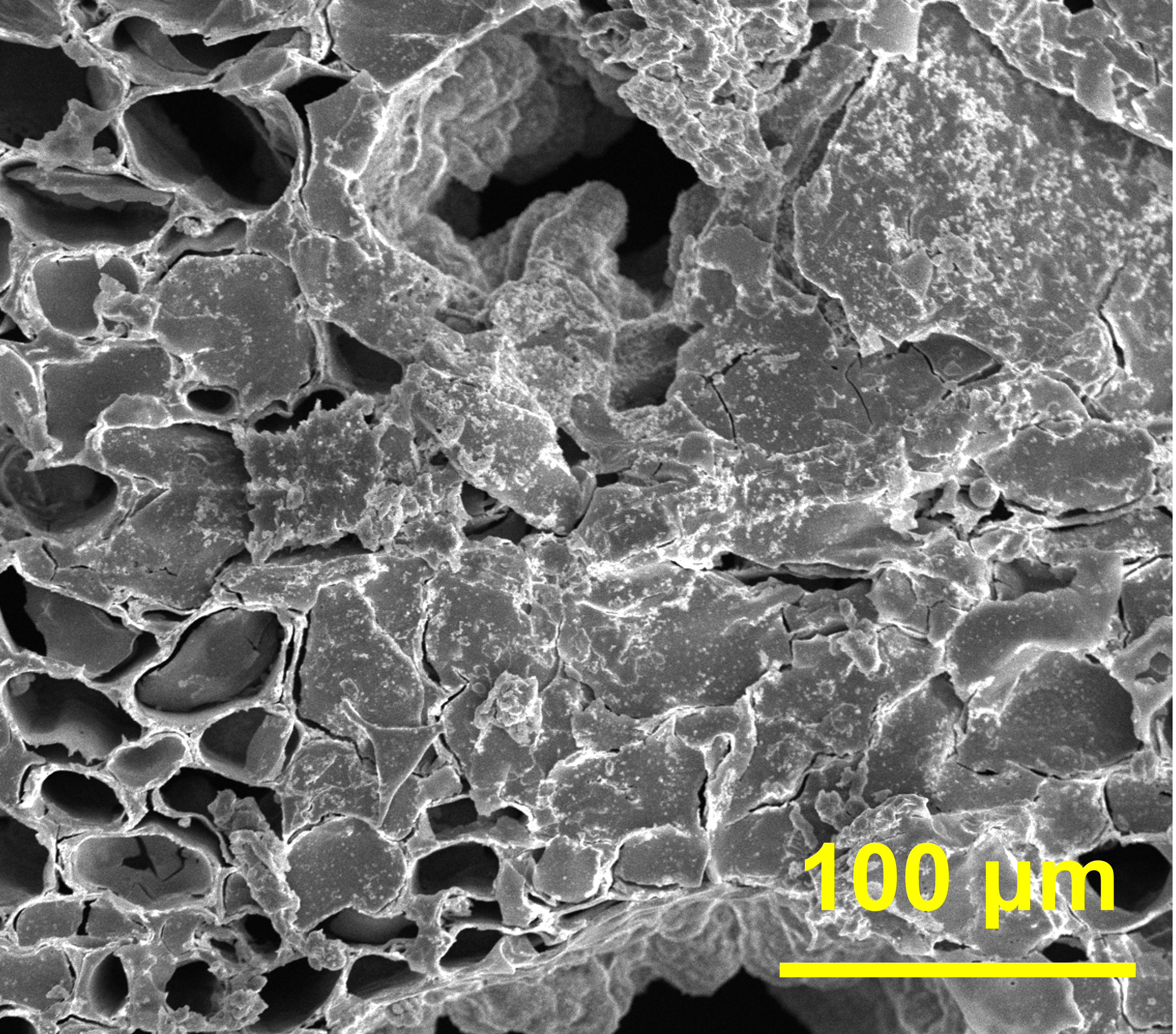


**Fig. S30.** Evaporative surface of PHW with a 90 mg loading of D-Lignin-Fe.





**Fig. S31.** Zeta potential of the upper evaporation layer and lower hygroscopic layer in PHW.





**Fig. S32.** Voltage output curve of the PHW without LiCl.





**Fig. S33.** *I_sc_* of PHW-HG under different relative humidities (30%, 60%, and 90% *RH*).





**Fig. S34.** Voltage output over repeated moisture-absorption cycles.





**Fig. S35.** *I_sc_* of PHW-HG under different light intensities (0.5–2 suns).





**Fig. S36.** Voltage output under prolonged simulated light desorption (1 sun).


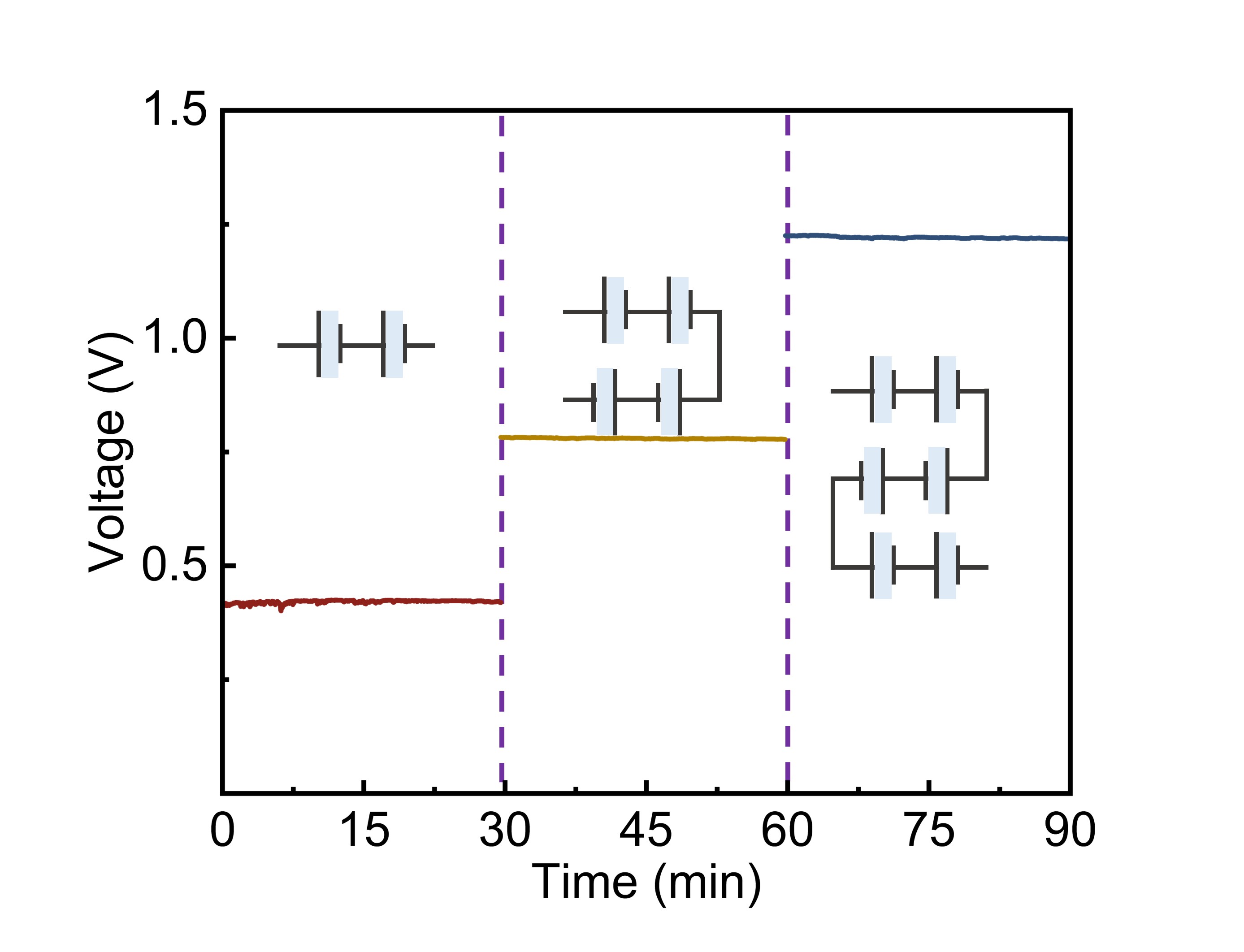


**Fig. S37.** Voltage output of HG modules connected in series under 60% *RH.*


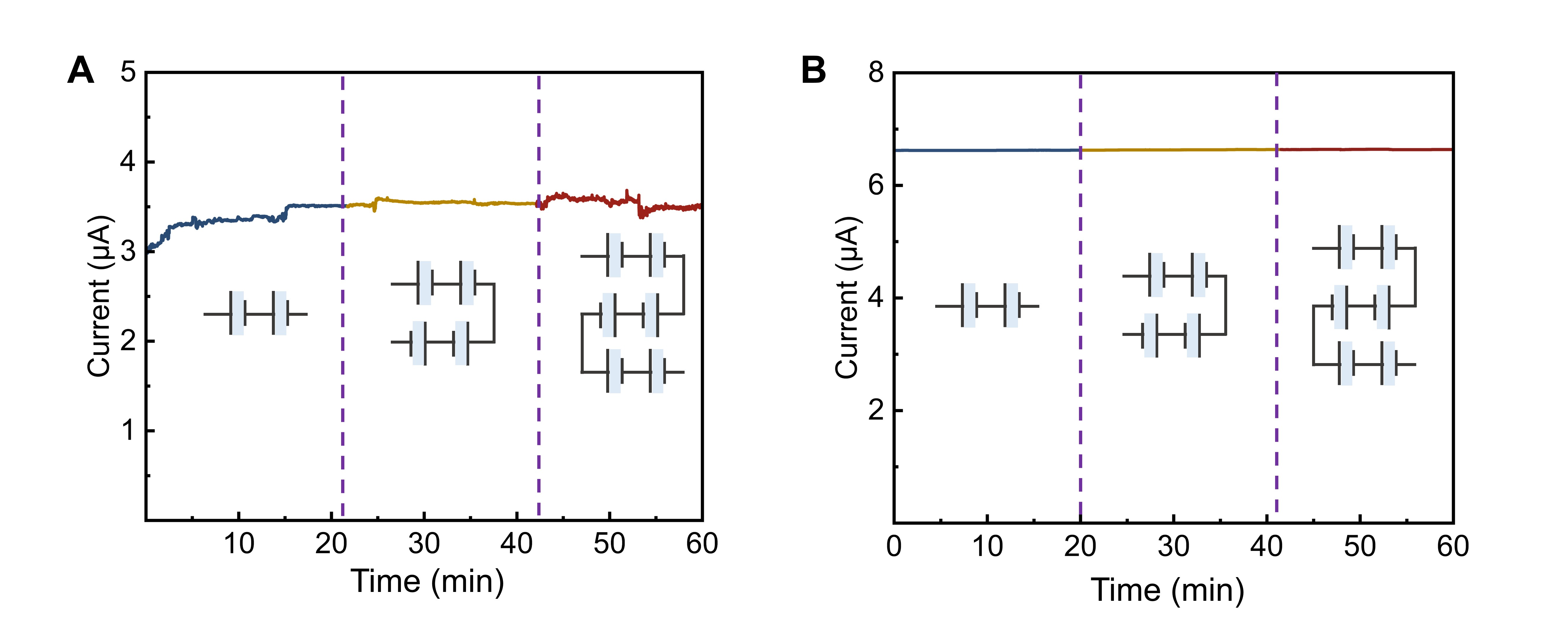


**Fig. S38.** *I_sc_* of PHW-HG modules connected in series under (a) 60% *RH* and (b) 1 sun.

**
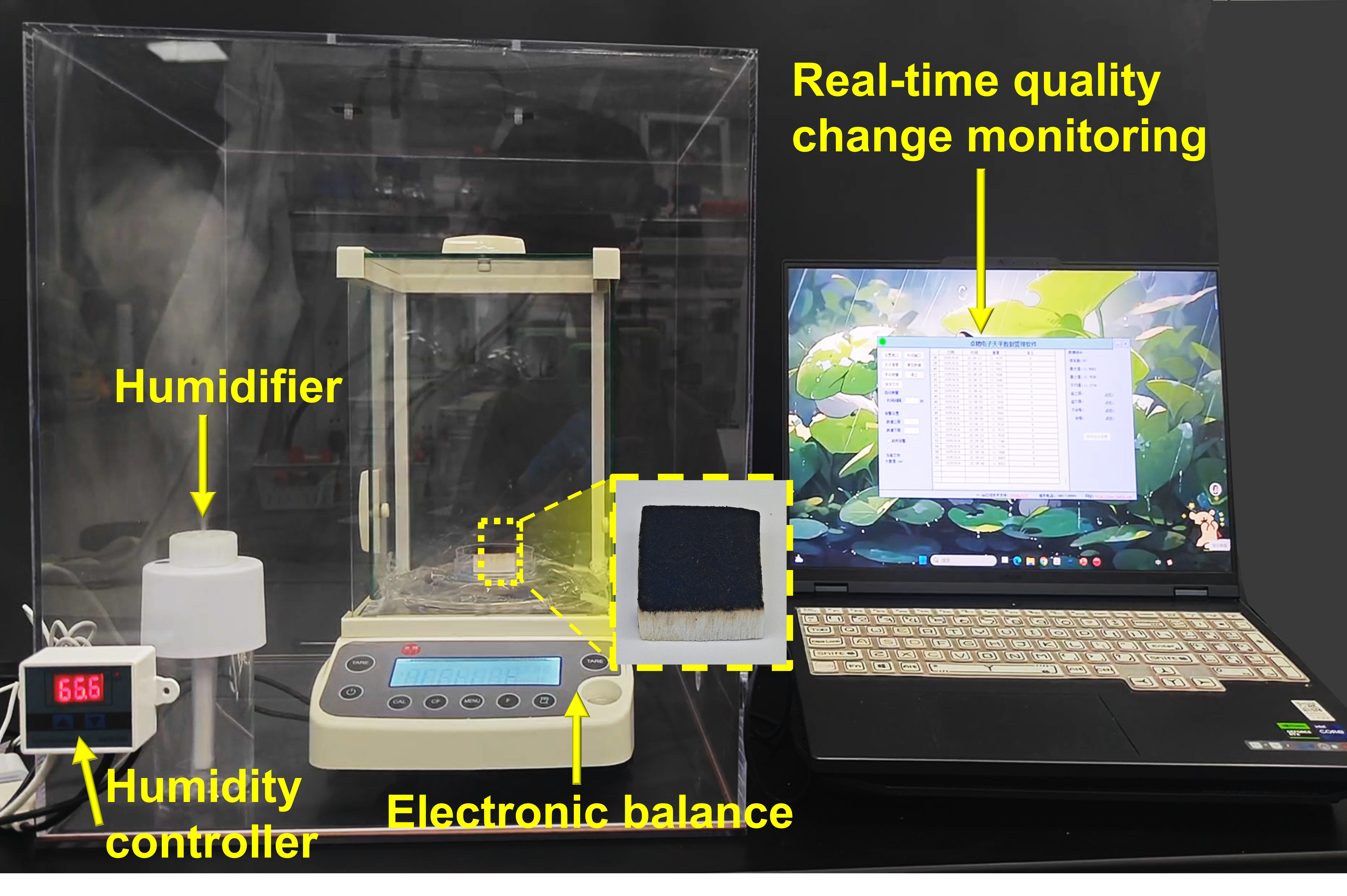
**

**Fig. S39.** Custom-built apparatus used to evaluate the hygroscopic performance of the samples.

Humidity-controlled sorption test (Fig. S39). The sorption experiment was performed in a sealed environmental chamber (internal dimensions: 0.4 × 0.4 × 0.4 m). A programmable humidity generator was used for *RH* control. The setpoint was maintained by a closed-loop feedback scheme in which an integrated humidity sensor and humidification unit were coupled to a controller. Owing to the small chamber volume, rapid moisture-field equilibration was expected under steady-state operation, and no macroscopic humidity stratification was observed during the tests. The experiment was designed to operate at fixed *RH* setpoints rather than to resolve transient RH fluctuations; continuous *RH* logging inside the chamber was therefore not implemented in the original measurements. This operating boundary has been stated here for clarity and reproducibility.

**Supplementary Tables**

**Table S1.** The contents of the three major components (cellulose, hemicellulose and lignin) in NW, DESW, and DW.

| **Sample** | **Cellulose (%)** | **Hemicellulose (%)** | **Lignin (%)** | |
| --- | --- | --- | --- | --- |
| NW | 42.42±2.01 | 40.72±1.99 | | 14.74±1.66 |
| DESW | 67.43±2.65 | 23.78±1.54 | | 8.48±1.08 |
| DW | 89.78±2.75 | 6.78±1.06 | | 1.39±0.65 |

**Table S2.** The carboxyl content of NW and PHW.

| **Sample** | **Carboxyl content (mmol g^-1^)** |
| --- | --- |
| NW | 0.026±0.007 |
| PHW | 1.359±0.132 |

**Table S3.** The specific surface area and pore size parameters of NW and PHW.

| **Sample** |  | **BET surface area (m^2^ g^-1^)** | **Adsorption average pore width (nm)** |
| --- | --- | --- | --- |
| NW |  | 3.63 | 5.46 |
| PHW |  | 4.16 | 4.70 |

**Table S4.** The linear fitting for the diffusion coefficient under different concentrations of LiCl (wt%*)* based on Fick’s second law.

| **Concentrations of LiCl (wt%)** | **Water uptake (g g^-1^)** | **Fitting equation** | **R^2^** |
| --- | --- | --- | --- |
| 0 | 0.3117 | y=0.0303x-0.1572 | 0.9925 |
| 5 | 1.1055 | y=0.0332x-0.1725 | 0.9963 |
| 10 | 1.4763 | y=0.0421x-0.1887 | 0.9983 |
| 15 | 1.8045 | y=0.0704x-0.2625 | 0.9874 |
| 20 | 1.396 | y=0.0414x-0.1947 | 0.9977 |
| 25 | 1.2618 | y=0.0358x-0.1919 | 0.9947 |
| 30 | 1.0300 | y=0.0312x-0.1493 | 0.9958 |

**Table S5.** The hygroscopic performance of different samples was conducted under 70% *RH* at 25 °C.

| **Sample** | **Water uptake (g g^-1^)** |
| --- | --- |
| NW | 0.0948 |
| CW | 0.3130 |
| HW | 1.8973 |
| PHW | 1.8932 |

**Table S6.** The linear fitting for the diffusion coefficient under different *RH* based on Fick’s second law.

| ***RH*** | **Water uptake (g g^-1^)** | **Fitting equation** | **R^2^** |
| --- | --- | --- | --- |
| 30 | 0.7531 | y=0.03242x-0.15184 | 0.97138 |
| 40 | 1.0137 | y=0.03608x-0.21857 | 0.97318 |
| 50 | 1.1882 | y=0.03693x-0.19097 | 0.96994 |
| 60 | 1.4711 | y=0.05112x-0.28501 | 0.99621 |
| 70 | 1.8627 | y=0.06085x-0.28348 | 0.99802 |
| 80 | 2.3308 | y=0.07702x-0.22596 | 0.99148 |
| 90 | 2.5166 | y=0.08053x-0.42674 | 0.98843 |

**Table S7.** Comparison of water uptake capacities (g g^-1^) of PHW with previously reported state-of-the-art atmospheric water harvesters under varying *RH* conditions.

| **Materials** | **30% *RH*** | **40% *RH*** | **50% *RH*** | **60% *RH*** | **70% *RH*** | **80% *RH*** | **90% *RH*** | **Ref** |
| --- | --- | --- | --- | --- | --- | --- | --- | --- |
| **PHW-AWHG** | **0.75** | **1.01** | **1.18** | **1.47** | **1.86** | **2.33** | **2.51** | **This work** |
| Cellulose-based sponges | 0.61 | - | 1.08 | - | 1.51 | - | 2.25 | [48] |
| CNT/LiCl/MOF (801) | - | 0.71 | - | - | - | 1.95 | - | [49] |
| Perovskite/LiCl/cellulose aerogels | 0.55 | 0.61 | 0.72 | 0.78 | 1.18 | 1.31 | 1.45 | [50] |
| 3D cellulose aerogels | 0.55 | - | - | 1.34 | - | 1.68 | 1.78 | [46] |
| PPy/MOF (801)/CA aerogel | - | 0.677 | - | 1.15 | - | - | - | [29] |
| Carbonized wood/ZnCl_2_ | - | 0.15 | 0.26 | 0.34 | 0.53 | 0.59 | - | [51] |
| SA/AF/EG/LiCl | 0.5 |  |  | 1.18 |  |  |  | [52] |
| PAM/CaCl_2_ | 0.6 |  |  | 1.2 |  |  | 2.1 | [53] |
| GO/SA/GA/LiCl | 0.73 |  | 1.64 |  | 2.34 |  |  | [54] |
| CMCS/PVP/PPY/LiCl/CaCl_2_ |  | 1.6 |  | 2.2 |  | 3.9 | 5.7 | [18] |
| BC/SA/GO-LiCl | 1.35 |  |  | 2.15 |  |  | 4.12 | [55] |
| HPC/LiCl/CaCl_2_ | 1.25 |  |  | 2.25 |  |  |  | [56] |
| MPTC/NaSS/LiCl | 1.15 |  | 2.2 |  | 3.15 |  | 5.2 | [57] |
| SA/SiO_2_/BN/LiCl/CaCl_2_ | 0.5 | - | 0.81 | - | 1.25 | - | - | [58] |
| HPMC/PAAS/LiCl | 0.9 | - | 1.89 | 1.18 | - | - | 4.3 | [59] |

**Table S8.** Ion concentration of harvested water compared with WHO drinking water standards (mg l^-1^).

| **Sample** | **Harvested water** | **WHO drinking water standards** |
| --- | --- | --- |
| Li^+^ | 0.0208 | N/A |
| Fe^3+^ | 0.0214 | N/A |
| Mg^2+^ | 0.0736 | N/A |
| Na^+^ | 1.7218 | N/A |
| Ca^2+^ | 1.1630 | N/A |
| F^-^ | 0.1000 | 1.5 |
| NO_2_^-^ | 0.4410 | 3 |
| NO_3_^-^ | 2.2770 | 50 |
| Cr | 0.0261 | 0.05 |

**Table S9.** Comparison of environmental impact characteristics between PHW and silica gel across various metrics.

| **Impact category** | **Unit** | **Silica gel** | **PHW** |
| --- | --- | --- | --- |
| Global warming | kg CO_2_ eq. per kg | 2.44 | 1.11 |
| Fossil resource scarcity | USD2013 | 0.222 | 0.0455 |
| Mineral resource scarcity | USD2013 | 0.00133 | 9.14E-5 |
| Marine ecotoxicity | species.yr | 5.81E-11 | 3.07E-12 |
| Terrestrial ecotoxicity | species.yr | 5.81E-11 | 3.07E-12 |
| Ozone formation | DALY | 7.4E-10 | 5.58E-11 |
| Freshwater ecotoxicity | species.yr | 6.44E-11 | 3.44E-12 |
| Water consumption | species.yr | 6.52E-14 | 2.27E-14 |
