# Supplementary material for: Biomimetic All-Wood Sponge for the Co-Generation of Adsorption-Based Atmospheric Water Harvesting and Hydrovoltaic Power Generation
Source: Research (Wash D C). 2026 Mar 24;9:1195. doi: 10.34133/research.1195 (PMC13009533; doi:10.34133/research.1195)
Supplement: Supplementary 1 — Figs. S1 to S39 Tables S1 to S9 Movies S1 to S3 [file research.1195.f1.zip › Supplementary Tables.docx]

**Supplementary Tables**

**Table S1.** The contents of the three major components (cellulose, hemicellulose and lignin) in NW, DESW, and DW.

| **Sample** | **Cellulose (%)** | **Hemicellulose (%)** | **Lignin (%)** | |
| --- | --- | --- | --- | --- |
| NW | 42.42±2.01 | 40.72±1.99 | | 14.74±1.66 |
| DESW | 67.43±2.65 | 23.78±1.54 | | 8.48±1.08 |
| DW | 89.78±2.75 | 6.78±1.06 | | 1.39±0.65 |

**Table S2.** The carboxyl content of NW and PHW.

| **Sample** | **Carboxyl content (mmol g^-1^)** |
| --- | --- |
| NW | 0.026±0.007 |
| PHW | 1.359±0.132 |

**Table S3.** The specific surface area and pore size parameters of NW and PHW.

| **Sample** |  | **BET surface area (m^2^ g^-1^)** | **Adsorption average pore width (nm)** |
| --- | --- | --- | --- |
| NW |  | 3.63 | 5.46 |
| PHW |  | 4.16 | 4.70 |

**Table S4.** The linear fitting for the diffusion coefficient under different concentrations of LiCl (wt%*)* based on Fick’s second law.

| **Concentrations of LiCl (wt%)** | **Water uptake (g g^-1^)** | **Fitting equation** | **R^2^** |
| --- | --- | --- | --- |
| 0 | 0.3117 | y=0.0303x-0.1572 | 0.9925 |
| 5 | 1.1055 | y=0.0332x-0.1725 | 0.9963 |
| 10 | 1.4763 | y=0.0421x-0.1887 | 0.9983 |
| 15 | 1.8045 | y=0.0704x-0.2625 | 0.9874 |
| 20 | 1.396 | y=0.0414x-0.1947 | 0.9977 |
| 25 | 1.2618 | y=0.0358x-0.1919 | 0.9947 |
| 30 | 1.0300 | y=0.0312x-0.1493 | 0.9958 |

**Table S5.** The hygroscopic performance of different samples conducted under 70% *RH* at 25 °C.

| **Sample** | **Water uptake (g g^-1^)** |
| --- | --- |
| NW | 0.0948 |
| CW | 0.3130 |
| HW | 1.8973 |
| PHW | 1.8932 |

**Table S6.** The linear fitting for diffusion coefficient under different *RH* based on Fick’s second law.

| ***RH*** | **Water uptake (g g^-1^)** | **Fitting equation** | **R^2^** |
| --- | --- | --- | --- |
| 30 | 0.7531 | y=0.03242x-0.15184 | 0.97138 |
| 40 | 1.0137 | y=0.03608x-0.21857 | 0.97318 |
| 50 | 1.1882 | y=0.03693x-0.19097 | 0.96994 |
| 60 | 1.4711 | y=0.05112x-0.28501 | 0.99621 |
| 70 | 1.8627 | y=0.06085x-0.28348 | 0.99802 |
| 80 | 2.3308 | y=0.07702x-0.22596 | 0.99148 |
| 90 | 2.5166 | y=0.08053x-0.42674 | 0.98843 |

**Table S7.** Comparison of water uptake capacities (g g^-1^) of PHW with previously reported state-of-the-art atmospheric water harvesters under varying *RH* conditions.

| **Materials** | **30% *RH*** | **40% *RH*** | **50% *RH*** | **60% *RH*** | **70% *RH*** | **80% *RH*** | **90% *RH*** | **Ref** |
| --- | --- | --- | --- | --- | --- | --- | --- | --- |
| **PHW-AWHG** | **0.75** | **1.01** | **1.18** | **1.47** | **1.86** | **2.33** | **2.51** | **This work** |
| Cellulose-based sponges | 0.61 | - | 1.08 | - | 1.51 | - | 2.25 | [49] |
| CNT/LiCl/MOF (801) | - | 0.71 | - | - | - | 1.95 | - | [50] |
| Perovskite/LiCl/cellulose aerogels | 0.55 | 0.61 | 0.72 | 0.78 | 1.18 | 1.31 | 1.45 | [51] |
| 3D cellulose aerogels | 0.55 | - | - | 1.34 | - | 1.68 | 1.78 | [52] |
| PPy/MOF (801)/CA aerogel | - | 0.677 | - | 1.15 | - | - | - | [53] |
| Carbonized wood/ZnCl_2_ | - | 0.15 | 0.26 | 0.34 | 0.53 | 0.59 | - | [54] |
| SA/AF/EG/LiCl | 0.5 |  |  | 1.18 |  |  |  | [55] |
| PAM/CaCl_2_ | 0.6 |  |  | 1.2 |  |  | 2.1 | [56] |
| GO/SA/GA/LiCl | 0.73 |  | 1.64 |  | 2.34 |  |  | [57] |
| CMCS/PVP/PPY/LiCl/CaCl_2_ |  | 1.6 |  | 2.2 |  | 3.9 | 5.7 | [18] |
| BC/SA/GO-LiCl | 1.35 |  |  | 2.15 |  |  | 4.12 | [58] |
| HPC/LiCl/CaCl_2_ | 1.25 |  |  | 2.25 |  |  |  | [59] |
| MPTC/NaSS/LiCl | 1.15 |  | 2.2 |  | 3.15 |  | 5.2 | [60] |
| SA/SiO_2_/BN/LiCl/CaCl_2_ | 0.5 | - | 0.81 | - | 1.25 | - | - | [61] |
| HPMC/PAAS/LiCl | 0.9 | - | 1.89 | 1.18 | - | - | 4.3 | [62] |

**Table S8.** Ion concentration of harvested water compared with WHO drinking water standards (mg l^-1^).

| **Sample** | **Harvested water** | **WHO drinking water standards** |
| --- | --- | --- |
| Li^+^ | 0.0208 | N/A |
| Fe^3+^ | 0.0214 | 0.3 |
| Mg^2+^ | 0.0736 | 10 |
| Na^+^ | 1.7218 | 20 |
| Ca^2+^ | 1.1630 | 20 |

**Table S9.** Comparison of environmental impact characteristics between PHW and silicone across various metrics.

| **Impact category** | **Unit** | **Silicone** | **PHW** |
| --- | --- | --- | --- |
| Global warming | kg CO_2_ eq. per kg | 2.44 | 1.11 |
| Fossil resource scarcity | USD2013 | 0.222 | 0.0455 |
| Mineral resource scarcity | USD2013 | 0.00133 | 9.14E-5 |
| Marine ecotoxicity | species.yr | 5.81E-11 | 3.07E-12 |
| Terrestrial ecotoxicity | species.yr | 5.81E-11 | 3.07E-12 |
| Ozone formation | DALY | 7.4E-10 | 5.58E-11 |
| Freshwater ecotoxicity | species.yr | 6.44E-11 | 3.44E-12 |
| Water consumption | species.yr | 6.52E-14 | 2.27E-14 |
