# Supplementary figures and images for: Biomimetic All-Wood Sponge for the Co-Generation of Adsorption-Based Atmospheric Water Harvesting and Hydrovoltaic Power Generation
Source: Research (Wash D C). 2026 Mar 24;9:1195. doi: 10.34133/research.1195 (PMC13009533; doi:10.34133/research.1195)

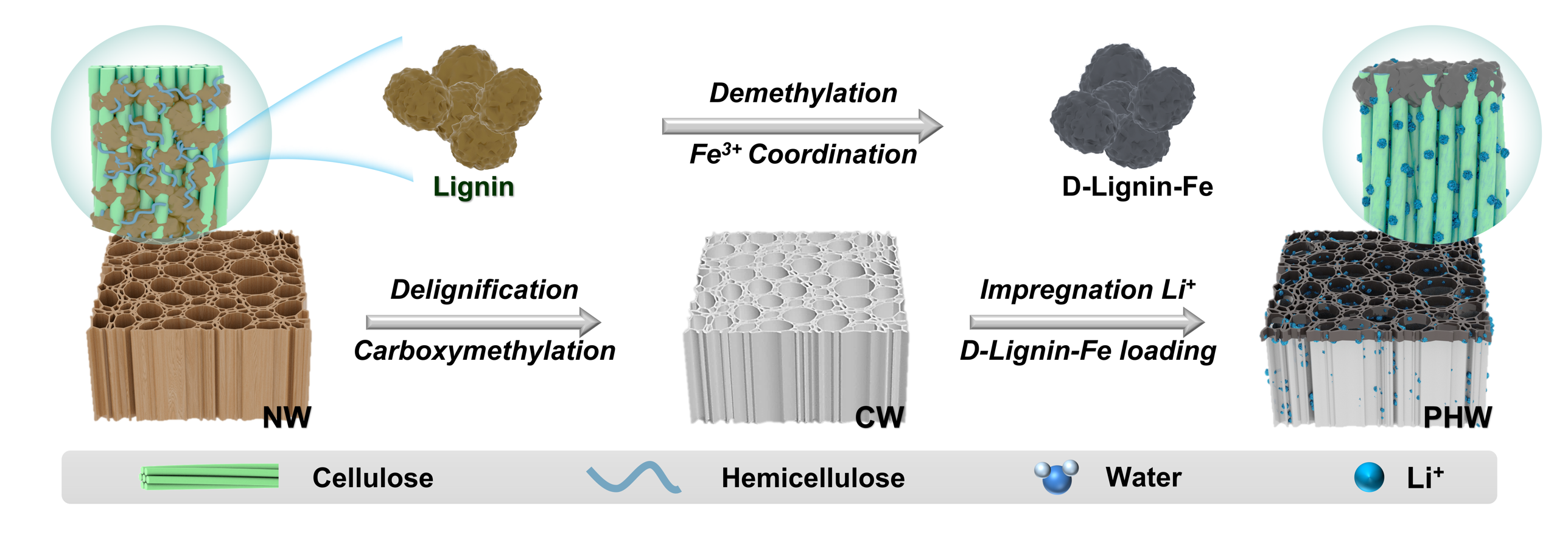

Supplement: Supplementary 1 — Figs. S1 to S39 Tables S1 to S9 Movies S1 to S3 [file research.1195.f1.zip › Supplementary Figures/Figure S1.png]

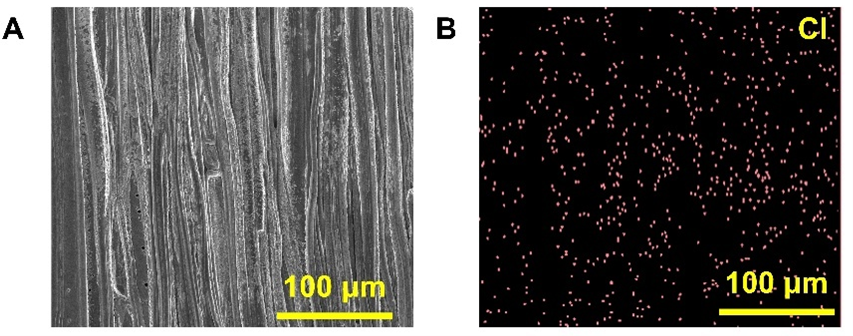

Supplement: Supplementary 1 — Figs. S1 to S39 Tables S1 to S9 Movies S1 to S3 [file research.1195.f1.zip › Supplementary Figures/Figure S10.png]

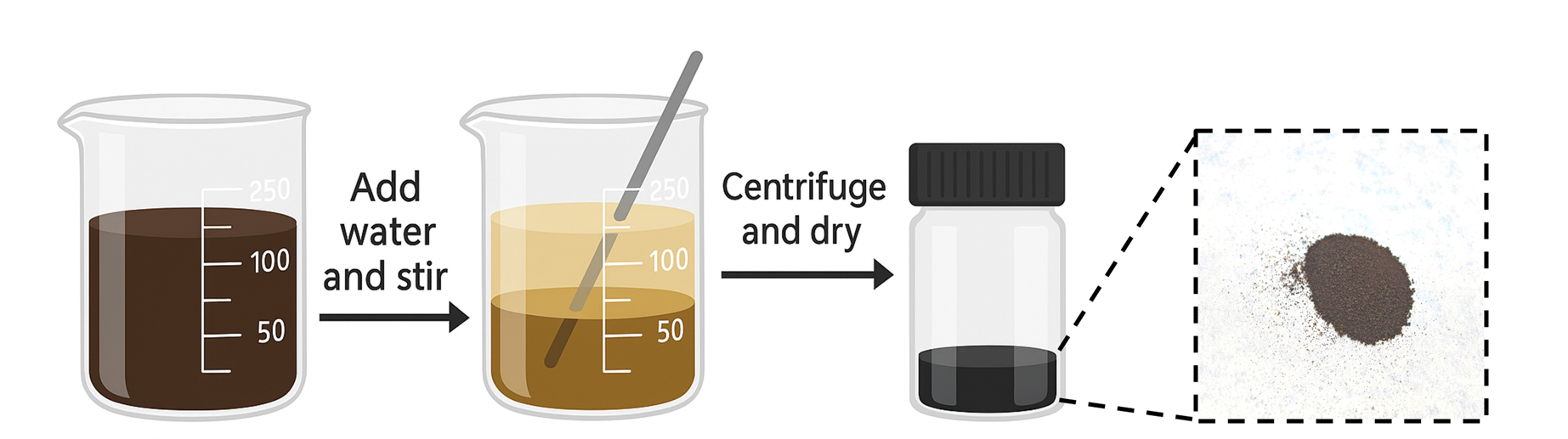

Supplement: Supplementary 1 — Figs. S1 to S39 Tables S1 to S9 Movies S1 to S3 [file research.1195.f1.zip › Supplementary Figures/Figure S11.png]

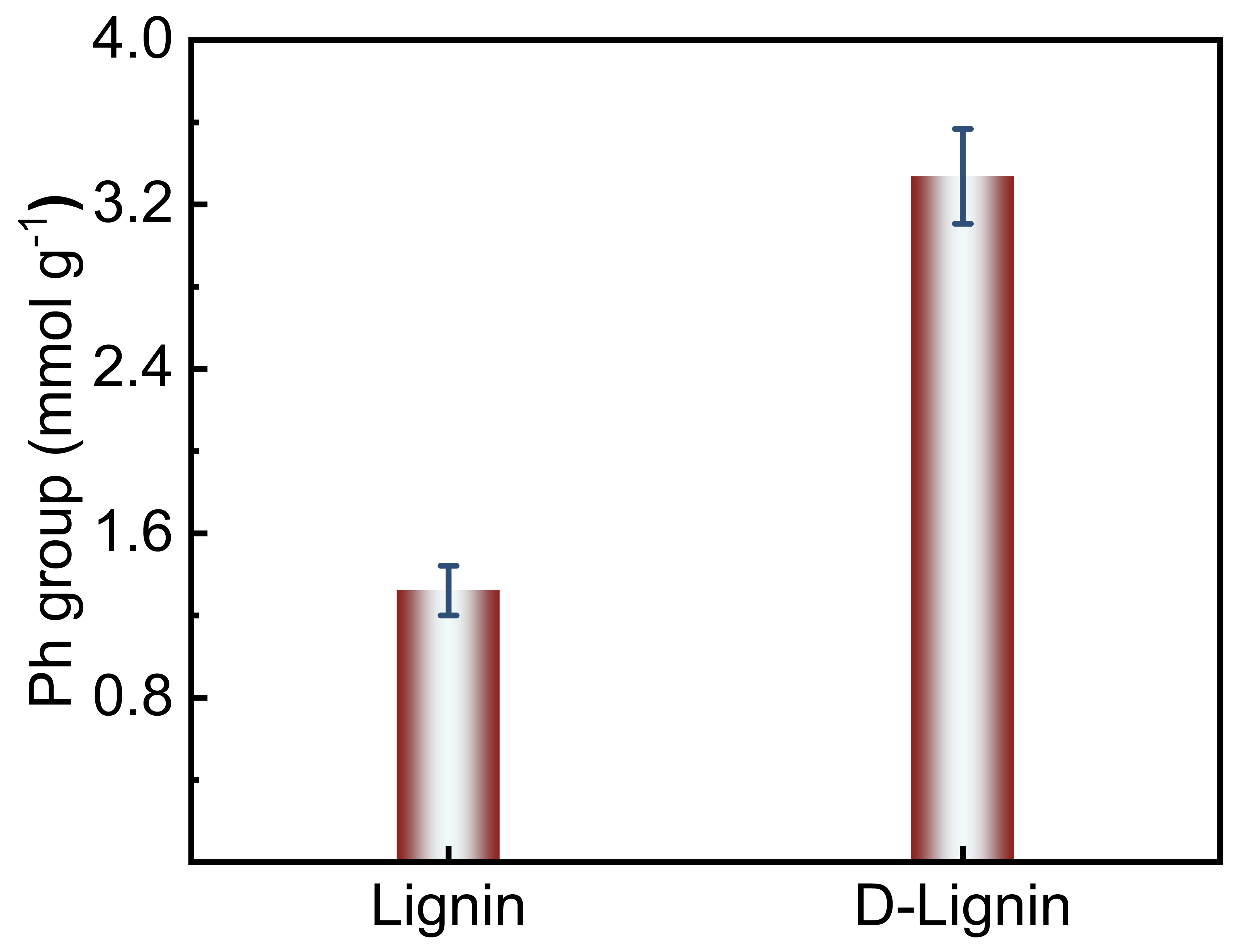

Supplement: Supplementary 1 — Figs. S1 to S39 Tables S1 to S9 Movies S1 to S3 [file research.1195.f1.zip › Supplementary Figures/Figure S12.png]

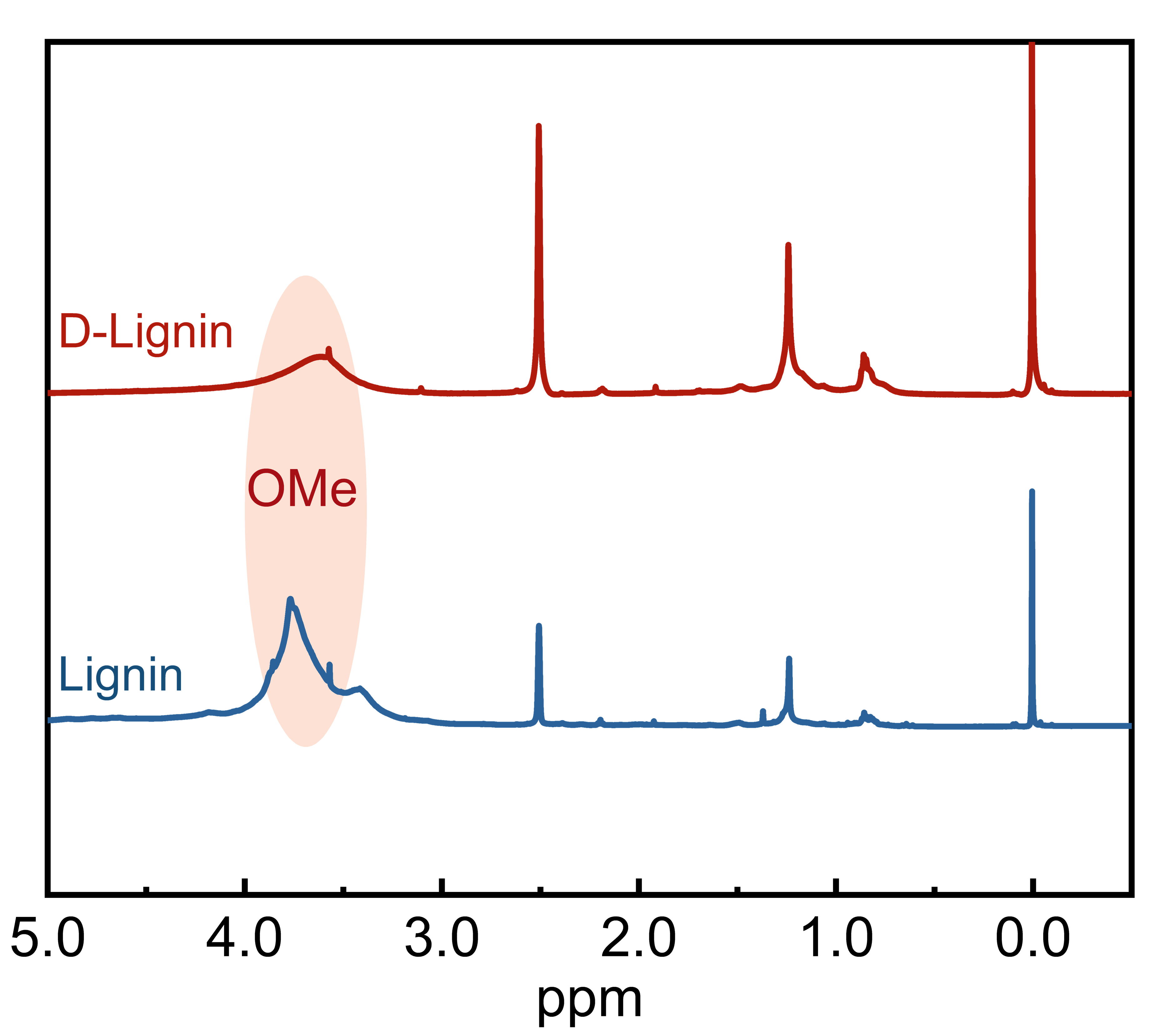

Supplement: Supplementary 1 — Figs. S1 to S39 Tables S1 to S9 Movies S1 to S3 [file research.1195.f1.zip › Supplementary Figures/Figure S13.png]

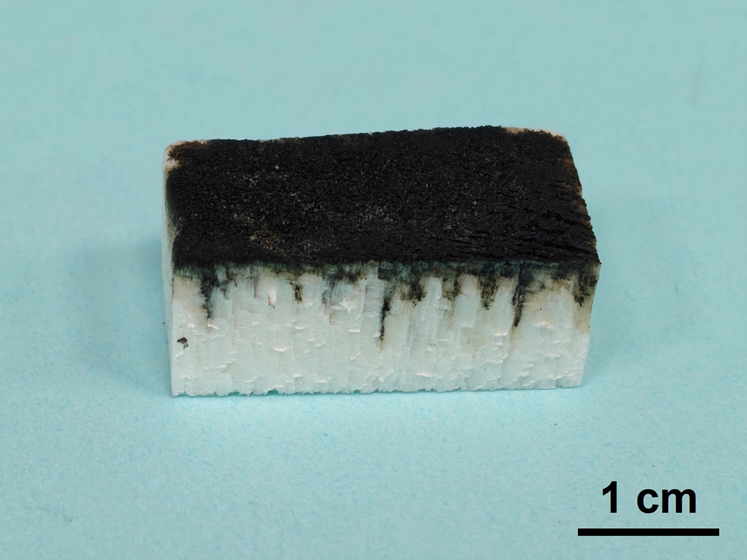

Supplement: Supplementary 1 — Figs. S1 to S39 Tables S1 to S9 Movies S1 to S3 [file research.1195.f1.zip › Supplementary Figures/Figure S14.png]

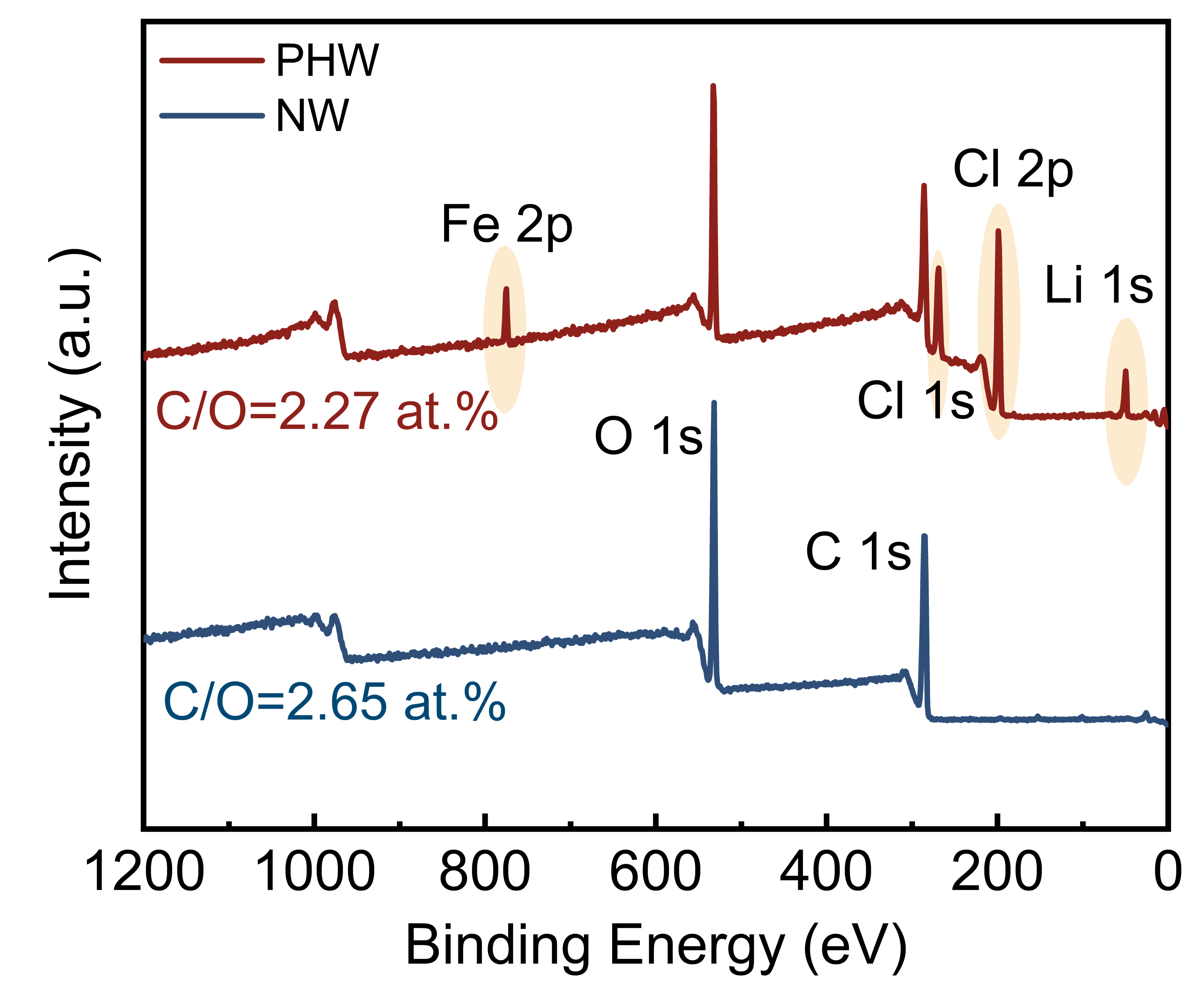

Supplement: Supplementary 1 — Figs. S1 to S39 Tables S1 to S9 Movies S1 to S3 [file research.1195.f1.zip › Supplementary Figures/Figure S15.png]

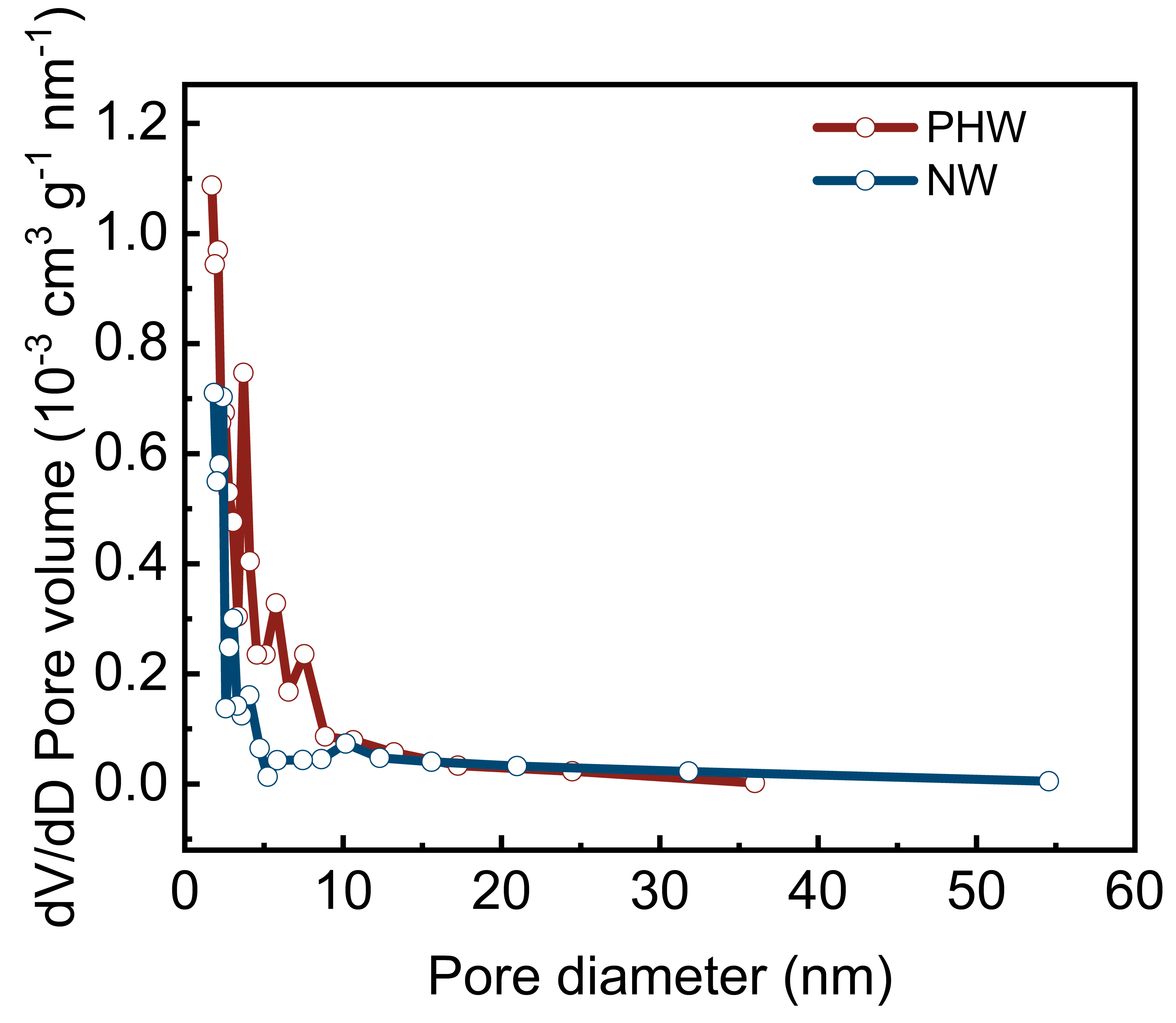

Supplement: Supplementary 1 — Figs. S1 to S39 Tables S1 to S9 Movies S1 to S3 [file research.1195.f1.zip › Supplementary Figures/Figure S16.png]

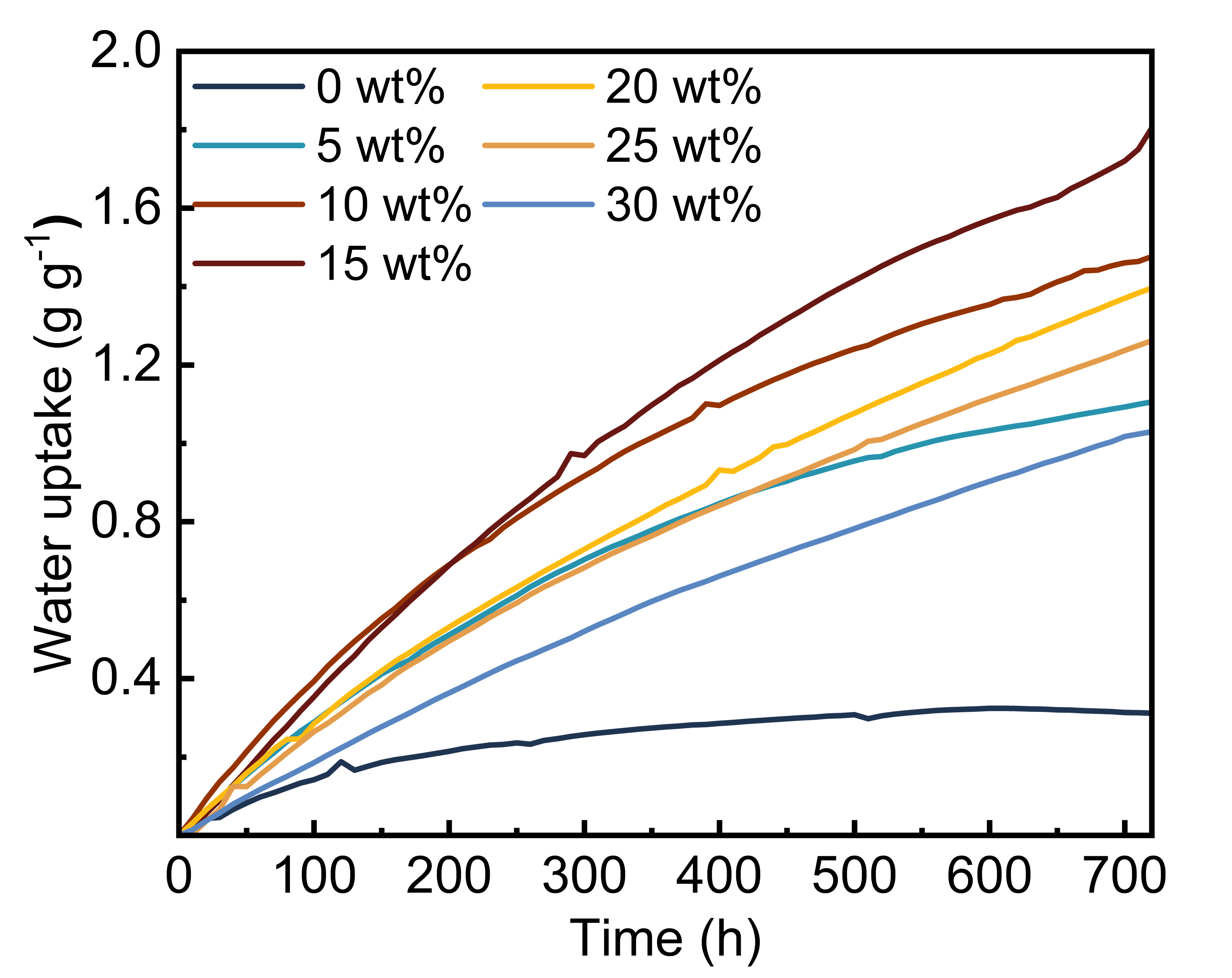

Supplement: Supplementary 1 — Figs. S1 to S39 Tables S1 to S9 Movies S1 to S3 [file research.1195.f1.zip › Supplementary Figures/Figure S17.png]

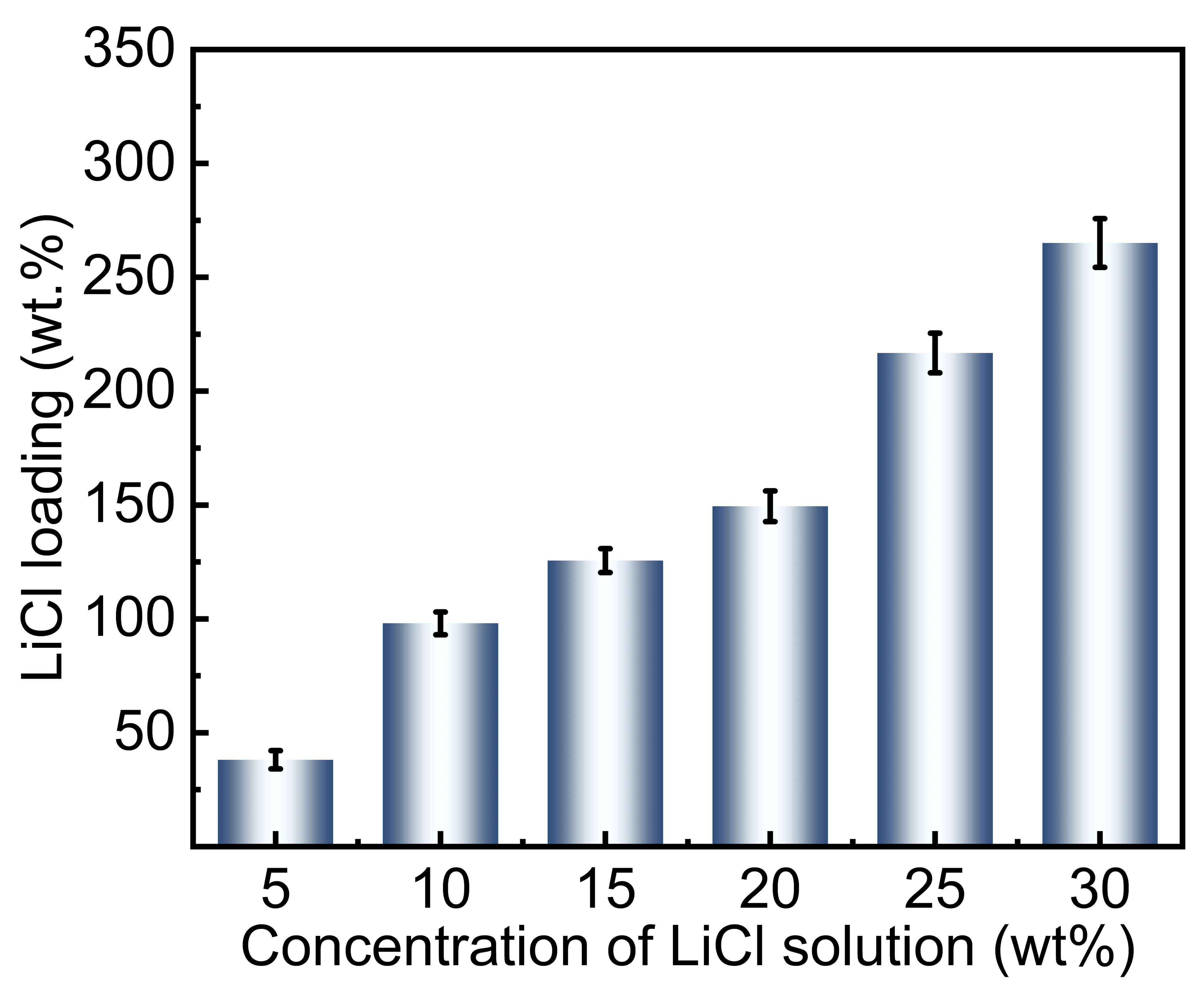

Supplement: Supplementary 1 — Figs. S1 to S39 Tables S1 to S9 Movies S1 to S3 [file research.1195.f1.zip › Supplementary Figures/Figure S18.png]

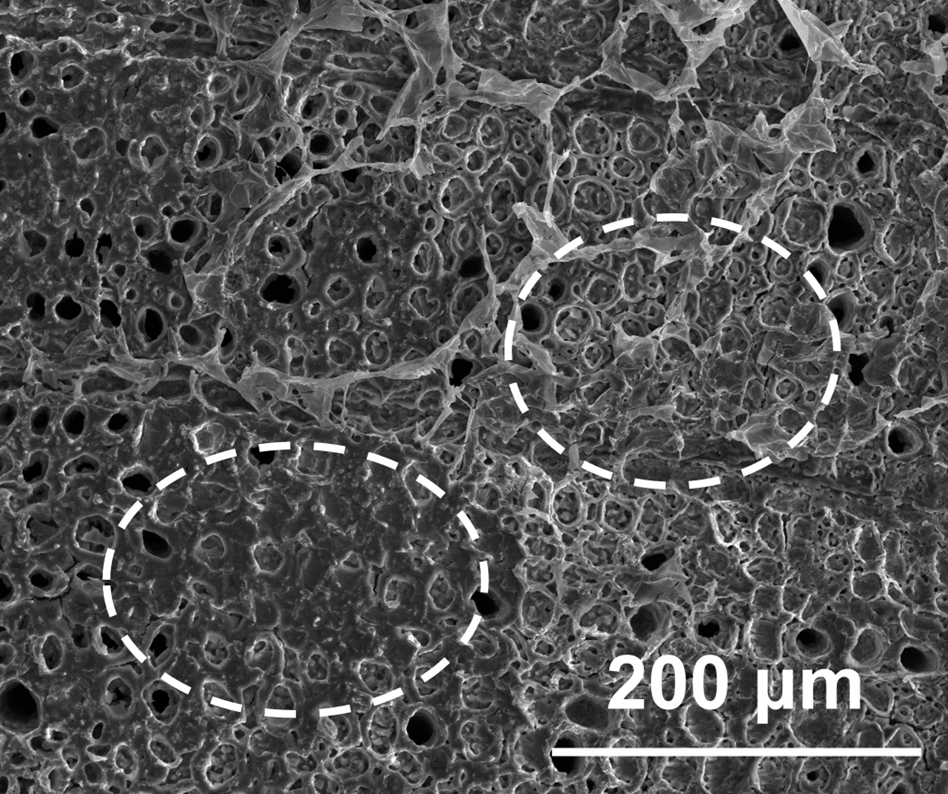

Supplement: Supplementary 1 — Figs. S1 to S39 Tables S1 to S9 Movies S1 to S3 [file research.1195.f1.zip › Supplementary Figures/Figure S19.png]

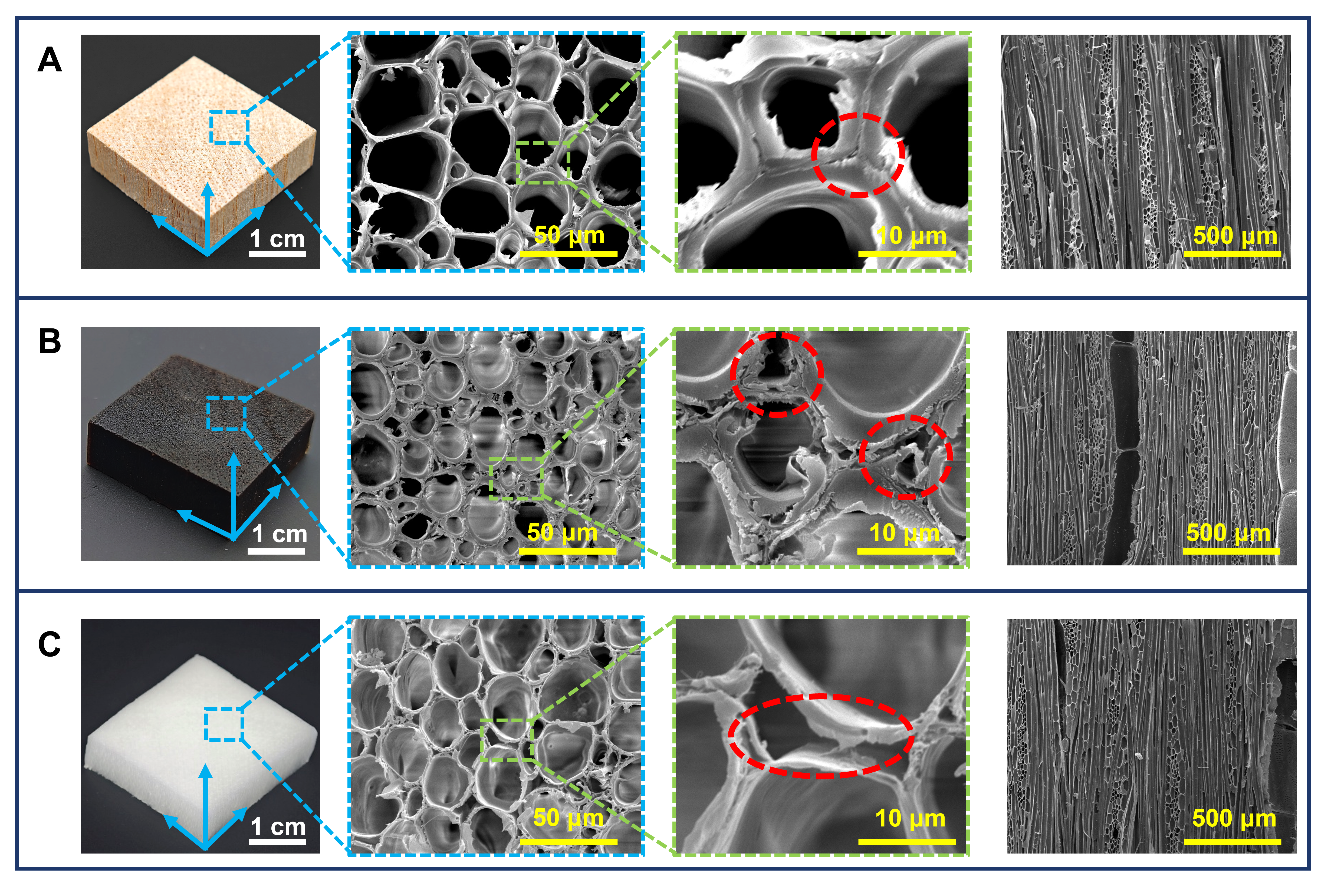

Supplement: Supplementary 1 — Figs. S1 to S39 Tables S1 to S9 Movies S1 to S3 [file research.1195.f1.zip › Supplementary Figures/Figure S2.png]

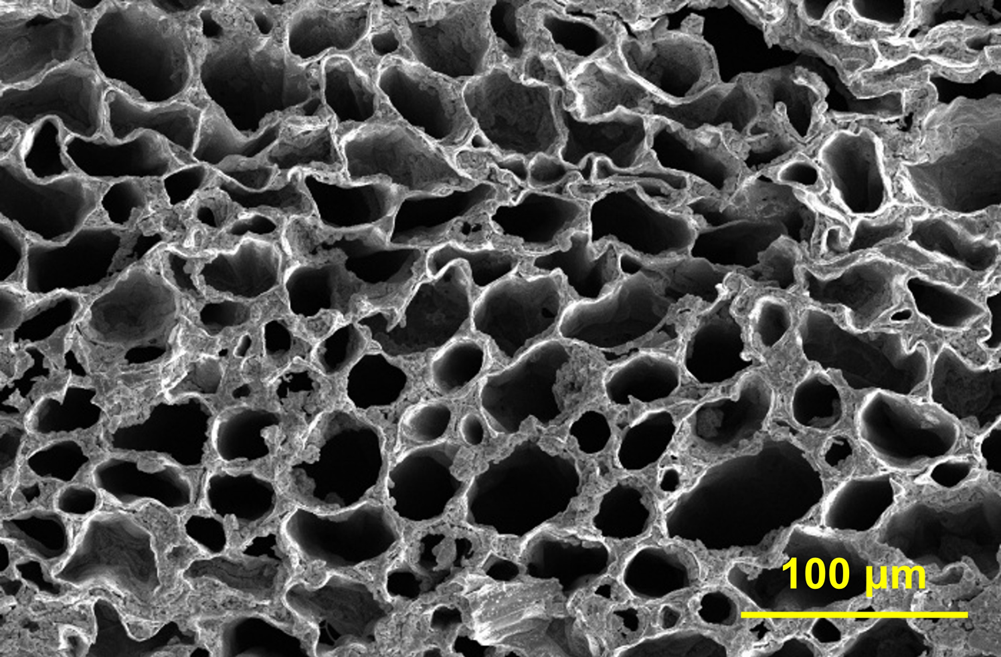

Supplement: Supplementary 1 — Figs. S1 to S39 Tables S1 to S9 Movies S1 to S3 [file research.1195.f1.zip › Supplementary Figures/Figure S20.png]

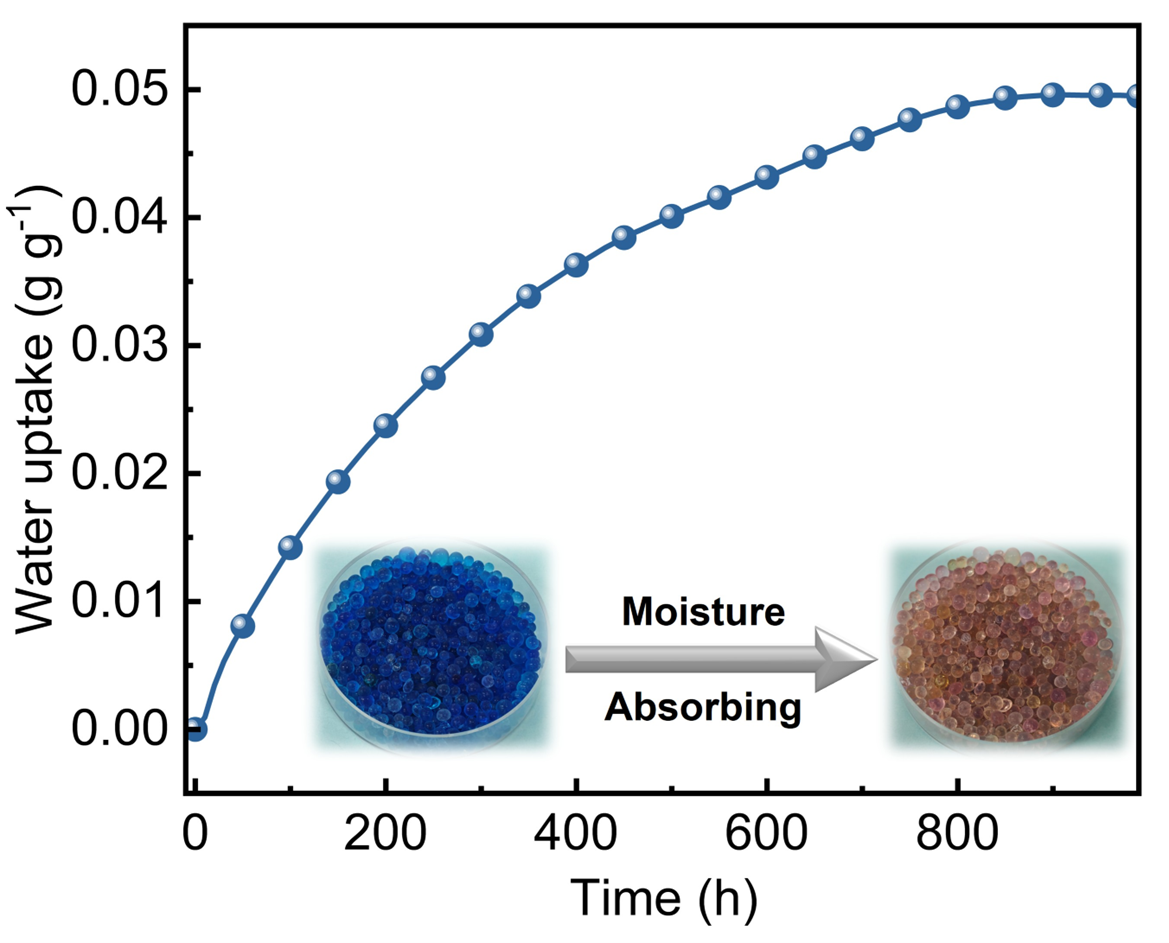

Supplement: Supplementary 1 — Figs. S1 to S39 Tables S1 to S9 Movies S1 to S3 [file research.1195.f1.zip › Supplementary Figures/Figure S21.png]

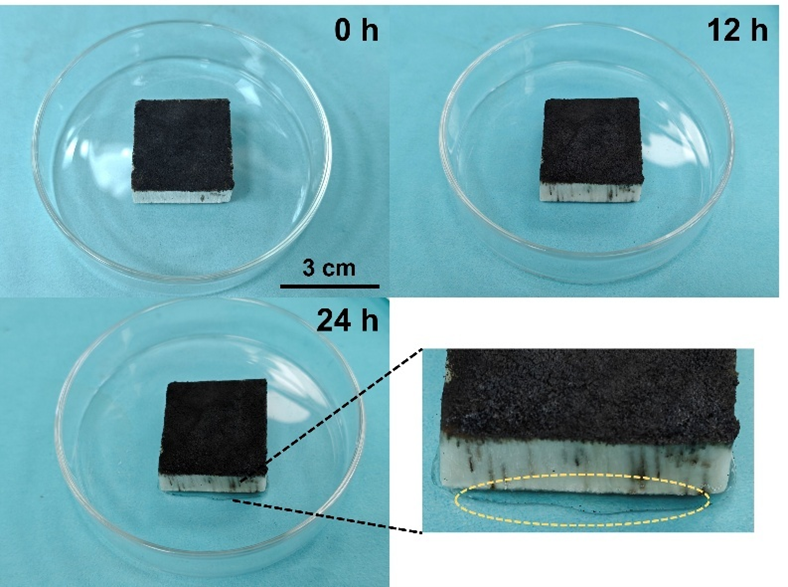

Supplement: Supplementary 1 — Figs. S1 to S39 Tables S1 to S9 Movies S1 to S3 [file research.1195.f1.zip › Supplementary Figures/Figure S22.png]

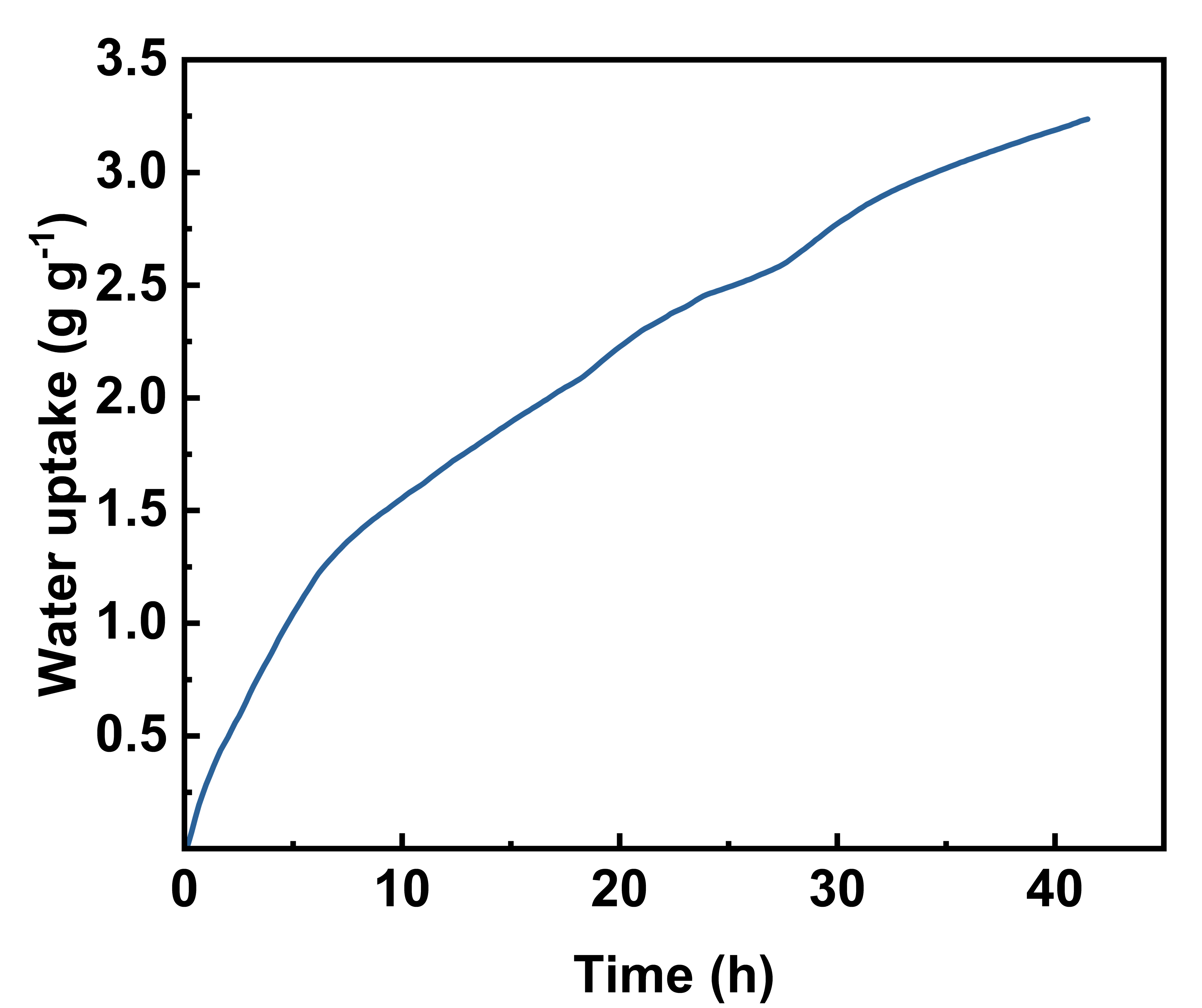

Supplement: Supplementary 1 — Figs. S1 to S39 Tables S1 to S9 Movies S1 to S3 [file research.1195.f1.zip › Supplementary Figures/Figure S23.png]

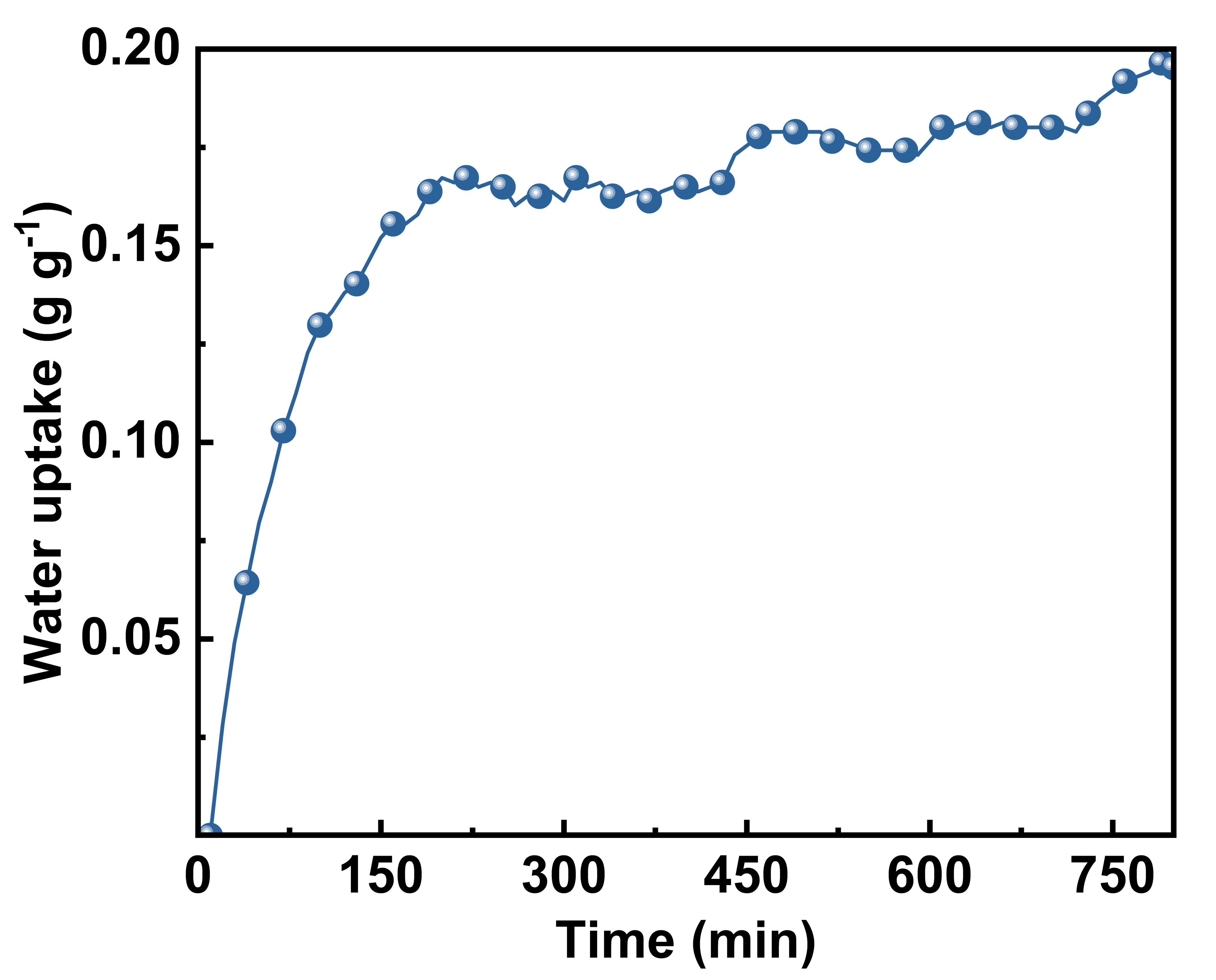

Supplement: Supplementary 1 — Figs. S1 to S39 Tables S1 to S9 Movies S1 to S3 [file research.1195.f1.zip › Supplementary Figures/Figure S24.png]

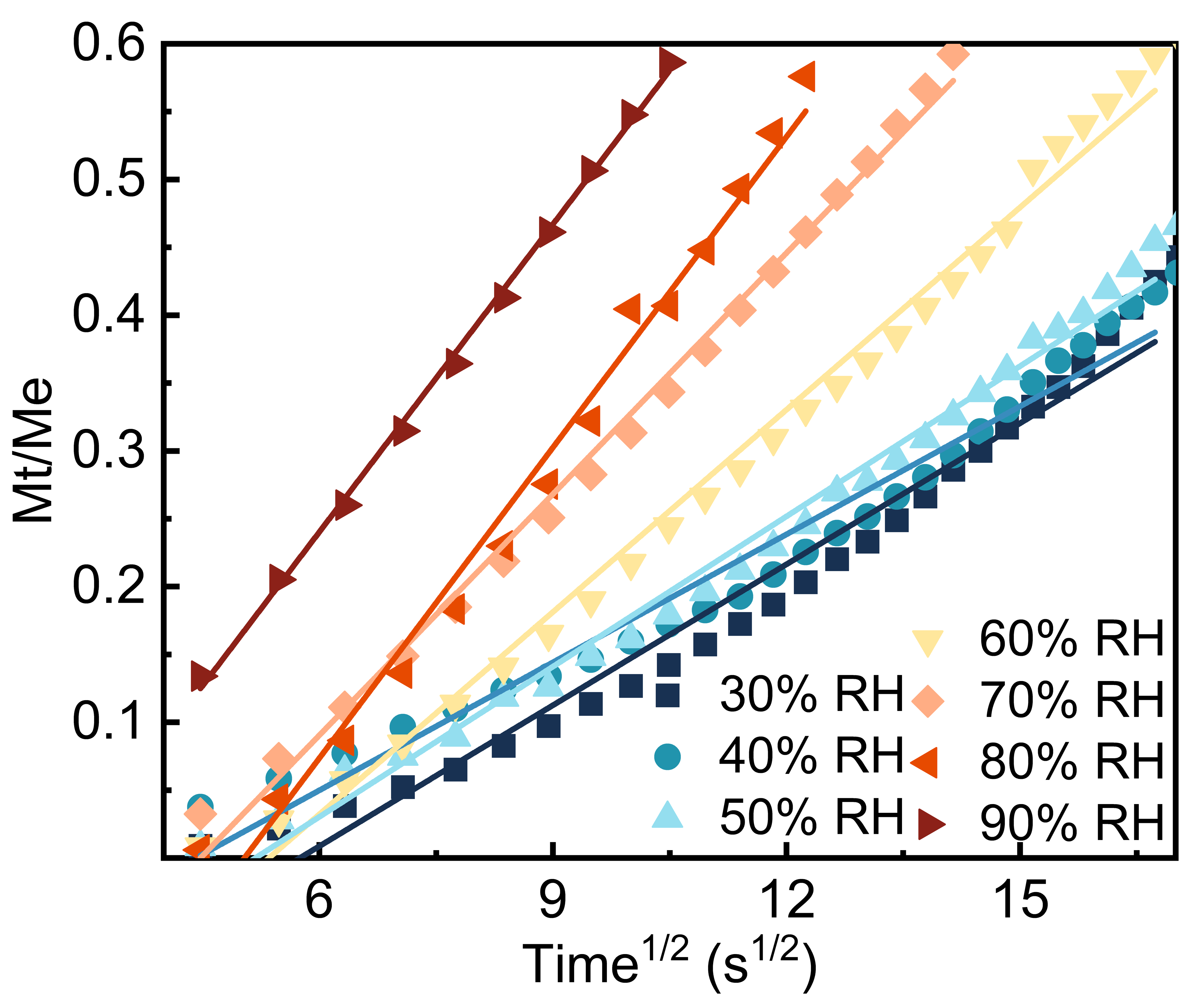

Supplement: Supplementary 1 — Figs. S1 to S39 Tables S1 to S9 Movies S1 to S3 [file research.1195.f1.zip › Supplementary Figures/Figure S25.png]

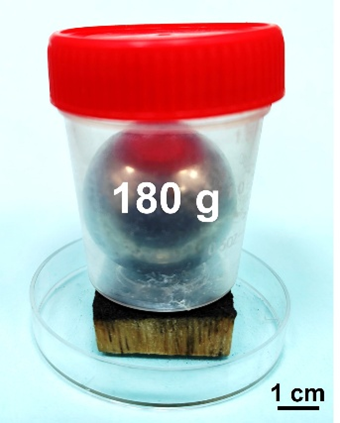

Supplement: Supplementary 1 — Figs. S1 to S39 Tables S1 to S9 Movies S1 to S3 [file research.1195.f1.zip › Supplementary Figures/Figure S26.png]

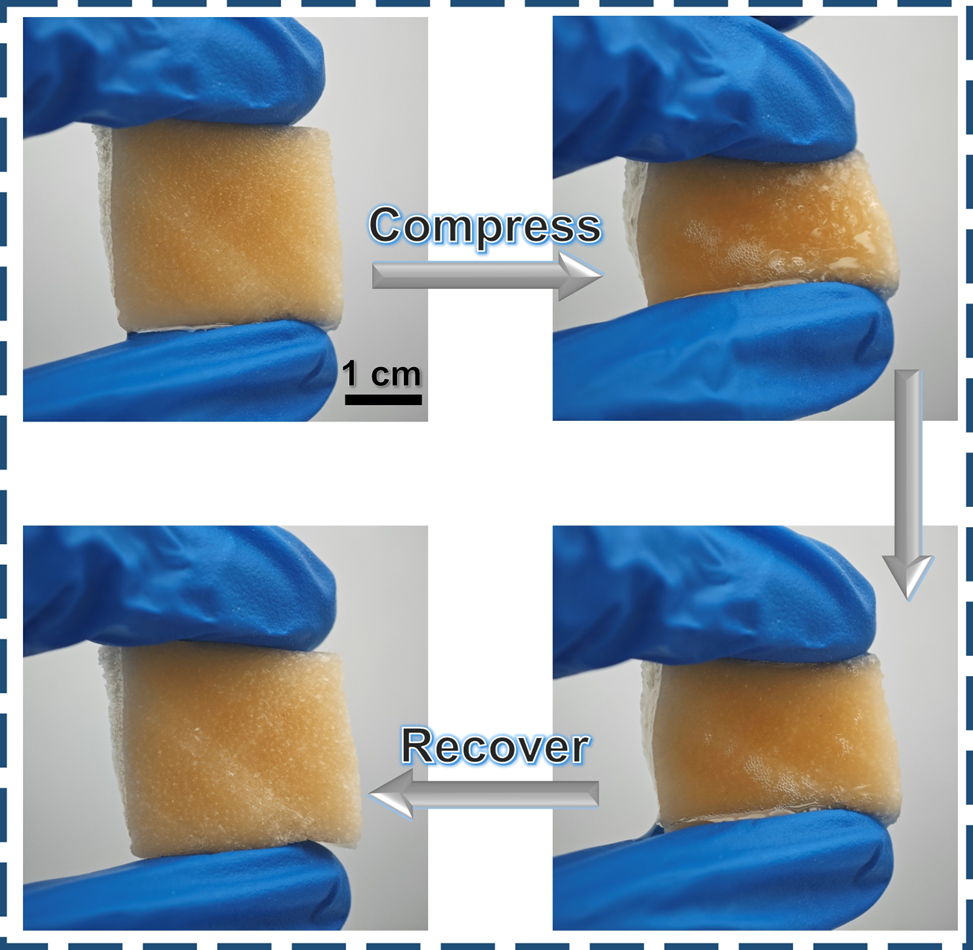

Supplement: Supplementary 1 — Figs. S1 to S39 Tables S1 to S9 Movies S1 to S3 [file research.1195.f1.zip › Supplementary Figures/Figure S27.png]

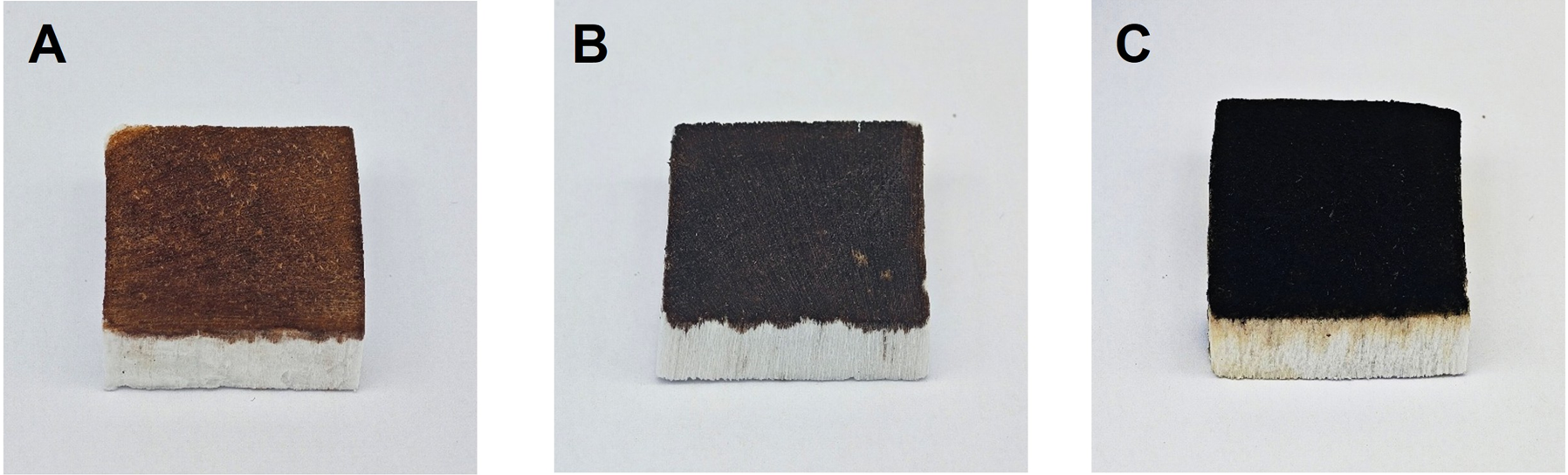

Supplement: Supplementary 1 — Figs. S1 to S39 Tables S1 to S9 Movies S1 to S3 [file research.1195.f1.zip › Supplementary Figures/Figure S28.png]

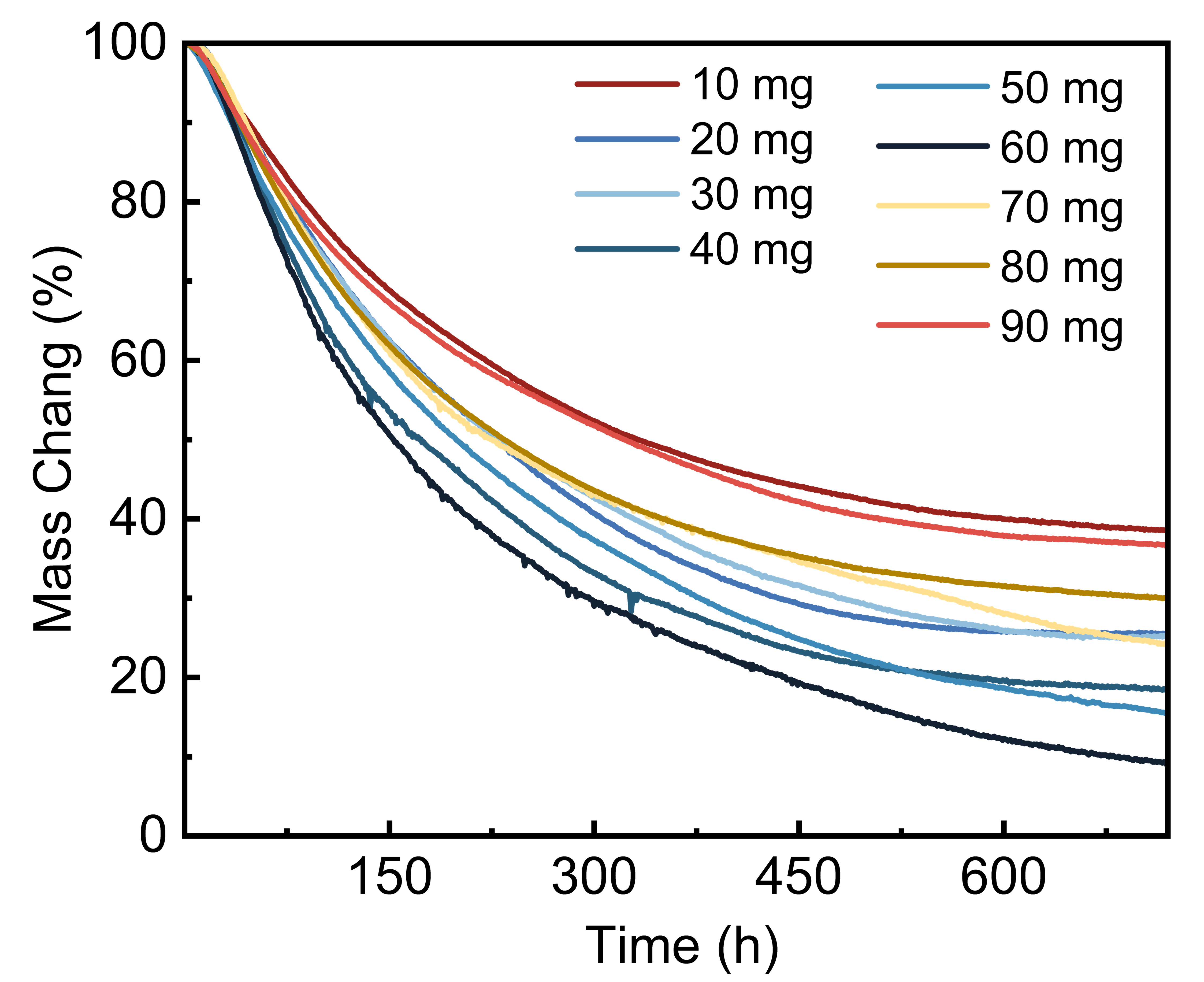

Supplement: Supplementary 1 — Figs. S1 to S39 Tables S1 to S9 Movies S1 to S3 [file research.1195.f1.zip › Supplementary Figures/Figure S29.png]

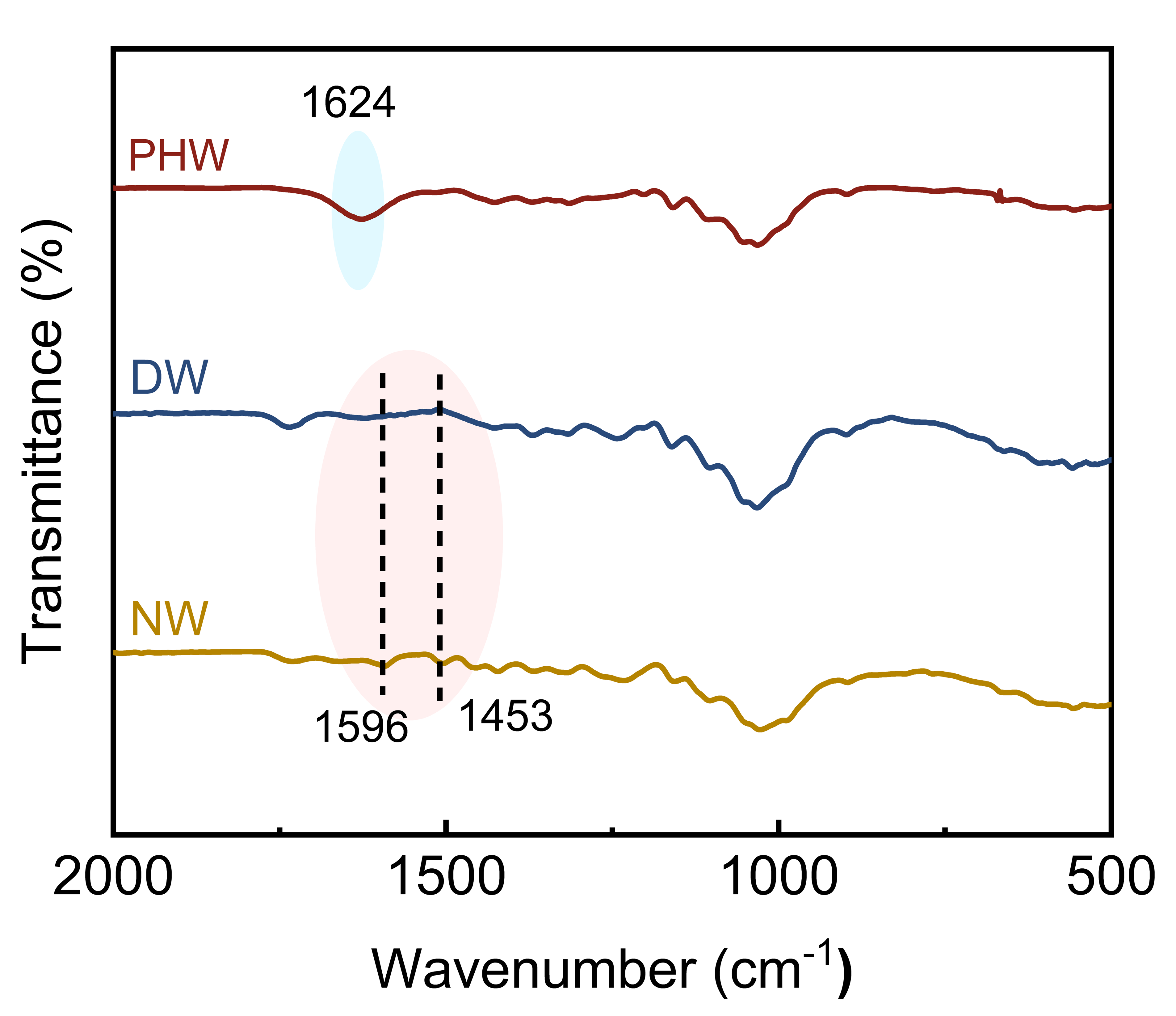

Supplement: Supplementary 1 — Figs. S1 to S39 Tables S1 to S9 Movies S1 to S3 [file research.1195.f1.zip › Supplementary Figures/Figure S3.png]

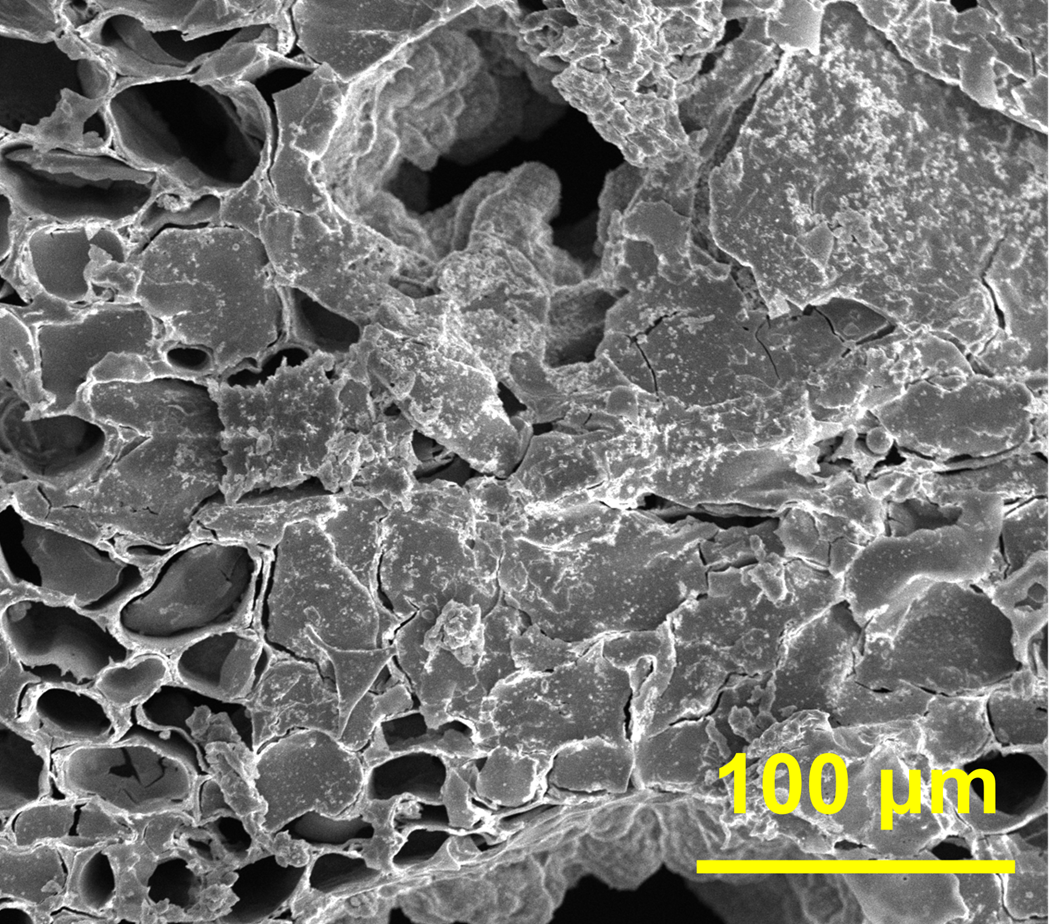

Supplement: Supplementary 1 — Figs. S1 to S39 Tables S1 to S9 Movies S1 to S3 [file research.1195.f1.zip › Supplementary Figures/Figure S30.png]

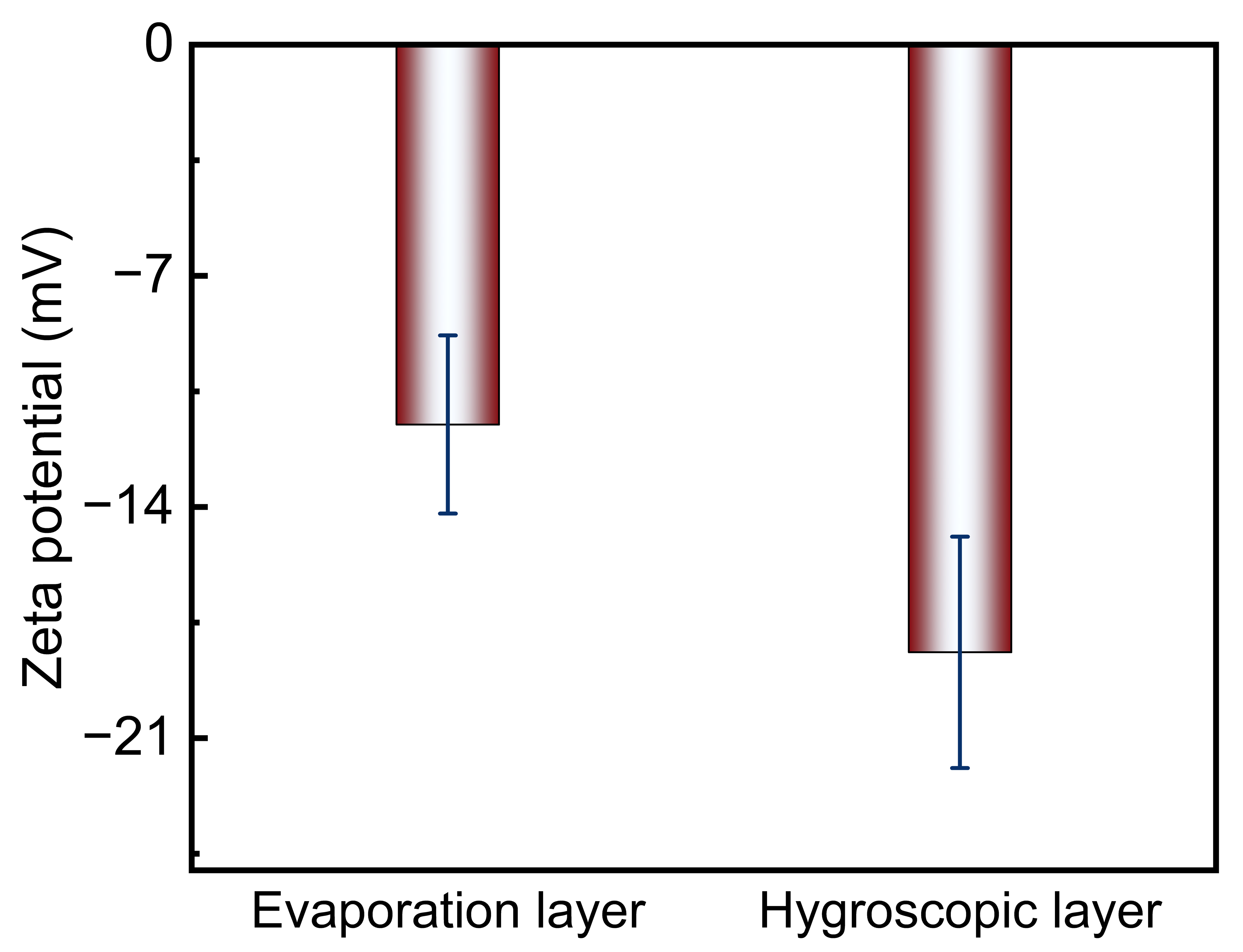

Supplement: Supplementary 1 — Figs. S1 to S39 Tables S1 to S9 Movies S1 to S3 [file research.1195.f1.zip › Supplementary Figures/Figure S31.png]

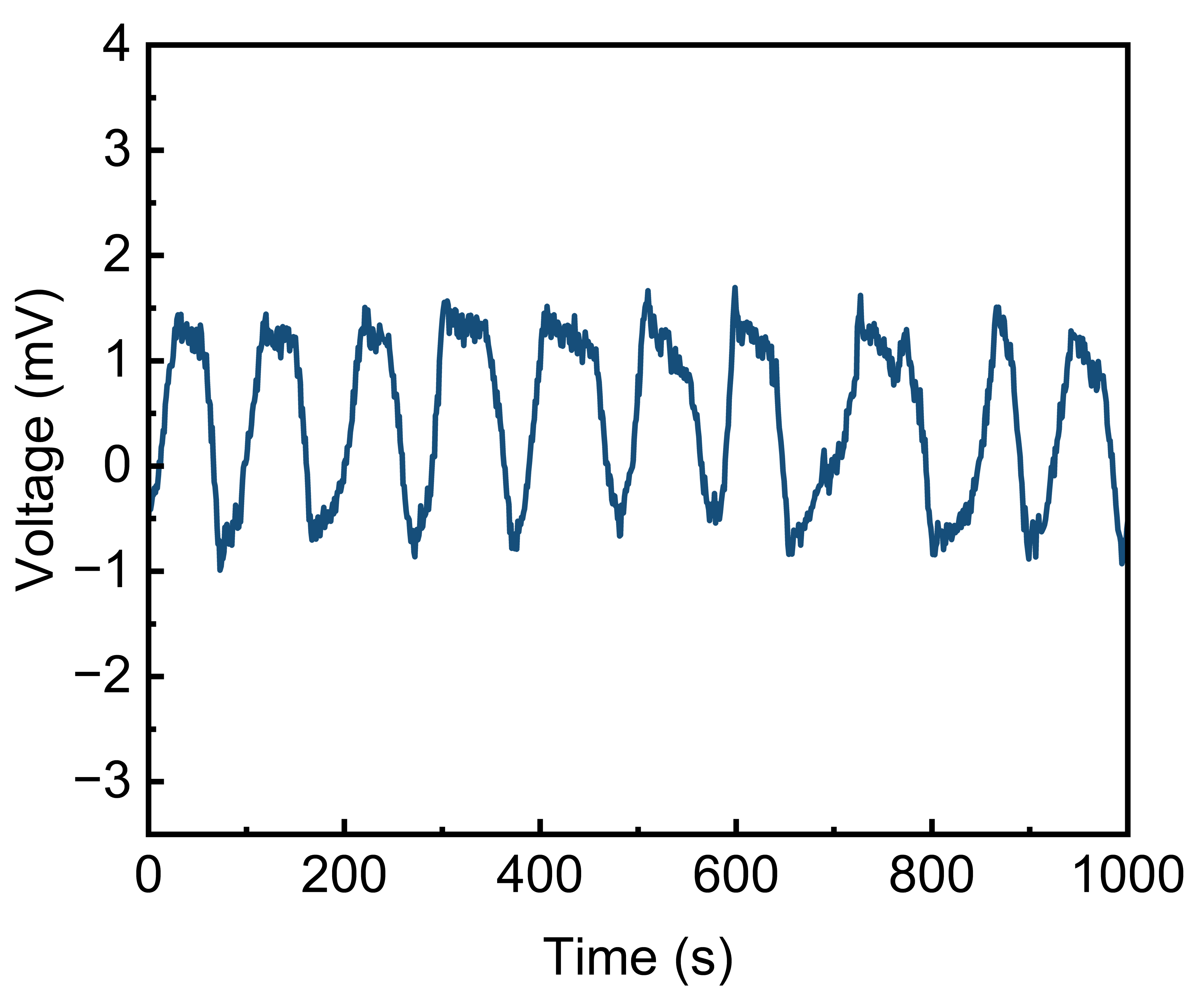

Supplement: Supplementary 1 — Figs. S1 to S39 Tables S1 to S9 Movies S1 to S3 [file research.1195.f1.zip › Supplementary Figures/Figure S32.png]

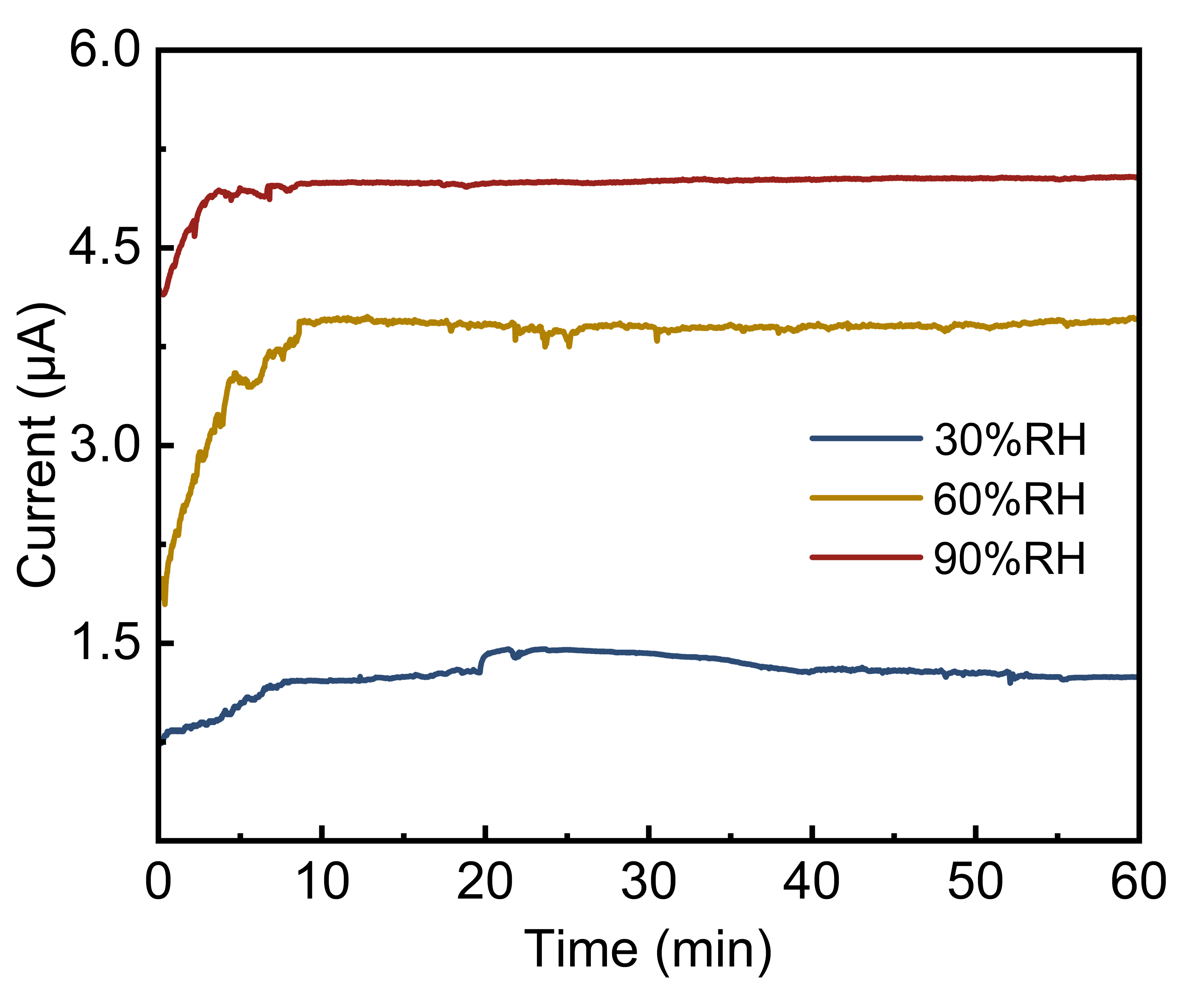

Supplement: Supplementary 1 — Figs. S1 to S39 Tables S1 to S9 Movies S1 to S3 [file research.1195.f1.zip › Supplementary Figures/Figure S33.png]

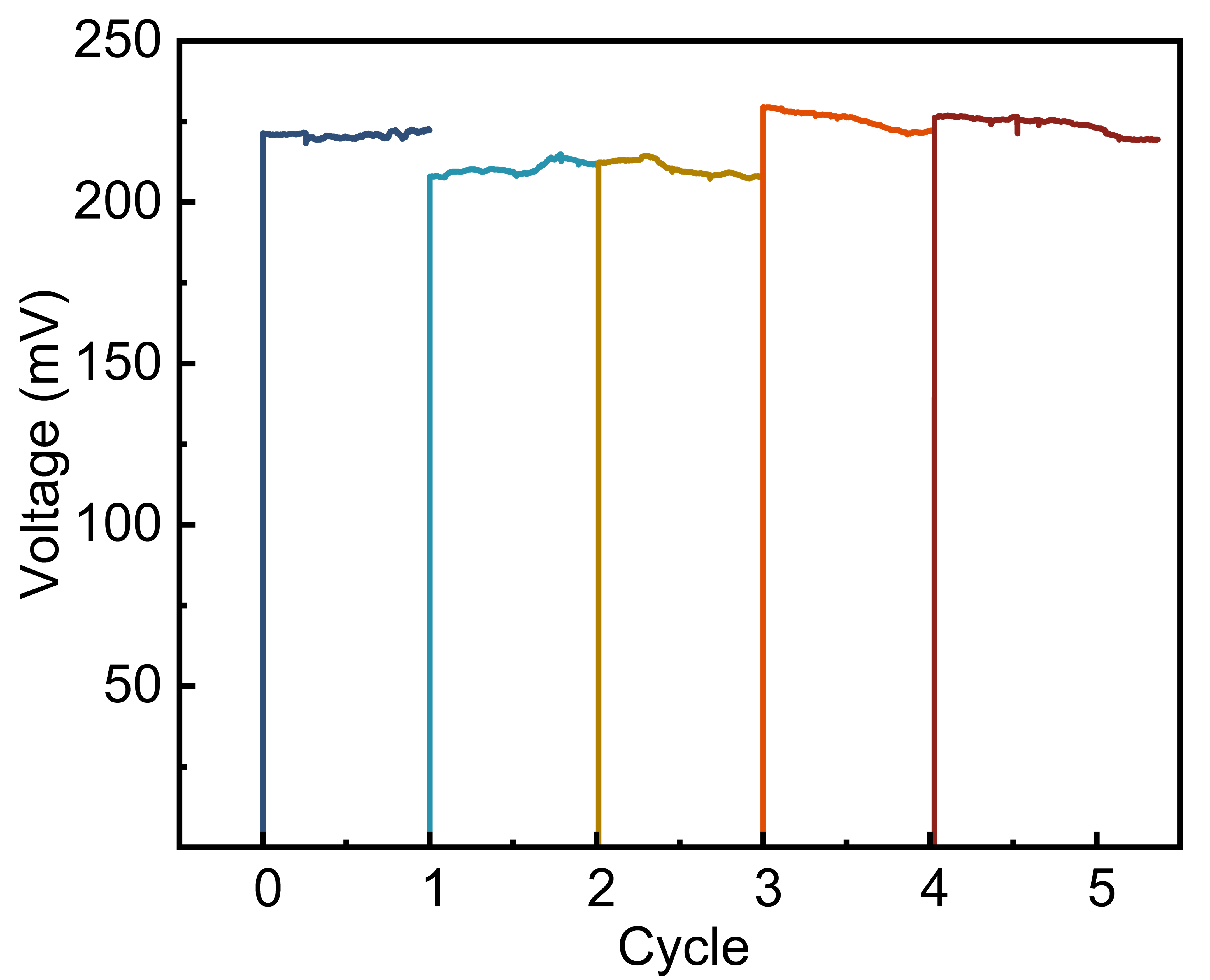

Supplement: Supplementary 1 — Figs. S1 to S39 Tables S1 to S9 Movies S1 to S3 [file research.1195.f1.zip › Supplementary Figures/Figure S34.png]

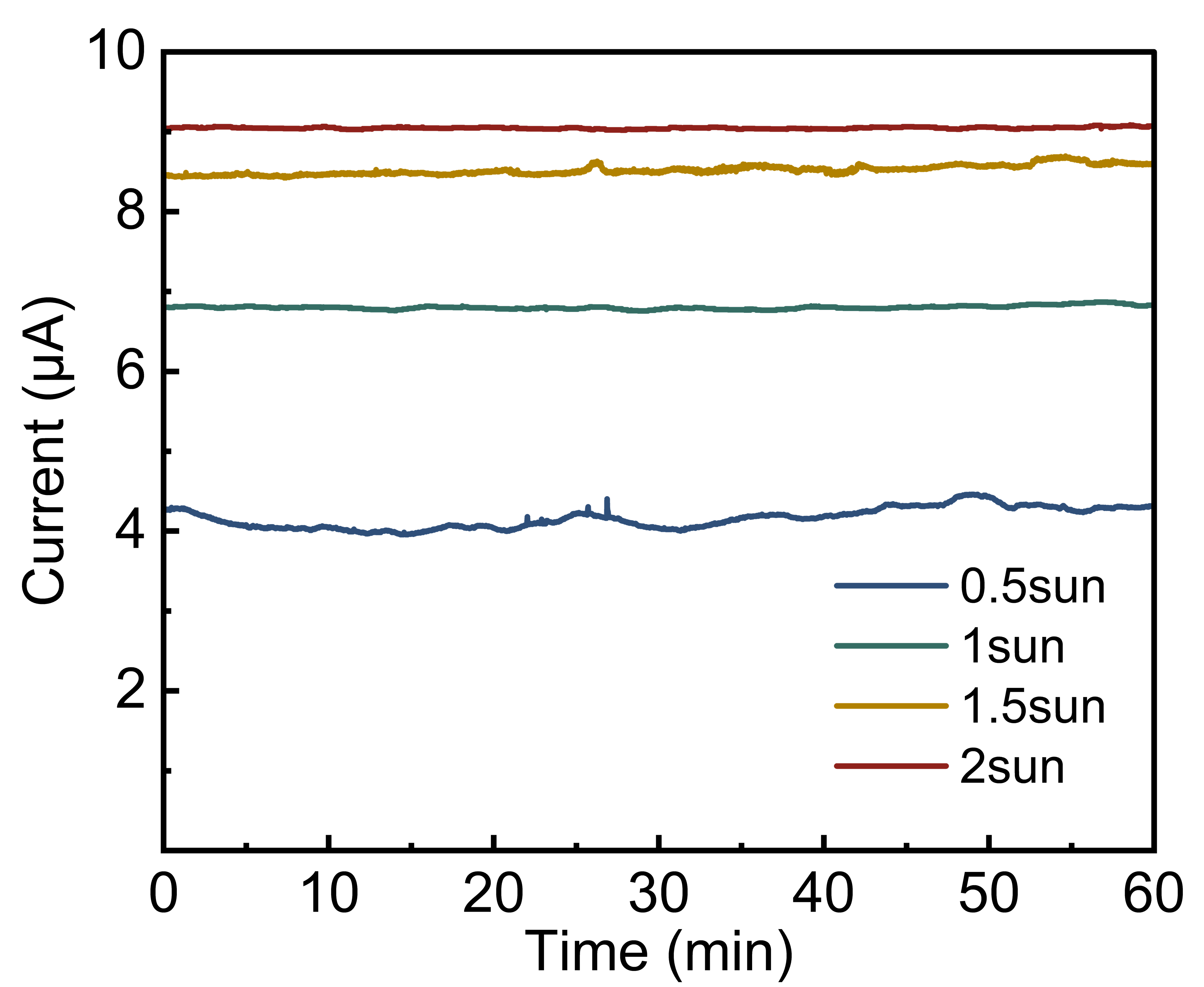

Supplement: Supplementary 1 — Figs. S1 to S39 Tables S1 to S9 Movies S1 to S3 [file research.1195.f1.zip › Supplementary Figures/Figure S35.png]

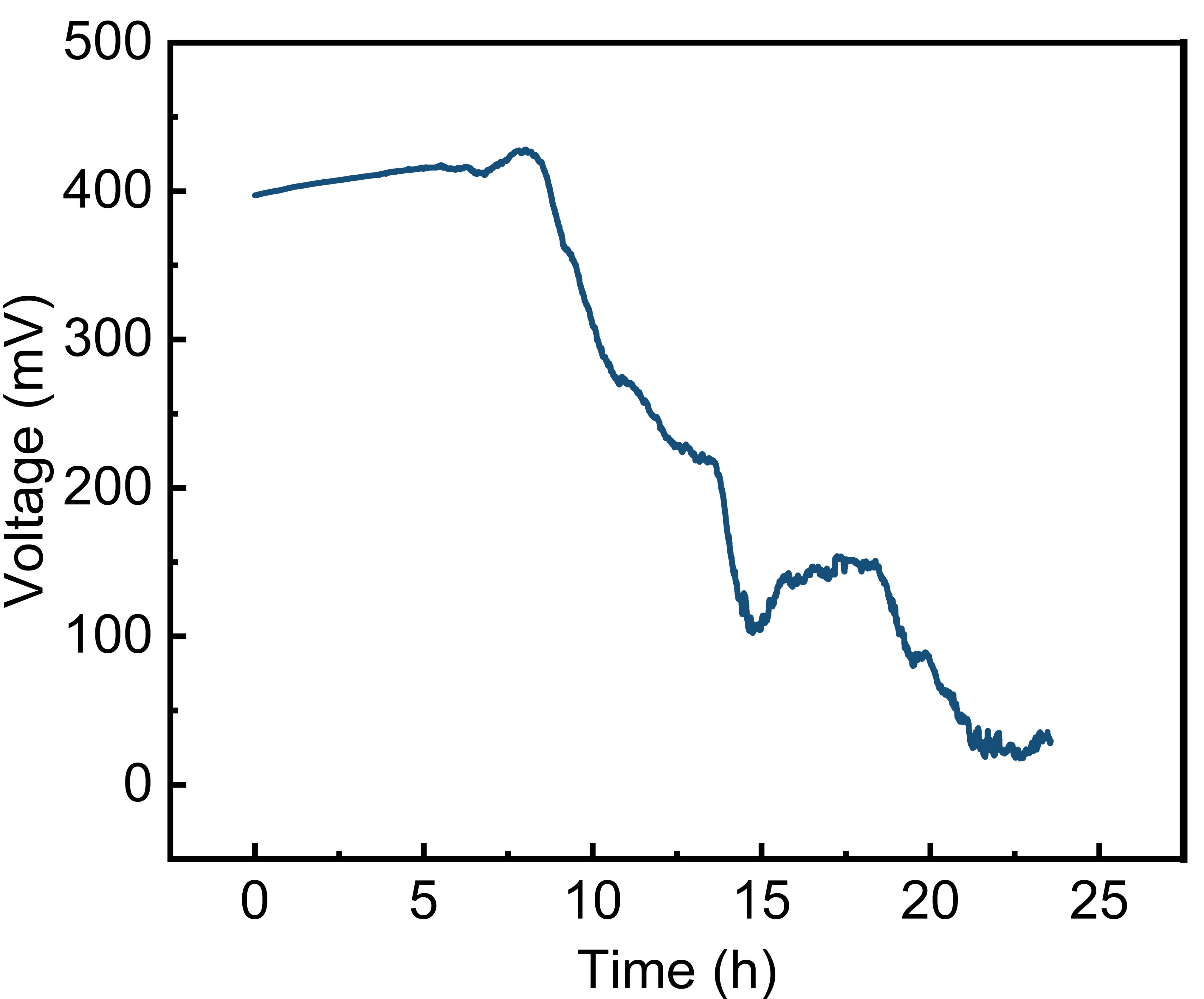

Supplement: Supplementary 1 — Figs. S1 to S39 Tables S1 to S9 Movies S1 to S3 [file research.1195.f1.zip › Supplementary Figures/Figure S36.png]

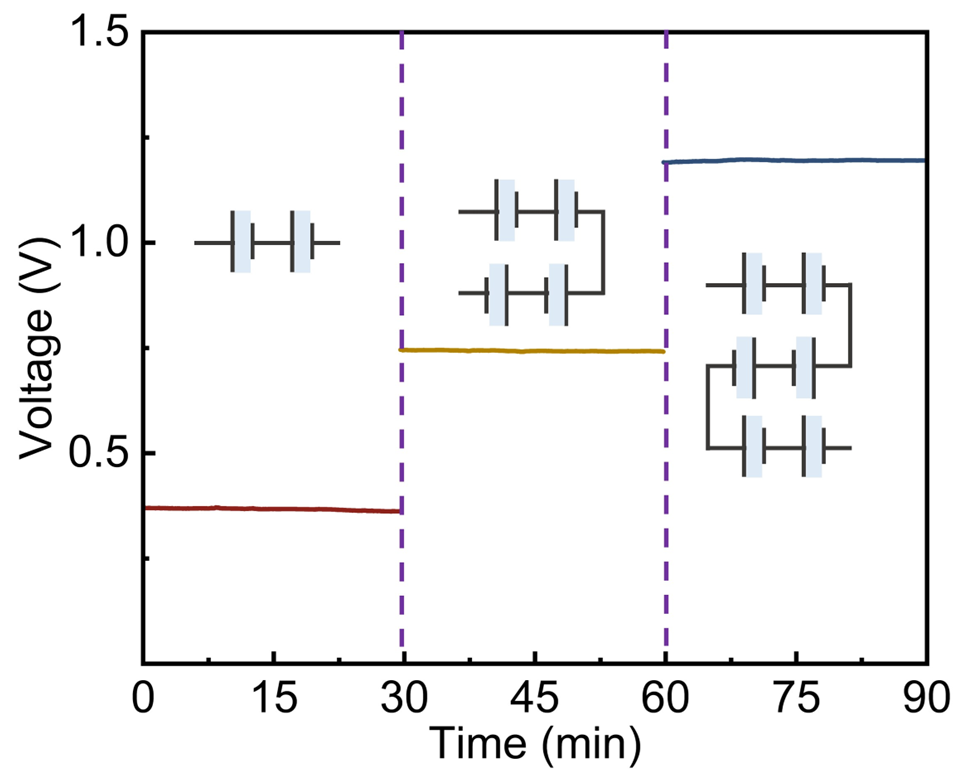

Supplement: Supplementary 1 — Figs. S1 to S39 Tables S1 to S9 Movies S1 to S3 [file research.1195.f1.zip › Supplementary Figures/Figure S37.png]

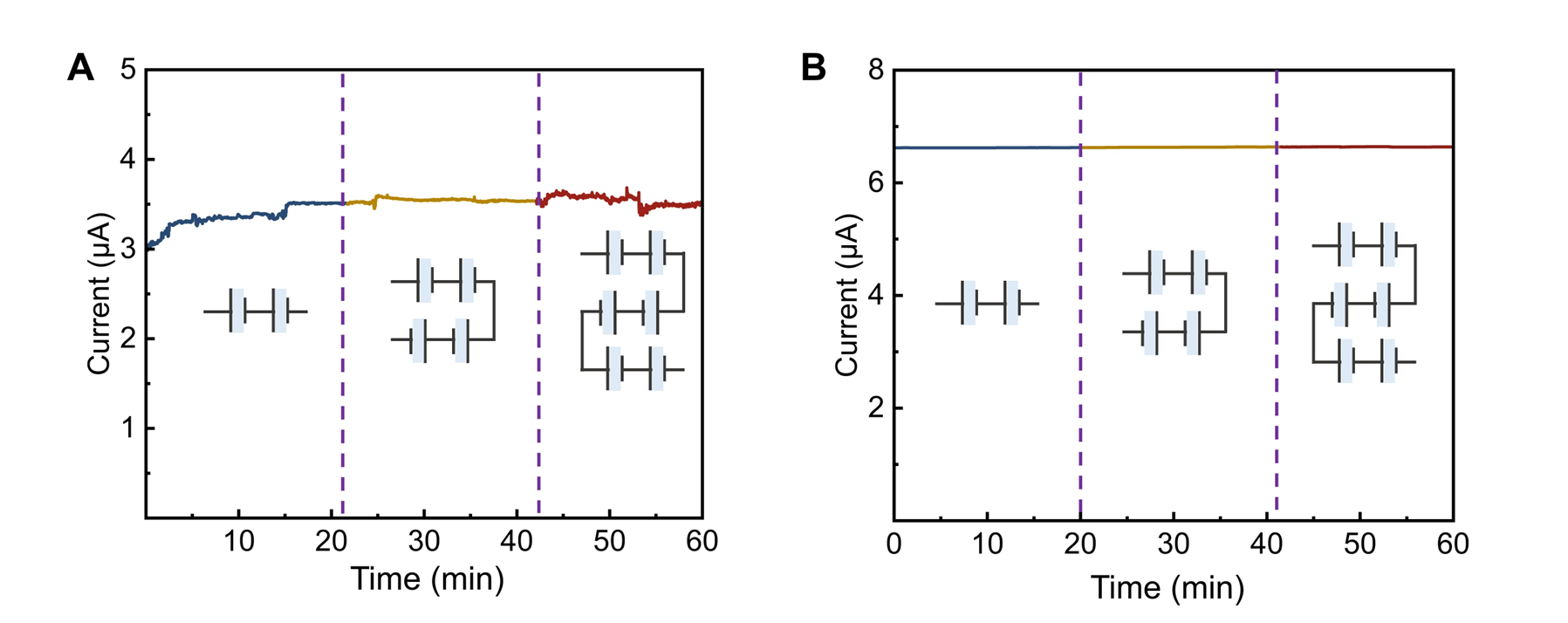

Supplement: Supplementary 1 — Figs. S1 to S39 Tables S1 to S9 Movies S1 to S3 [file research.1195.f1.zip › Supplementary Figures/Figure S38.png]

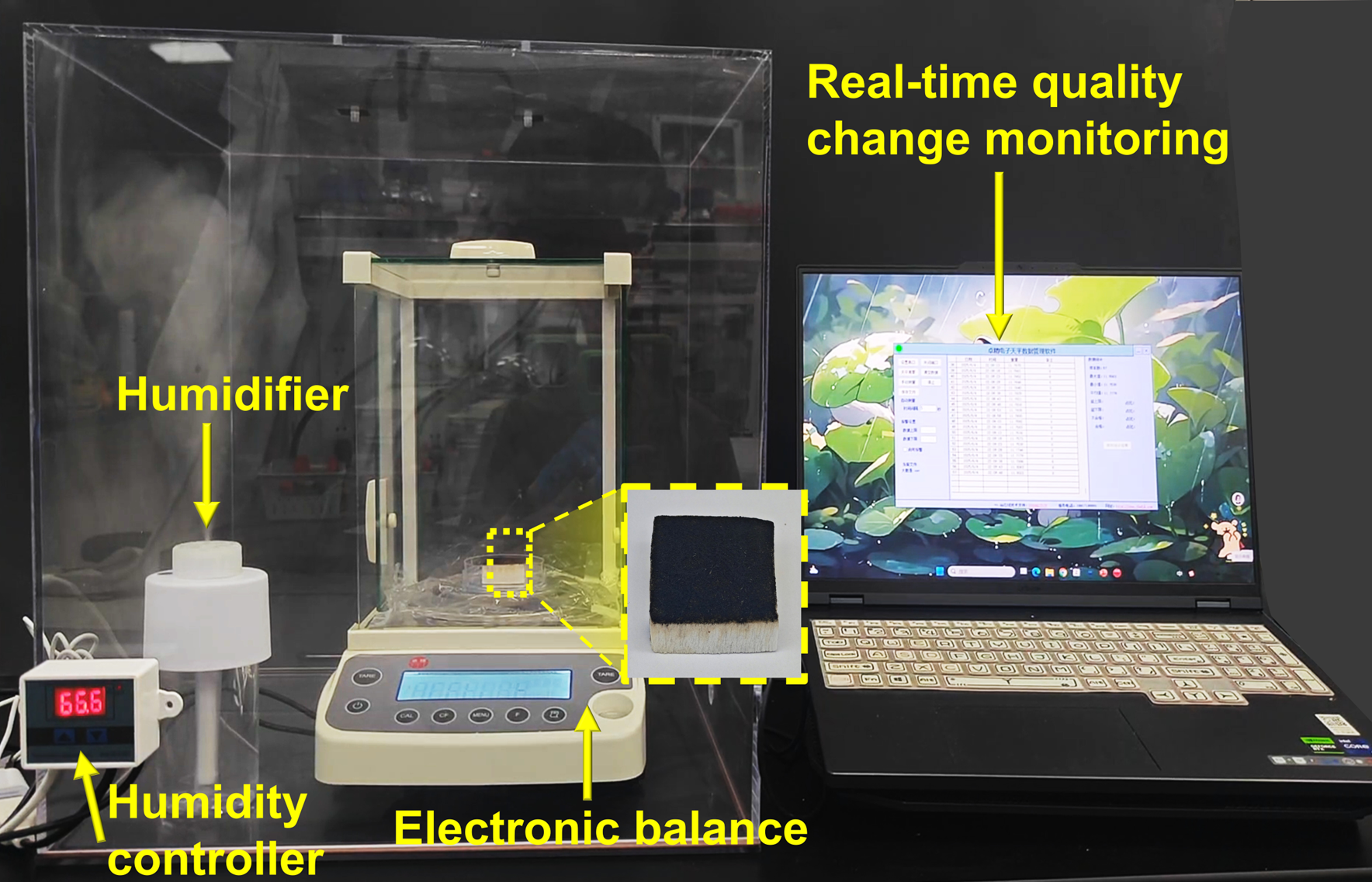

Supplement: Supplementary 1 — Figs. S1 to S39 Tables S1 to S9 Movies S1 to S3 [file research.1195.f1.zip › Supplementary Figures/Figure S39.png]

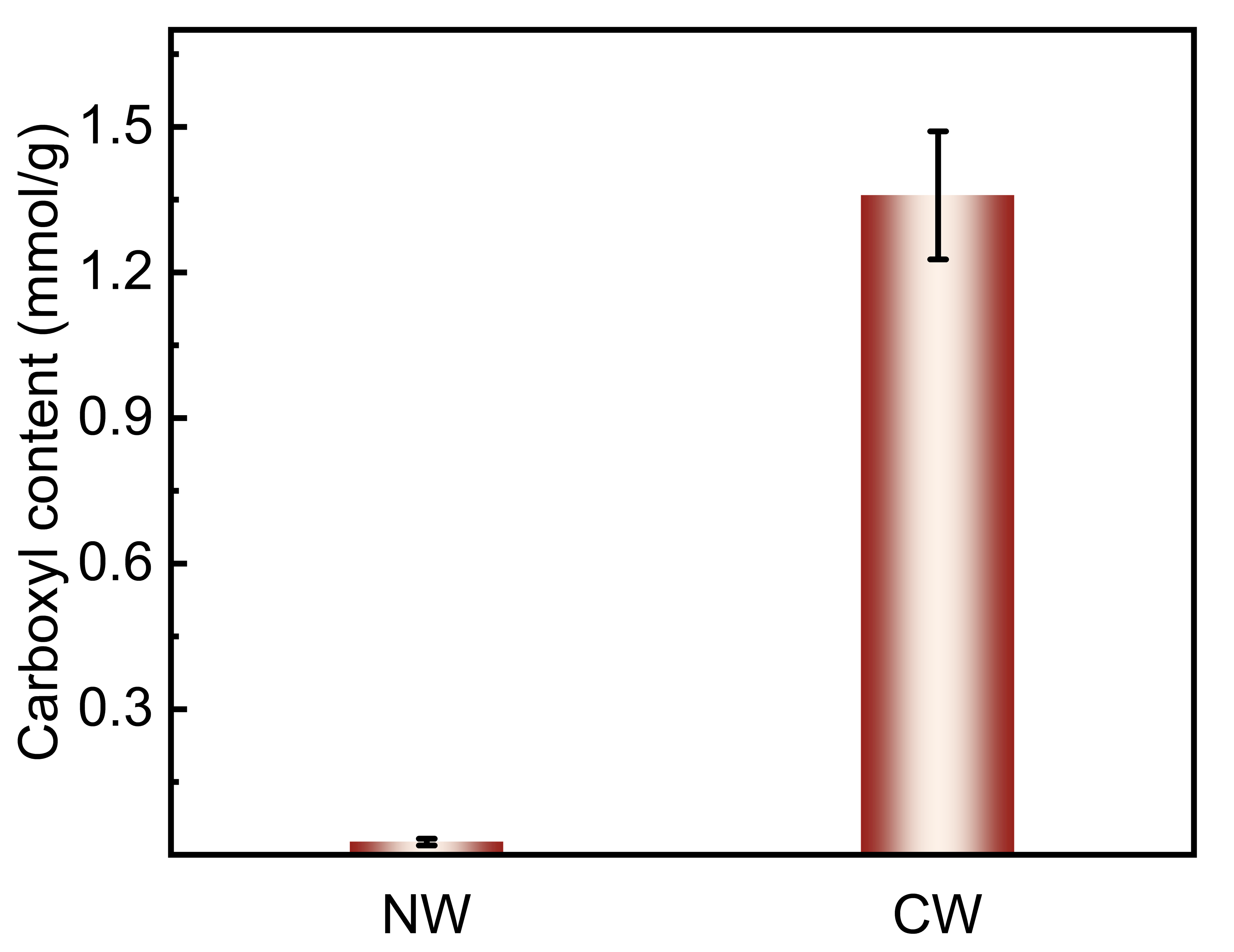

Supplement: Supplementary 1 — Figs. S1 to S39 Tables S1 to S9 Movies S1 to S3 [file research.1195.f1.zip › Supplementary Figures/Figure S6.png]

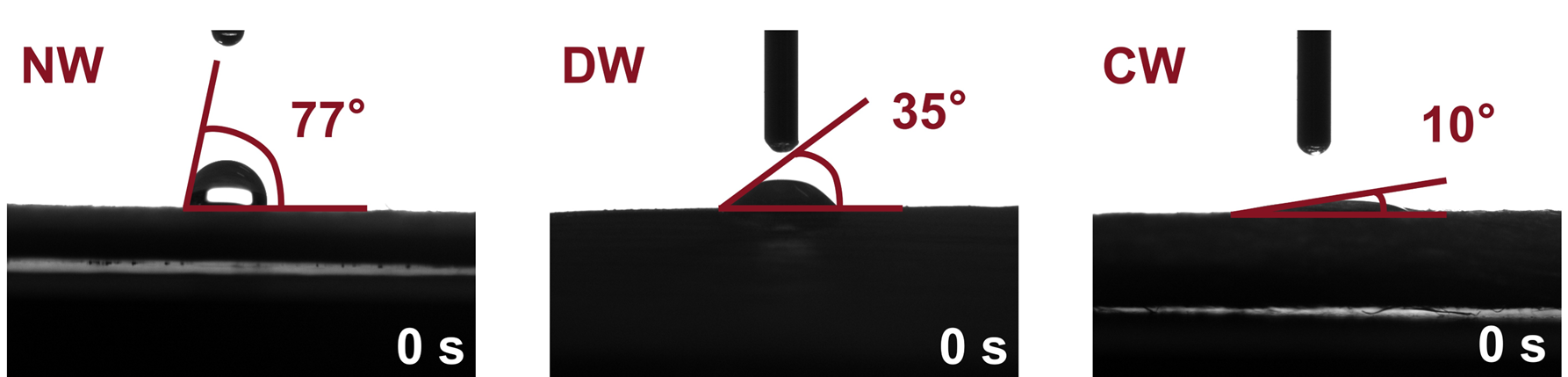

Supplement: Supplementary 1 — Figs. S1 to S39 Tables S1 to S9 Movies S1 to S3 [file research.1195.f1.zip › Supplementary Figures/Figure S7.png]

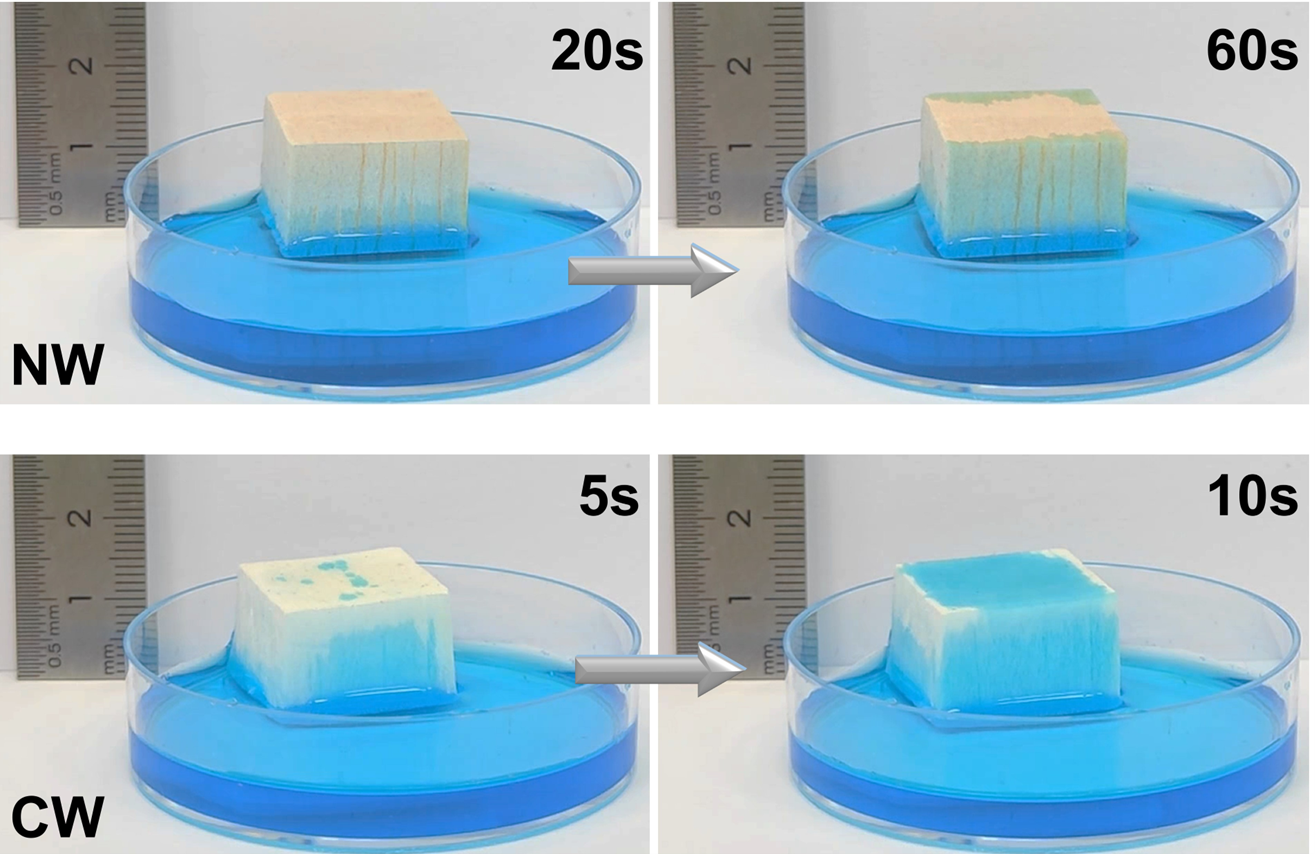

Supplement: Supplementary 1 — Figs. S1 to S39 Tables S1 to S9 Movies S1 to S3 [file research.1195.f1.zip › Supplementary Figures/Figure S8.png]

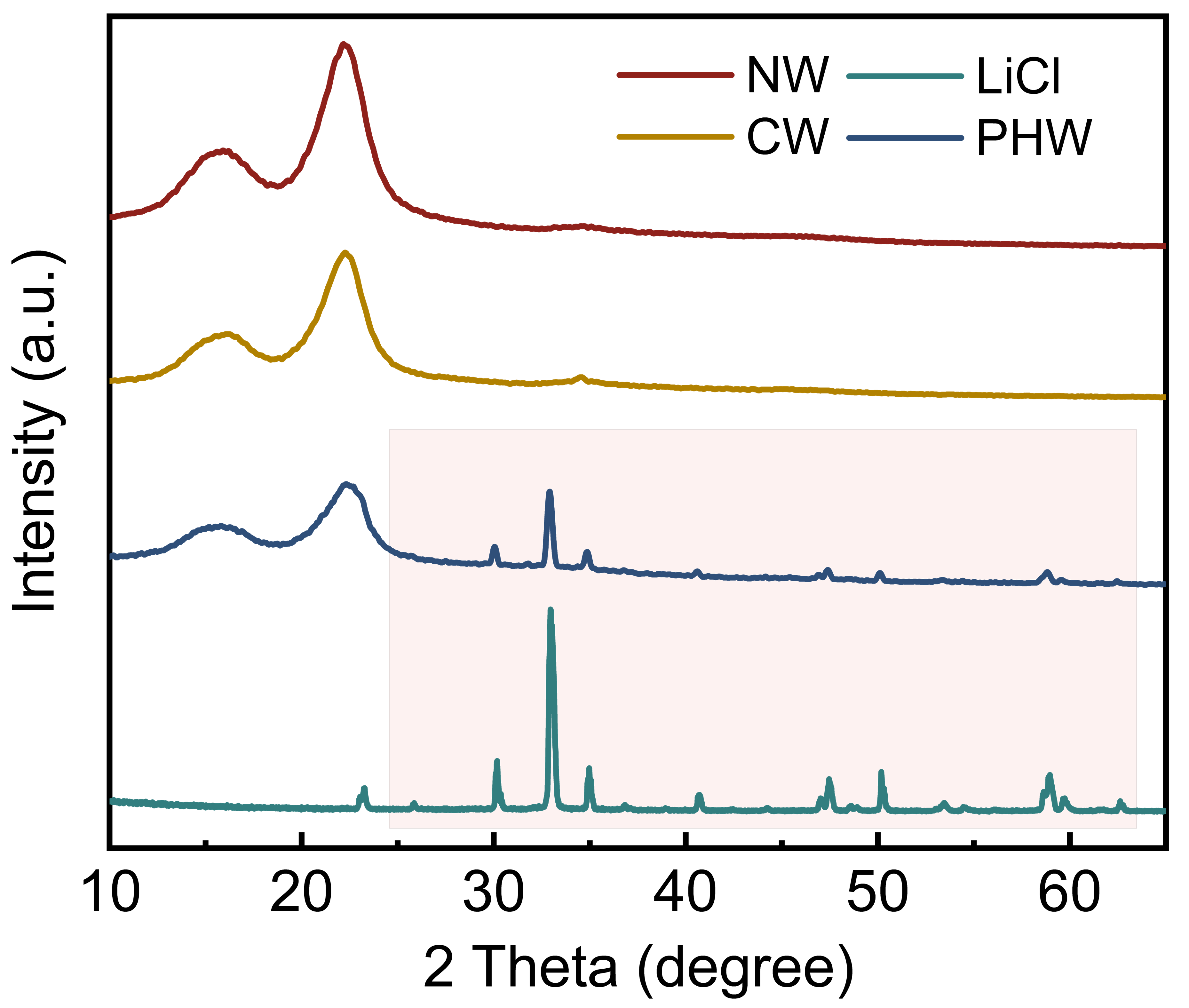

Supplement: Supplementary 1 — Figs. S1 to S39 Tables S1 to S9 Movies S1 to S3 [file research.1195.f1.zip › Supplementary Figures/Figure S9.png]
